# Supplementary material for: Dye-incorporated coordination polymers for direct photocatalytic trifluoromethylation of aromatics at metabolically susceptible positions
Source: Nat Commun. 2018 Oct 2;9:4024. doi: 10.1038/s41467-018-05919-6 (PMC6168478; doi:10.1038/s41467-018-05919-6)
Supplement: Supplementary file 1 — Supplementary Information [file 41467_2018_5919_MOESM1_ESM.docx]

**Dye-incorporated Coordination Polymers for Direct Photocatalytic Trifluoromethylation of Aromatics at Metabolically Susceptible Positions**

Zhang et al.

Supplementary Methods.

Synthesis of Ligand H_3_TCTA^1^.

To a solution of NaOH (7.20 g, 0.18 mol) in 30 ml water on ice bath, 3.1 ml of Br_2_ (*ca.* 0.06 mol) were added dropwisely, then the cold bath was removed, and the reaction mixture was further stirred for 20 min at room temperature. The solution was transferred to a pressure-equalizing dropping funnel and was added dropwise into a solution of 1,1',1''-[nitrilo*tris*(4,1-phenylenethiene-5,2-diyl)]triethanone (3.34 g, 5.4 mmol) in 50 ml 1,4-dioxane at room temperature, and stirred for 5 h at 45 ^o^C. Then the mixture was put on ice-bath, a saturated aqueous solution of hydroxylamine-HCl was added to quench the excessive sodium hypobromite. Then the mixture was acidified with 1 mmol/mL HCl solution, the precipitate was filtered and dried under vacuum. The crude product was recrystallized from acetic acid to afford pure product H_3_**TCTA** (tris[4-(5-carboxy-2-thienyl)phenyl]amine) as a yellow powder. (2.42 g, 72%). ^1^H NMR (400 MHz, DMSO-*d*_6_) δ = 13.10 (br s, 3H, acid), 7.74–7.70 (m, 9H, Ph and thiophene), 7.51 (d, J = 3.9 Hz, 3H, thiophene), 7.15 (d, J = 8.7 Hz, 6H, Ph); ^13^C NMR (126 MHz, DMSO-*d*_6_) δ = 162.8, 149.4, 146.7, 134.4, 132.6, 128.0, 127.3, 124.4, 123.9. FTIR (KBr pellet): 3456, 2852, 1674, 1596, 1536, 1508, 1448, 1322, 1276, 1186, 1103, 1037, 812, 747 cm^-1^. HRMS (m/z) [M^+^] calcd. for C_33_H_21_NO_6_S_3_, 623.0531; found, 623.0535.

Synthesis of Zn−TCTA.

A mixture of H_3_**TCTA** (93 mg, 0.15 mmol), Zn(NO_3_)_2_∙6H_2_O (297 mg, 1.0 mmol) was dissolved in 6 mL DMF in a Teflon-lined steel autoclave. The resulting mixture was kept in an oven at 100 °C for 3 days. The block red-brown crystals for X-ray structural analysis were collected by filtration, washed with acetonitrile, and then dried under vacuum. Yield: 70%. ^1^H NMR (400 MHz, DMSO-*d*_6_/DCl): δ = 7.72–7.70 (m, 9H), 7.50 (d, J = 3.9 Hz, 3H), 7.13 (d, J = 8.7 Hz, 3H). Analysis (calcd., found for (Zn_4_O)(C_33_H_18_NO_6_S_3_)_2_): C (52.19, 52.02), H (2.39, 2.51), N (1.84, 1.96), S (12.67, 12.48). FTIR (KBr): 3400, 1596, 1534, 1447, 1398, 1321, 1291, 1185, 1119, 813, 768 cm^-1^.

Single-Crystal X-ray Crystallography of Photocatalyst and Related Host Guest Composites

Intensities were collected on a Bruker SMART APEX CCD diffractometer with graphite monochromated Mo-Kα radiation (*λ* = 0.71073 Å) using the SMART and SAINT programs**^2,3^**. The structure was solved by direct methods and refined on F2 by full-matrix least-squares methods with SHELXTL version 5.1**^4^**. Hydrogen atoms were fixed geometrically at calculated positions and allowed to ride on the parent non-hydrogen atoms. The SQUEEZE program was carried out for crystals Zn−**TCTA**, **1a**@Zn−**TCTA**, and **3a**@Zn−**TCTA**. Crystallographic data for Zn−**TCTA**, **1a**@Zn−**TCTA**, **3a**@Zn−**TCTA** and **3f**@Zn−**TCTA** are summarized in Supplementary Table 1. For all of the crystal data, one of the carboxylate oxygen atoms was disordered into two parts with the site occupancy factor (s.o.f.) of each part fixed at 0.5.

In the refinement of crystal data of **1a**@Zn−**TCTA**, **3a**@Zn−**TCTA**, and **3f**@Zn−**TCTA**, to help the stability of the refinement for the impregnated related substrate molecule, the bond distances between several atoms were fixed; the geometrical constraints of idealized regular polygons were used for benzene rings, and thermal parameters on adjacent atoms in two molecules were restrained to be similar. The A alert error in the checklist for **3f**@Zn−**TCTA** is due to the partial occupancy of the substrate molecules.

Dye Uptake Experiments.

Crystals of Zn−**TCTA** (6.0 μmol) were soaked in a saturated solution of methylene blue in acetonitrile for 12 h, and the resulting crystals were washed with acetonitrile thoroughly until the solution became clear. The dried sample was dissociated by concentrated hydrochloric acid, and the resultant clear solution with a light olivine colour was diluted to 10 mL and adjusted to a pH of 1.5. The dye concentration was determined by comparing the solution UV-vis absorption with a standard curve of the dye. The amount of methylene blue uptaken by Zn−**TCTA** was calculated to be 2.8 % (m %).

Substrate Ingress and Egress Experiments.

Crystals of Zn−TCTA (0.07 mmol) was soaked in a solution of substrate 1a (0.25 mmol) in acetonitrile (1 mL), and the mixture was shaken by a vortex reactor. The uptake amount of 1a was monitored by time-course sampling of supernatant and gas chromatography (GC). The previously obtained crystals saturated with substrate 1a was washed with a minimum amount of acetonitrile to remove the substrate absorbed on the surface, then immersed in acetonitrile (1 mL), and shaken by a vortex reactor. The release amount of 1a was time-course monitored by GC analysis.

Substrate-Product Exchange Experiments.

Crystals of Zn−TCTA (0.07 mmol) was soaked in a solution of product 2a (0.25 mmol) in acetonitrile (1 mL), and the mixture was shaken by a vortex reactor for *ca.* 0.5 h to reach the saturated adsorption of 2a in Zn−TCTA. The obtained crystals saturated with product 2a was washed with a minimum amount of acetonitrile to remove the product absorbed on the surface, then immersed in acetonitrile (1 mL) with specified amount of substrate 1a pre-dissolved inside (25%, 50%, 75%, and 100% of 0.25 mmol, respectively), and shaken by a vortex reactor for *ca.* 0.5 h to reach the equilibrium. The competitive sorption of 1a and 2a in Zn−TCTA was monitored by GC analysis on the amounts of 1a and 2a in solution phase.

Substrate Encapsulation Experiments.

The substrate-impregnated crystals were obtained by soaking crystals of Zn−TCTA in a solution of the substrate in acetonitrile (1 M) for 12 h. After the soaked Zn−TCTA was washed with acetonitrile, the substrate-loaded crystals were directly used for single-crystal X-ray diffraction and IR or digested with DMSO-*d*^6^/DCl, and the amounts of released substrate molecules were quantified by ^1^H NMR.

General Procedure (GP) for Photocatalyzed Trifluoromethylation by Zn−TCTA.

To a pre-dried Pyrex tube equipped with a cooling water system, specified amounts of Zn−**TCTA** crystals (0.00625 mmol) and substrate (0.25 mmol) were added. After adding acetonitrile (1 mL), 2,4,6-collidine (0.50 mmol), and TfCl (0.50 mmol) by syringe, the reaction mixture was stirred and illuminated with visible light by a 23 W household light for 24 h. The catalyst was recovered by centrifugation and filtration, and the filtrate was concentrated under reduced pressure. The product was isolated *via* flash chromatography on silica gel from the crude mixture.

In the case of photocatalytic perfluoroalkylation, the same mmol amount of C_4_F_9_SO_2_Cl was used instead of TfCl. In the case of gram-scale preparation of **2a**, the reaction was conducted according to GP but at a 15 mmol scale, and 2.42 g of **2a** was obtained (91% isolated yield). For the photoreactions catalysed by MOF−**150** or H_3_**TCTA**, the specified amounts of corresponding catalysts will be used instead of Zn−**TCTA**.

Typical Procedure for 10 Consecutive Runs of Photocatalysis.

To a pre-dried Pyrex tube equipped with a cooling water system, specified amounts of photocatalyst Zn−**TCTA** (0.00625 mmol) and substrate **1a** (2.5 mmol) were added. After adding acetonitrile, 2,4,6-collidine (0.50 mmol) and TfCl (0.50 mmol) were added by syringe, and the reaction mixture was stirred and illuminated with visible light by a 23 W household light for 24 h. Then, another 0.5 mmol of additive base and TfCl were added, and the reaction was intermittently charged with the previously mentioned reagents for a total of 10 times. When *fac*-Ir(Fppy)^3^ was used as the photocatalyst, the reaction was performed according to the literature protocol**^6^**, except for the use of 10 eq. of **1a** (2.5 mmol) and the intermittently charged additive base and TfCl.

Characterization of the Substrates and Photocatalytic Products.

4-Nonylphenyl 2-(1-(4-chlorobenzoyl)-5-methoxy-2-methyl-1*H*-indol-3-yl)acetate^5^

1p

This compound was synthesized is prepared according to the literature protocol, and isolated by column chromatography as sticky oil (75% yield) using petroleum ether/ethyl acetate (10:1 v:v) as the eluent system. ^1^H NMR (500 MHz, CDCl_3_): δ 7.68 (d, J = 8.4 Hz, 2H), 7.47 (d, J = 8.5 Hz, 2H), 7.30 (dd, J = 14.7, 8.8 Hz, 1H), 7.23 (dd, J = 11.6, 6.7 Hz, 1H), 7.06 (s, 1H), 7.01 – 6.95 (m, 2H), 6.89 (dd, J = 9.0, 2.2 Hz, 1H), 6.69 (dd, J = 9.0, 2.2 Hz, 1H), 3.89 (s, 2H), 3.84 (s, 3H), 2.45 (s, 3H), 1.73 – 0.50 (m, 19H). ^13^C NMR (101 MHz, CDCl_3_): δ 169.6, 168.5, 156.4, 139.5, 136.4, 134.1, 131.4, 131.1, 130.8, 129.4, 127.6, 127.2, 120.7, 120.6, 115.2, 112.4, 112.0, 101.4, 56.0, 38.1, 33.4, 30.8, 30.2, 29.9, 29.4, 29.3, 28.8, 22.1, 14.4, 13.7.

1-Methyl-3-(trifluoromethyl)pyridin-2(1*H*)-one^6^

2a

This compound was synthesized according to the general procedure (GP) and isolated by column chromatography as clear oil (37 mg, 84% yield) using petroleum ether/ethyl acetate (10:1 v:v) as the eluent system. ^1^H NMR (400 MHz, CDCl_3_): δ 7.75 (dd, J = 7.1 Hz, 1H), 7.54 (dd, J = 6.6 Hz, 1H), 6.24 (t, J = 6.9 Hz, 1H), 3.60 (s, 3H). ^13^C NMR (101 MHz, CDCl_3_): δ 159.0, 142.4, 139.0 (q, J = 5.1 Hz), 122.9 (q, J = 271.5 Hz), 120.5 (q, J = 30.8 Hz), 104.1, 38.0. ^19^F NMR (470 MHz, CDCl_3_): δ -66.0.

1-Methyl-3-(perfluorobutyl)pyridin-2(1*H*)-one

2aa

This compound was synthesized according to the general procedure (GP) and isolated by column chromatography as clear oil (71 mg, 87% yield) using petroleum ether/ethyl acetate (15:1 v:v) as the eluent system. ^1^H NMR (500 MHz, CDCl_3_):: δ 7.72 (dd, J = 7.2 and 1.6 Hz, 1H), 7.60 (d, J = 6.4 Hz, 1H), 6.28 (t, J = 7.0 Hz, 1H), 3.60 (s, 3H). ^13^C NMR (126 MHz, CDCl_3_):: δ 159.0 (t, J = 2.2 Hz), 143.1, 141.9 (t, J = 8.6 Hz), 121.1 – 106.5 (m), 118.8 (t, J = 22.5 Hz), 104.3, 38.2. ^19^F NMR (470 MHz, CDCl_3_):: δ -81.05 (t, J = 9.9 Hz, 3F), -110.95 (t, J = 13.3 Hz, 2F), -121.76 – -121.89 (m, 2H), -126.03 – -126.14 (m, 2H). FTIR (film): 3088, 3046, 3008, 2931, 1665, 1598, 1556, 1483, 1435, 1409, 1379, 1352, 1306, 1272, 1197, 1132, 1095, 1016, 961, 913, 886, 872, 808, 765, 745, 727, 700, 657, 641, 620, 605, 577, 540, 495, 474 cm^-1^. HRMS (m/z) [M^+^] calcd. for C_10_H_6_F_9_NO, 327.0306; found, 327.0307.

1,3-Dimethyl-5-(trifluoromethyl)pyrimidine-2,4(1*H*,3*H*)-dione^6^

2b

This compound was synthesized according to the GP and isolated by column chromatography as clear oil (47 mg, 90% yield) using petroleum ether/ethyl acetate (5:1 v:v) as the eluent system. ^1^H NMR (500 MHz, CDCl_3_): δ 7.71 (s, 1H), 3.50 (s, 3H), 3.37 (s, 3H). ^13^C NMR (126 MHz, CDCl_3_): δ 158. 9, 151.1, 143.8 (q, J = 5.8 Hz), 122.2 (q, J = 269.8 Hz), 104.2 (q, J = 33.0 Hz), 37.8, 28.1. ^19^F NMR (470 MHz, CDCl_3_): δ -63.8.

2,4,6-Trimethoxy-5-(trifluoromethyl)pyrimidine^6^

2c

This compound was synthesized according to the GP and isolated by column chromatography as pale solid (55 mg, 92% yield) using petroleum ether/ethyl acetate (20:1 v:v) as the eluent system. ^1^H NMR (500 MHz, CDCl_3_): δ 4.03 (s, 6H), 4.01 (s, 3H). ^13^C NMR (126 MHz, CDCl_3_): δ 170.1, 165.2, 123.7 (q, J = 271.3 Hz), 89.6 (q, J = 34.0 Hz), 55.3, 55.1. ^19^F NMR (470 MHz, CDCl_3_): δ -56.0.

3,7-Dimethyl-1-(5-oxohexyl)-8-(trifluoromethyl)-*1H*-purine-2,6(3*H*,7*H*)-dione^7^2d

This compound was synthesized according to the GP and isolated by column chromatography as pale solid (75 mg, 87% yield) using petroleum ether/ethyl acetate (10:1 v:v) as the eluent system. ^1^H NMR (400 MHz, CDCl_3_): δ 4.16 (s, 3H), 4.02 (t, J = 6.7 Hz, 2H), 3.58 (s, 3H), 2.51 (t, J = 6.7 Hz, 2H), 2.15 (s, 3H), 1.68 – 1.62 (m, 4H). ^13^C NMR (101 MHz, CDCl_3_): δ 208.7, 155.4, 151.2, 146.7, 139.0 (q, J = 40.0 Hz), 118.3 (q, J = 271.3 Hz), 109.8, 43.2, 41.2, 33.3 (q, J = 2.0 Hz), 30.0, 29.9, 27.4, 21.0. ^19^F NMR (470 MHz, CDCl_3_): δ -62.6.

7-(2-Chloroethyl)-1,3-dimethyl-8-(trifluoromethyl)-1*H*-purine-2,6(3*H*,7*H*)-dione

2e

This compound was synthesized according to the GP and isolated by column chromatography as sticky oil (32 mg, 41% yield) using petroleum ether/ethyl acetate (15:1 v:v) as the eluent system. In the case of using 3.0 equiv. of TfCl and collidine, the target compound was obtained in a 61% yield (47 mg). ^1^H NMR (400 MHz, CDCl_3_): δ 4.77 (t, J = 6.4 Hz, 2H), 3.94 (t, J = 6.4 Hz, 2H), 3.61 (s, 3H), 3.43 (s, 3H). ^13^C NMR (126 MHz, CDCl_3_): δ 155.3, 151.4, 147.3, 139.5 (q, J = 40.1 Hz), 118.3 (q, J = 271.8 Hz), 109.2, 48.1 (q, J = 1.8 Hz), 41.9, 30.2, 28.5. ^19^F NMR (470 MHz, CDCl_3_): δ -60.6. FTIR (film): 2960, 1705, 1670, 1552, 1457, 1423, 1350, 1294, 1178, 1147, 1053, 981, 968, 767, 750, 669 cm^-1^. HRMS (m/z) [M+H^+^] calcd. for C_10_H_11_ClF_3_N_4_O_2_, 311.0517; found, 311.0516.

3-(Trifluoromethyl)-2*H*-chromen-2-one^8^

2f

This compound was synthesized according to the GP and isolated by column chromatography as white solid (33 mg, 62% yield) using petroleum ether/ethyl acetate (7:1 v:v) as the eluent system. ^1^H NMR (500 MHz, CD_3_CN) δ 8.41 (s, 1H), 7.77 – 7.72 (m, 2H), 7.44 – 7.41 (m, 2H). ^13^C NMR (126 MHz, CD_3_CN) δ 157.2, 155.8, 145.6 (q, J = 4.8 Hz), 135.7, 131.2, 126.3, 123.0 (q, J = 270.7 Hz), 118.2 (overlapped with CN peak of CD_3_CN), 117.9, 117.6. ^19^F NMR (470 MHz, CDCl_3_): δ -67.2.

3-Methyl-2-(trifluoromethyl)benzofuran^9^

2g

This compound was synthesized according to the GP and isolated by column chromatography as clear sticky oil (38 mg, 76% yield) using light petroleum ether/ether (100:1 v:v) as the eluent system. ^1^H NMR (500 MHz, CDCl_3_): δ 7.60 (d, J = 7.8 Hz, 1H), 7.52 (d, J = 8.3 Hz, 1H), 7.43 (t, J = 7.7 Hz, 1H), 7.33 (dd, J = 11.1 and 4.0 Hz, 1H), 2.41 (dd, J = 3.9 and 1.9 Hz, 3H). ^13^C NMR (126 MHz, CDCl_3_): δ 154.2, 138.7 (q, J = 39.7 Hz), 128.6, 127.1, 123.6, 120.9, 120.7 (q, J = 268.7 Hz), 118.4 (q, J = 2.7 Hz), 112.1, 7.88 (q, J = 0.6 Hz). ^19^F NMR (470 MHz, CDCl_3_): δ -62.03 (d, J = 2.0 Hz).

3-Methyl-2-(trifluoromethyl)benzo[*b*]thiophene^9^

2h

This compound was synthesized according to the GP and isolated by column chromatography as clear sticky oil (39 mg, 72% yield) using light petroleum ether as the eluent system. ^1^H NMR (500 MHz, CDCl_3_): δ 7.88 – 7.84 (m, 1H), 7.82 – 7.78 (m, 1H), 7.49 – 7.44 (m, 2H), 2.57 (d, J = 1.7 Hz, 3H). ^13^C NMR (101 MHz, CDCl_3_): δ 139.8, 138.8 (d, J = 0.7 Hz), 134.9 (q, J = 3.4 Hz), 126.7, 125.0, 123.4 (d, J = 270.5 Hz), 123.2, 122.9, 122.8, 12.1. ^19^F NMR (470 MHz, CDCl_3_): δ -54.08 (d, J = 1.1 Hz).

1,4-Dimethoxy-2-(trifluoromethyl)benzene^6^

2i

This compound was synthesized according to the GP and isolated by column chromatography as clear oil (40 mg, 77% yield) using petroleum ether/diethyl ether (20:1 v:v) as the eluent system. ^1^H NMR (400 MHz, CDCl_3_): δ 7.11 (d, J = 2.9 Hz, 1H), 7.02 (dd, J = 9.0, 2.9 Hz, 1H), 6.94 (d, J = 9.0 Hz, 1H), 3.85 (s, 3H), 3.79 (s, 3H). ^13^C NMR (126 MHz, CDCl_3_): δ 153.2, 151.8 (q, J = 1.6 Hz), 123.6 (q, J = 272.5 Hz), 119.7 (d, J = 31.0 Hz), 118.3, 113.9, 113.1 (d, J = 5.4 Hz), 56.8, 56.1. ^19^F NMR (470 MHz, CDCl_3_): δ -62.4.

1,3,5-Trimethoxy-2-(trifluoromethyl)benzene^10^

2j

This compound was synthesized according to the GP and isolated by column chromatography as pale solid (54 mg, 91% yield) using petroleum ether/ethyl acetate (20:1 v:v) as the eluent system. ^1^H NMR (500 MHz, CDCl_3_): δ 6.13 (s, 2H), 3.83 (s, 9H). ^13^C NMR (126 MHz, CDCl_3_): δ 163.7, 160.6, 124.5 (q, J = 273.2 Hz), 100.6 (q, J = 30.2 Hz), 91.5, 56.5, 55.6. ^19^F NMR (470 MHz, CDCl_3_): δ -54.2.

1,2-Dimethoxy-4-methyl-5-(trifluoromethyl)benzene^6^

2k

This compound was synthesized according to the GP and isolated by column chromatography as clear oil (49 mg, 89% yield) using petroleum ether/diethyl ether (30:1 v:v) as the eluent system. ^1^H NMR (400 MHz, CDCl_3_): δ 7.04 (s, 1H), 6.71 (s, 1H), 3.87 (s, 3H), 3.85 (s, 3H), 2.39 (q, J = 1.8 Hz, 3H). ^13^C NMR (126 MHz, CDCl_3_): δ 151.1, 146.7, 130.0 (q, J = 1.9 Hz), 124.9 (q, J = 272.7 Hz), 120.9 (q, J = 30.3 Hz), 114.7, 109.4 (q, J = 5.7 Hz), 56.31, 56.12, 19.1 (q, J = 1.9 Hz). ^19^F NMR (470 MHz, CDCl_3_): δ -60.0.

3,4-Dimethoxy-5-(trifluoromethyl)benzaldehyde^6^

2l

This compound was synthesized according to the GP and isolated by flash chromatography as clear oil (46 mg, 79% yield, 10:1 r.r.) using petroleum ether/diethyl ether (20:1 v:v) as the eluent system, and the pure major regioisomer could be further separated by column chromatography using gradient eluents petroleum ether/diethyl ether (50:1 to 30:1 v:v). ^1^H NMR (500 MHz, CDCl_3_): δ 10.28 (q, J = 2.1 Hz, 1H), 7.63 (s, 1H), 7.19 (s, 1H), 4.01 (s, 3H), 3.99 (s, 3H). ^13^C NMR (126 MHz, CDCl_3_): δ 188.0 (q, J = 2.8 Hz), 153.2, 151.6, 127.7, 125.8 (q, J = 32.8 Hz, 1H), 124.0 (q, J = 274.0 Hz), 110.5, 108.6 (q, J = 5.9 Hz), 56.7, 56.5. ^19^F NMR (470 MHz, CDCl_3_): δ -53.6.

3-Methoxy-13-methyl-4-(trifluoromethyl)-7,8,9,11,12,13,15,16-octahydro-6*H*-cyclopenta[*a*]phenanthren-17(14*H*)-one

2m

This compound was synthesized according to the GP and isolated by flash chromatography as sticky oil (57 mg, 65% yield, 4:1 r.r.) using petroleum ether/ethyl acetate (20:1 v:v) as the eluent system, and the pure major and minor regioisomers could be further separated by preparative chromatography using petroleum ether/ethyl acetate (20:1 v:v). Major regioisomer (2k) (sticky oil, 44 mg): ^1^H NMR (400 MHz, CDCl_3_): δ 7.43 (d, J = 8.8 Hz, 1H), 6.86 (d, J = 8.8 Hz, 1H), 3.85 (s, 3H), 3.12 – 2.95 (m, 2H), 2.51 (dd, J = 18.8 and 8.7 Hz, 1H), 2.42 – 2.35 (m, 1H), 2.29 – 2.24 (s, 1H), 2.22 – 1.93 (m, 4H), 1.68 – 1.63 (m, 1H), 1.58 – 1.46 (m, 4H), 1.37 (ddd, J = 23.7 and 11.4 and 6.3 Hz, 1H), 0.92 (s, 3H). ^13^C NMR (126 MHz, CDCl_3_): δ 220.8, 157.1, 138.0, 133.6, 130.1, 125.5 (q, J = 276.6 Hz), 117.2 (q, J = 28.3 Hz), 110.7, 56.6, 50.6, 48.1, 44.7, 37.4, 36.1, 31.8, 27.4 (q, J = 5.0 Hz), 26.6, 26.5 (q, J = 1.2 Hz), 21.7, 14.0. FTIR (film): 2935, 2862, 1740, 1597, 1581, 1483, 1440, 1286, 1240, 1138, 1115, 1072, 733 cm^-1^. ^19^F NMR (470 MHz, CDCl_3_): δ -53.2. HRMS (m/z) [M+H^+^] calcd. for C_20_H_24_F_3_O_2_, 353.1723; found, 353.1727.

3-Methoxy-13-methyl-2-(trifluoromethyl)-7,8,9,11,12,13,15,16-octahydro-6*H*-cyclopenta[*a*]phenanthren-17(14*H*)-one

2m'

Minor regioisomer (2k') (sticky oil, 10 mg): ^1^H NMR (400 MHz, CDCl_3_): δ 7.46 (s, 1H), 6.71 (s, 1H), 3.86 (s, 3H), 2.95 – 2.94 (m, 2H), 2.51 (dd, J = 18.8 and 8.6 Hz, 1H), 2.44 – 2.40 (m, 1H), 2.27 – 1.97 (m, 5H), 1.69 – 1.41 (m, 6H), 0.92 (s, 3H). ^13^C NMR (126 MHz, CDCl_3_): δ 220.7, 155.5, 142.4, 131.8, 124.4 (q, J = 5.2 Hz), 124.2 (q, J = 272.2 Hz), 116.5 (q, J = 30.5 Hz), 112.6, 56.2, 50.6, 48.2, 43.9, 38.4, 36.0, 31.7, 30.0, 26.5, 26.0, 21.8, 14.0. FTIR (film): 2933, 2868, 1740, 1622, 1578, 1508, 1466, 1416, 1336, 1298, 1255, 1192, 1153, 1124, 1052, 904, 733 cm^-1^. ^19^F NMR (470 MHz, CDCl_3_): δ -61.8. HRMS (m/z) [M+H^+^] calcd. for C_20_H_24_F_3_O_2_, 353.1723; found, 353.1728.

Methyl 2-(4-isobutyl-3-(trifluoromethyl)phenyl)propanoate

2n

This compound was synthesized according to the GP and isolated by flash chromatography as clear oil (52 mg, 72% yield) using gradient eluents petroleum ether/ethyl acetate (80:1 to 30:1 v:v). ^1^H NMR (400 MHz, CDCl_3_): δ 7.53 (s, 1H), 7.39 (d, J = 8.1 Hz, 1H), 7.26 (d, J = 7.9 Hz, 1H), 3.74 (q, J = 7.1 Hz, 1H), 3.68 (s, 3H), 2.63 (d, J = 7.2 Hz, 2H), 1.94 (tt, J = 13.8 and 6.8 Hz, 1H), 1.51 (d, J = 7.2 Hz, 3H), 0.93 (d, J = 6.6 Hz, 6H). ^13^C NMR (126 MHz, CDCl_3_): δ 174.7, 138.4, 132.1, 130.5, 129.2 (q, J = 29.3 Hz), 125.4 (q, J = 5.8 Hz), 124.8 (q, J = 274.2 Hz), 52.4, 45.1, 41.3 (q, J = 1.3 Hz), 30.0, 22.7, 18.7. ^19^F NMR (470 MHz, CDCl_3_): δ -58.9. FTIR (film): 2958, 2937, 2872, 1740, 1504, 1464, 1435, 1379, 1317, 1255, 1200, 1165, 1140, 1122, 1055, 901, 855, 852, 667 cm^-1^. HRMS (m/z) [M+H^+^] calcd. for C_15_H_20_F_3_O_2_, 289.1410; found, 289.1414.

Methyl 2-(1-(4-chlorobenzoyl)-5-methoxy-2-methyl-4-(trifluoromethyl)-1*H*-indol-3-yl)acetate

2o

This compound was synthesized according to the GP and isolated by flash chromatography as sticky oil (64 mg, 58% yield, 6:1 r.r.) using petroleum ether/ethyl acetate (15:1 v:v) as the eluent system, and the pure major and minor regioisomers could be further separated by column chromatography using gradient eluents petroleum ether/ethyl acetate (40:1 to 20:1 v:v). Major regioisomer (2m) (sticky oil, 52 mg): ^1^H NMR (500 MHz, CDCl_3_): δ 7.69 – 7.64 (m, 2H), 7.50 – 7.47 (m, 2H), 7.38 (d, J = 9.2 Hz, 1H), 6.84 (d, J = 9.2 Hz, 1H), 3.87 (s, 3H), 3.79 (q, J = 2.3 Hz, 2H), 3.72 (s, 3H), 2.26 (s, 3H). ^13^C NMR (126 MHz, CDCl_3_): δ 172.0, 168.3, 155.1, 140.3, 139.3, 133.6, 132.3, 131.7, 129.6, 124.8 (q, J = 273.4 Hz), 118.2, 111.3, 109.9, 109.1 (q, J = 31.8 Hz), 58.0, 52.2, 31.6 (q, J = 6.8 Hz), 13.9. ^19^F NMR (470 MHz, CDCl_3_): δ -51.9. FTIR (film): 3008, 2956, 2848, 1741, 1689, 1589, 1466, 1425, 1400, 1354, 1315, 1252, 1192, 1170, 1090, 1062, 1014, 925, 906, 845, 806, 752, 735 cm^-1^. HRMS (m/z) [M+H^+^] calcd. for C_21_H_18_ClF_3_NO_4_, 440.0871; found, 440.0880.

Methyl 2-(1-(4-chlorobenzoyl)-5-methoxy-2-methyl-6-(trifluoromethyl)-1*H*-indol-3-yl)acetate

2o'

Minor regioisomer (2m') (sticky oil, 8 mg): ^1^H NMR (500 MHz, CDCl_3_): δ 7.67 – 7.62 (m, 2H), 7.52 – 7.47 (m, 2H), 7.35 (s, 1H), 7.05 (s, 1H), 3.95 (s, 3H), 3.71 (s, 3H), 3.69 (s, 2H), 2.35 (s, 3H). ^13^C NMR (126 MHz, CDCl_3_): δ 171.2, 168.2, 154.2, 140.0, 138.3, 133.5, 133.2, 131.3, 129.5, 124.1 (q, J = 272.0 Hz), 115.0 (q, J = 30.8 Hz), 113.4 (q, J = 6.1 Hz), 112.4, 101.1, 56.6, 52.4, 30.3, 13.7. ^19^F NMR (470 MHz, CDCl_3_): δ -61.7. FTIR (film): 3004, 2952, 2935, 2841, 1740, 1689, 1628, 1597, 1473, 1433, 1402, 1360, 1329, 1272, 1170, 1153, 1124, 1090, 1053, 1034, 1014, 943, 841, 754, 737 cm^-1^. HRMS (m/z) [M+H^+^] calcd. for C_21_H_18_ClF_3_NO_4_, 440.0871; found, 440.0871.

1,3-Dimethyl-3-(2,2,2-trifluoroethyl)indolin-2-one^11^

4a

This compound was synthesized according to the GP and isolated by flash chromatography as clear sticky oil (57 mg, 93% yield) using eluent petroleum ether/ethyl acetate (7:1 v:v). ^1^H NMR (500 MHz, CDCl_3_): δ = 7.31 (td, J = 7.7 and 1.2 Hz, 1H), 7.26 (d, J = 7.3 Hz, 1H), 7.09 (td, J = 7.6 and 0.8 Hz, 1H), 6.88 (d, J = 7.8 Hz, 1H), 3.23 (s, 3H), 2.82 (dq, J = 15.1 and 10.8 Hz, 1H), 2.65 (dq, J = 15.2 and 10.5 Hz, 1H), 1.41 (s, 3H); ^13^C NMR (126 MHz, CDCl_3_): δ = 178.6, 143.0, 131.2, 128.7, 125.4 (q, J = 278.0 Hz), 123.7, 122.8, 120.5 – 112.3, 108.6, 44.5 (d, J = 1.7 Hz), 40.8 (q, J = 28.3 Hz), 26.6, 25.1; ^19^F NMR (470 MHz, CDCl_3_): δ = -62.0 (t, J = 10.7 Hz).

1,3-Dimethyl-3-(2,2,3,3,4,4,5,5,5-nonafluoropentyl)indolin-2-one^12^

4aa

This compound was synthesized according to the GP and isolated by flash chromatography as clear sticky oil (89 mg, 91% yield) using eluent petroleum ether/ethyl acetate (10:1 v:v). ^1^H NMR (400 MHz, CDCl_3_): δ 7.32 (td, J = 7.8, 1.1 Hz, 1H), 7.29 (d, J = 7.5 Hz, 1H), 7.10 (td, J = 7.6 and 0.7 Hz, 1H), 6.90 (d, J = 7.8 Hz, 1H), 3.25 (s, 3H), 2.88 (dd, J = 35.2 and 15.3 Hz, 1H), 2.61 (ddd, J = 31.0 and 15.4 and 8.1 Hz, 1H), 1.44 (s, 3H). ^13^C NMR (126 MHz, C_6_D_6_) δ 178.8, 143.0, 131.5, 128.7, 123.8, 122.8, 108.7, 44.4 (d, J = 1.8 Hz), 37.1 (t, J = 20.5 Hz), 26.7, 26.1. ^19^F NMR (470 MHz, C_6_D_6_) δ -81.2 (t, J = 9.5 Hz, 3F), -108.6 – -109.6 (m, 1F), -114.3 – -115.2 (m, 1F), -124.7 (dd, J = 19.4 and 14.1 Hz, 2F), -125.9 – -125.9 (m, 1F), -126.0 – -126.1 (m, 1F).

2,4-Dimethyl-4-(2,2,2-trifluoroethyl)isoquinoline-1,3(2*H*,4*H*)-dione^13^

4b

This compound was synthesized according to the GP and isolated by flash chromatography as clear oil (43 mg, 64% yield) using eluent petroleum ether/ethyl acetate (7:1 v:v). ^1^H NMR (500 MHz, CDCl_3_): δ = 8.29 (dd, J = 7.9 and 1.2 Hz, 1H), 7.67 (td, J = 7.8 and 1.4 Hz, 1H), 7.49 (td, J = 8.0 and 1.0 Hz, 1H), 7.43 (d, J = 7.9 Hz, 1H), 3.44 – 3.30 (m, 4H), 2.81 (dq, J = 15.1 and 9.8 Hz, 1H), 1.66 (s, 3H); ^13^C NMR (126 MHz, CDCl_3_): δ = 174.8, 163.9, 140.6, 134.0, 129.5, 128.2, 125.8, 125.2 (q, J = 279.3 Hz), 124.4, 44.6 (q, J = 27.6 Hz), 43.8 (q, J = 2.1 Hz), 31.4, 27.6; ^19^F NMR (470 MHz, CDCl_3_): δ = -61.7 (t, J = 10.1 Hz).

1,3-Dimethyl-3-(2,2,2-trifluoroethyl)-1*H*-pyrrolo[3,2-*b*]pyridin-2(3*H*)-one^14^

4c

This compound was synthesized according to the GP and isolated by flash chromatography as clear sticky oil (51 mg, 84% yield) using eluent petroleum ether/ethyl acetate (4:1 v:v). ^1^H NMR (500 MHz, CDCl_3_): δ = 8.26 (dd, J = 5.1 and 1.1 Hz, 1H), 7.21 (dd, J = 7.9 and 5.1 Hz, 1H), 7.12 (dd, J = 7.9 and 1.1 Hz, 1H), 3.26 (s, 3H), 2.93 – 2.83 (m, 2H), 1.46 (s, 3H); ^13^C NMR (126 MHz, CDCl_3_): δ = 177.0, 152.4, 143.3, 138.3, 125.2 (q, J = 278.4 Hz), 123.3, 114.7, 44.8 (d, J = 2.0 Hz), 40.0 (q, J = 28.7 Hz), 26.3, 23.6; ^19^F NMR (470 MHz, CDCl_3_): δ = -62.8 (t, J = 10.4 Hz).

2,4-Dimethyl-7-nitro-4-(2,2,2-trifluoroethyl)isoquinoline-1,3(2*H*,4*H*)-dione^15^

4d

This compound was synthesized according to the GP and isolated by flash chromatography as slightly yellow sticky oil (57 mg, 72% yield) using eluent petroleum ether/ethyl acetate (4:1 v:v). ^1^H NMR (500 MHz, CDCl_3_): δ = 9.12 (d, J = 2.5 Hz, 1H), 8.49 (dd, J = 8.7 and 2.5 Hz, 1H), 7.66 (d, J = 8.7 Hz, 1H), 3.53 – 3.39 (m, 4H), 2.86 (dq, J = 15.3 and 9.6 Hz, 1H), 1.72 (s, 3H); ^13^C NMR (126 MHz, CDCl_3_): δ = 173.4, 161.9, 147.9, 146.7, 128.0, 127.7, 126.1, 124.93, 124.89 (q, J = 279.7 Hz), 44.5 (q, J = 28.4 Hz), 44.2 – 44.1 (m, partially overlapped), 31.1, 28.0; ^19^F NMR (470 MHz, CDCl_3_): δ = -61.8 (t, J = 9.9 Hz).

2,4-Dimethyl-4-(2,2,2-trifluoroethyl)-7-(trifluoromethyl)isoquinoline-1,3(2*H*,4*H*)-dione

4e

This compound was synthesized according to the GP and isolated by flash chromatography as clear oil (57 mg, 67% yield) using eluent petroleum ether/ethyl acetate (10:1 v:v). ^1^H NMR (500 MHz, CDCl_3_): δ = 8.58 (s, 1H), 7.90 (dd, J = 8.3 and 1.6 Hz, 1H), 7.58 (d, J = 8.3 Hz, 1H), 3.47 – 3.36 (m, 4H), 2.83 (dq, J = 15.2 and 9.6 Hz, 1H), 1.69 (s, 3H); ^13^C NMR (126 MHz, CDCl_3_): δ = 173.9, 162.7, 144.2, 131.1 (q, J = 33.7 Hz), 130.3 (q, J = 3.3 Hz), 126.91 (q, J = 3.8 Hz, partially overlapped), 126.88 (overlapped), 125.2, 125.0 (q, J = 279.2 Hz), 123.5 (q, J = 273.2 Hz), 44.5 (q, J = 27.9 Hz), 44.0 (d, J = 2.2 Hz), 31.2, 27.8; ^19^F NMR (470 MHz, CDCl_3_): δ = -61.6 (t, J = 9.9 Hz), -63.0. HRMS (m/z) [M+Na^+^] calcd. for C_14_H_11_NO_2_F_6_Na, 362.0592; found, 362.0598.

2,4-Dimethyl-4-(1,1,1-trifluoropropan-2-yl)isoquinoline-1,3(2*H*,4*H*)-dione

4f

This compound was synthesized according to the GP and isolated by flash chromatography as slightly yellow oil (49 mg, 69% yield) using eluent petroleum ether/ethyl acetate (7:1 v:v). Only the peaks of major diastereomer were assigned here; ^1^H NMR (500 MHz, CDCl_3_): δ = 8.25 (d, J = 7.8 Hz, 1H), 7.65 (t, J = 7.6 Hz, 1H), 7.49 – 7.42 (m, 2H), 3.37 (s, 3H), 2.89 – 2.81 (m, 1H), 1.77 (s, 3H), 1.37 (d, J = 7.1 Hz, 3H); ^13^C NMR (126 MHz, CDCl_3_): δ = 173.8, 164.2, 142.4, 133.9, 129.2, 128.1, 127.1 (q, J = 282.2 Hz), 126.0, 125.2, 50.2 (q, J = 24.7 Hz), 46.6, 27.6, 26.2, 10.2 (q, J = 2.8 Hz); ^19^F NMR (470 MHz, CDCl_3_): δ = -66.7 (d, J = 8.8 Hz). FTIR (film): 2924, 2853, 1715, 1670, 1605, 1463, 1418, 1363, 1325, 1300, 1260, 1238, 1165, 1127, 1055, 799, 763, 704 cm^-1^. HRMS (m/z) [M+Na^+^] calcd. for C_14_H_14_NO_2_F_3_Na, 308.0874; found, 308.0882.

6-Methoxy-2,4-dimethyl-4-(1,1,1-trifluoropropan-2-yl)isoquinoline-1,3(2*H*,4*H*)-dione^4^

4g

This compound was synthesized according to the GP unless using 1.50 equiv. of TfCl and collidine, and isolated by flash chromatography as clear oil (53 mg, 67% yield) using eluent petroleum ether/ethyl acetate (7:1 v:v). Only the peaks of major diastereomer were assigned here; ^1^H NMR (500 MHz, CDCl_3_): δ = 8.20 (d, J = 8.8 Hz, 1H), 6.98 (dd, J = 8.8 and 2.4 Hz, 1H), 6.87 (d, J = 2.3 Hz, 1H), 3.90 (s, 3H), 3.35 (s, 3H), 2.91 – 2.79 (m, 1H), 1.75 (s, 3H), 1.35 (d, J = 7.2 Hz, 3H); ^13^C NMR (126 MHz, CDCl_3_): δ = 173.9, 164.1, 163.9, 144.6, 131.5, 127.1 (q, J = 282.0 Hz), 118.2, 113.5, 111.8, 55.8, 50.1 (q, J = 24.8 Hz), 46.9, 27.4, 26.4, 10.3 (q, J = 2.9 Hz); ^19^F NMR (470 MHz, CDCl_3_): δ = -66.3 (d, J = 8.8 Hz).

2'-Methyl-2-(trifluoromethyl)-1'*H*-spiro[cyclohexane-1,4'-isoquinoline]-1',3'(2'*H*)-dione

4h

This compound was synthesized according to the GP and isolated by flash chromatography as clear oil (41 mg, 53% yield) using eluent petroleum ether/ethyl acetate (10:1 v:v). In the case of using 3.0 equiv. of TfCl and collidine, the target compound was obtained in a 71% yield (55 mg). Only the peaks of major diastereomer were assigned here; ^1^H NMR (500 MHz, CDCl_3_): δ = 8.27 (d, J = 7.7 Hz, 1H), 7.66 (td, J = 7.8 and 1.0 Hz, 1H), 7.48 (d, J = 8.0 Hz, 1H), 7.44 (t, J = 7.6 Hz, 1H), 3.37 (s, 3H), 2.92 (dqd, J = 12.6 and 8.5 and 4.2 Hz, 1H), 2.53 (qd, J = 13.2 and 3.7 Hz, 1H), 2.21 – 2.06 (m, 2H), 2.02 – 1.95 (m, 2H), 1.88 (td, J = 14.0 and 4.1 Hz, 1H), 1.67 – 1.60 (m, 1H), 1.49 (qt, J = 13.5 and 4.0 Hz, 1H); ^13^C NMR (126 MHz, CDCl_3_): δ = 172.4, 164.1, 143.9, 134.0, 129.3, 127.6, 126.8 (q, J = 283.5 Hz), 125.5, 124.7, 50.5 (q, J = 24.3 Hz), 45.4 (d, J = 1.1 Hz), 41.4, 27.2, 24.4, 21.9 (q, J = 2.3 Hz), 20.8; ^19^F NMR (470 MHz, CDCl_3_): δ = -65.8 (d, J = 8.5 Hz); FTIR (film): 2936, 2860, 1714, 1671, 1604, 1463, 1418, 1360, 1336, 1302, 1268, 1203, 1173, 1153, 1143, 1097, 761, 748, 703, 689 cm^-1^; HRMS (m/z) [M+Na^+^] calcd. for C_16_H_16_NO_2_F_3_Na, 334.1031; found, 334.1021.

2'-Methyl-2-(trifluoromethyl)-1'*H*-spiro[cyclopentane-1,4'-isoquinoline]-1',3'(2'*H*)-dione

4i

This compound was synthesized according to the GP and isolated by flash chromatography as clear oil (42 mg, 57% yield) using eluent petroleum ether/ethyl acetate (10:1 v:v). In the case of using 3.0 equiv. of TfCl and collidine, the target compound was obtained in a 74% yield (55 mg). Only the peaks of major diastereomer were assigned here; ^1^H NMR (500 MHz, CDCl_3_): δ = 8.23 (dd, J = 7.9 and 1.2 Hz, 1H), 7.67 (td, J = 7.8 and 1.4 Hz, 1H), 7.48 – 7.42 (m, 2H), 3.37 (s, 3H), 3.01 – 2.90 (m, 1H), 2.82 – 2.74 (m, 1H), 2.46 – 2.30 (m, 3H), 2.20 (dt, J = 10.3 and 7.4 Hz, 1H), 2.14 – 2.06 (m, 1H); ^13^C NMR (126 MHz, CDCl_3_): δ = 174.2, 164.3, 143.0, 134.4, 129.0, 128.0, 126.2 (q, J = 281.0 Hz), 125.6, 125.4, 61.1 (q, J = 26.5 Hz), 53.5 (d, J = 1.0 Hz), 42.0, 27.6, 26.8 (d, J = 1.6 Hz), 24.7; ^19^F NMR (470 MHz, CDCl_3_): δ = -66.6 (d, J = 8.7 Hz); FTIR (film): 2958, 1715, 1671, 1604, 1462, 1419, 1364, 1333, 1303, 1274, 1162, 1116, 1034, 757, 702 cm^-1^; HRMS (m/z) [M+Na^+^] calcd. for C_15_H_14_NO_2_F_3_Na, 320.0874; found, 320.0867.

*tert*-Butyl 4-(trifluoromethyl)-2,3,4,4a-tetrahydro-1*H*-carbazole-9(9a*H*)-carboxylate

4j

This compound was synthesized according to the GP and isolated by flash chromatography as clear oil (44 mg, 51% yield) using eluent petroleum ether/ethyl acetate (10:1 v:v). In the case of using 3.0 equiv. of TfCl and collidine, the target compound was obtained in a 66% yield (56 mg). Only the peaks of major diastereomer were assigned here; ^1^H NMR (500 MHz, CDCl_3_): δ = 7.70 (br s, 1H), 7.22 (td, J = 7.8 and 0.8 Hz, 1H), 7.11 (d, J = 7.3 Hz, 1H), 7.01 (t, J = 7.5 Hz, 1H), 4.54 – 4.43 (m, 1H), 3.62 (d, J = 7.8 Hz, 1H), 3.02 – 2.90 (m, 1H), 2.16 – 2.09 (m, 1H), 1.97 (d, J = 11.5 Hz, 1H), 1.57 (s, 9H), 1.55 – 1.45 (m, 3H), 1.21 – 1.09 (m, 1H); ^13^C NMR (126 MHz, CDCl_3_): δ = 152.2, 141.6, 131.1, 128.6 (q, J = 281.4 Hz), 128.4, 122.8, 122.3, 116.0, 81.2, 59.0, 38.6 (q, J = 25.2 Hz), 38.2 (d, J = 1.8 Hz, partially overlapped), 28.7, 26.3, 20.6 (d, J = 2.1 Hz), 18.0; ^19^F NMR (470 MHz, CDCl_3_): δ = -68.0 (d, J = 10.9 Hz); FTIR (film): 2976, 2880, 1704, 1604, 1481, 1463, 1392, 1367, 1314, 1286, 1256, 1240, 1176, 1163, 1145, 1126, 1108, 1068, 1016, 859, 752, 699, 620 cm^-1^; HRMS (m/z) [M+Na^+^] calcd. for C_18_H_22_NO_2_F_3_Na, 364.1500; found, 364.1494.

Supplementary Tables.

Supplementary Table 1. Crystallographic data and structural refinements^a^

| entry | Zn−**TCTA** | **1a**@Zn−**TCTA** | **3a**@Zn−**TCTA** | **3f**@Zn−**TCTA** |
| --- | --- | --- | --- | --- |
| empirical formula | C_66_H_36_N_2_O_13_S_6_Zn_4_ | C_78_H_62_N_4_O_21_S_6_Zn_4_ | C_92_H_68_N_6_O_15_S_6_Zn_4_ | C_92_H_68_N_4_O_18_S_6_Zn_4_ |
| CCDC No. | 1407818 | 1546691 | 1415189 | 1415190 |
| M, g mol^-1^ | 1518.81 | 1845.16 | 1951.36 | 1971.34 |
| cryst syst | Hexagonal | Hexagonal | Hexagonal | Hexagonal |
| space group | *R*-3c | *R*-3c | *R*-3c | *R*-3c |
| *a*, Å | 20.809(1) | 20.844(2) | 20.801(2) | 20.852(2) |
| *b*, Å | 20.809(1) | 20.844(2) | 20.801(2) | 20.852(2) |
| *c*, Å | 56.62(1) | 54.72(1) | 54.36(1) | 54.18(1) |
| *V*, Å^3^ | 21230(2) | 20590(4) | 20370(5) | 20404(6) |
| Z | 6 | 6 | 6 | 6 |
| *D*_calcd_,  g cm^-3^ | 0.713 | 0.893 | 0.954 | 0.963 |
| *T*, K | 100(2) | 100(2) | 100(2) | 100(2) |
| rflns collected | 42434 | 38539 | 34517 | 31424 |
| unique rflns | 4172 | 4016 | 3922 | 3971 |
| *R*_int_ | 0.0637 | 0.0743 | 0.1993 | 0.1237 |
| *μ*, mm^-1^ | 0.788 | 0.825 | 0.835 | 0.835 |
| GOF | 1.030 | 1.000 | 1.003 | 1.030 |
| F(000) | 4596 | 5652 | 5988 | 6048 |
| *R*_1_^a^ (*I* > 2*σ*) | 0.0503 | 0.1025 | 0.1000 | 0.1027 |
| *wR*_2_^b^  (all data) | 0.2308 | 0.2986 | 0.2815 | 0.2798 |
| ^a^ *R*_1_ = ∑\|\|*F*o\| – \|*F*c\|\|/∑\|*F*o\|.  ^b^ *wR*_2_ = [∑*w*(*F*o^2^– *F*c^2^)^2^/∑*w*(*F*o^2^)^2^]^1/2^; *w* = 1/[*σ*^2^(*F*o^2^) + (*xP*)^2^ + *yP*], *P* = (*F*o^2^ + 2*F*c^2^)/3, where *x* = 0.1859, *y* = 0.0000 for Zn−**TCTA**; where *x* = 0.1550, *y* = 0.0000 for **1a**@Zn−**TCTA**; where *x* = 0.1380, *y* = 0.0000 for **3a**@Zn−**TCTA**; where *x* = 0.0150, *y* = 0.0000 for **3f**@Zn−**TCTA**. | | | | |

Supplementary Table 2. The control experiments, hot filtration test, and catalyst recycling study^a^

|  | | |
| --- | --- | --- |
| entry | Variants | Yield (%)^b^ |
| 1 | None | 84 |
| 2 | H_3_**TCTA** (2.5%) | 27 |
| 3 | Zn(NO_3_)_2_•6H_2_O (10.0%) + H_3_**TCTA** (2.5%) | 44 |
| 4 | MOF-150 (2.5% based upon amount of **TCA** ligand) | 26 |
| 5 | No Catalyst | <5 |
| 6 | Dark | 0 |
| 7 | Air | <5 |
| 8 | 3 h | 33 |
| 9 | 3 h first with Zn-**TCTA**, then remove photocatalyst | 37 |
| 10 | Zn-**TCTA** (2.5%) recycle round 1 | 88 |
| 11 | Zn-**TCTA** (2.5%) recycle round 2 | 85 |
| 12 | Zn-**TCTA** (2.5%) recycle round 3 | 81 |
| 13 | with 1 eq. of TEMPO added | 0 |
| 14 | with 1 eq. of acetone added | 82 |
| 15 | with 1 eq. of 2,5-dimethylhexa-2,4-diene added | 55 |
| 16 | Zn-**TCTA** (2.5%) grounded powder | 86 |
| 17 | UiO-67-Ir(ppyF)_2_ instead of Zn-**TCTA** as catalyst | _^c^ |
| 18 | **1a**@Zn-**TCTA** (in a ratio of *ca.* 2:1) used instead of catalyst and substrate | 95^d^ |
| ^a^ Reaction conditions: **1** (0.25 mmol), TfCl (0.50 mmol, 2.0 equiv), additive base (0.50 mmol, 2.0 equiv), Zn-**TCTA** (0.00625 mmol, 0.025 equiv), MeCN (1 mL), 23 W household light, N_2_ atmosphere, room temperature, 24 h.  ^b^ Isolated yields.  ^c^ A mixture of regioisomers and over-trifluoromethylated products was obtained.  ^d^ Reaction completed in 1 h. | | |

Supplementary Figures


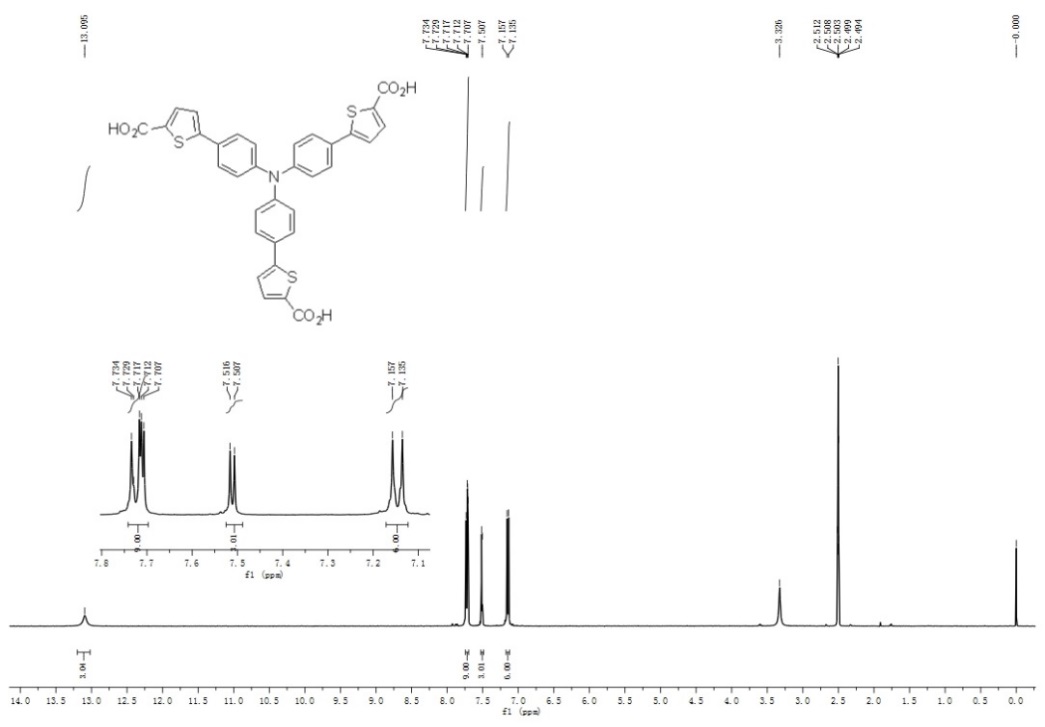


**Supplementary Figure 1.** ^1^H NMR spectra of H_3_**TCTA**.


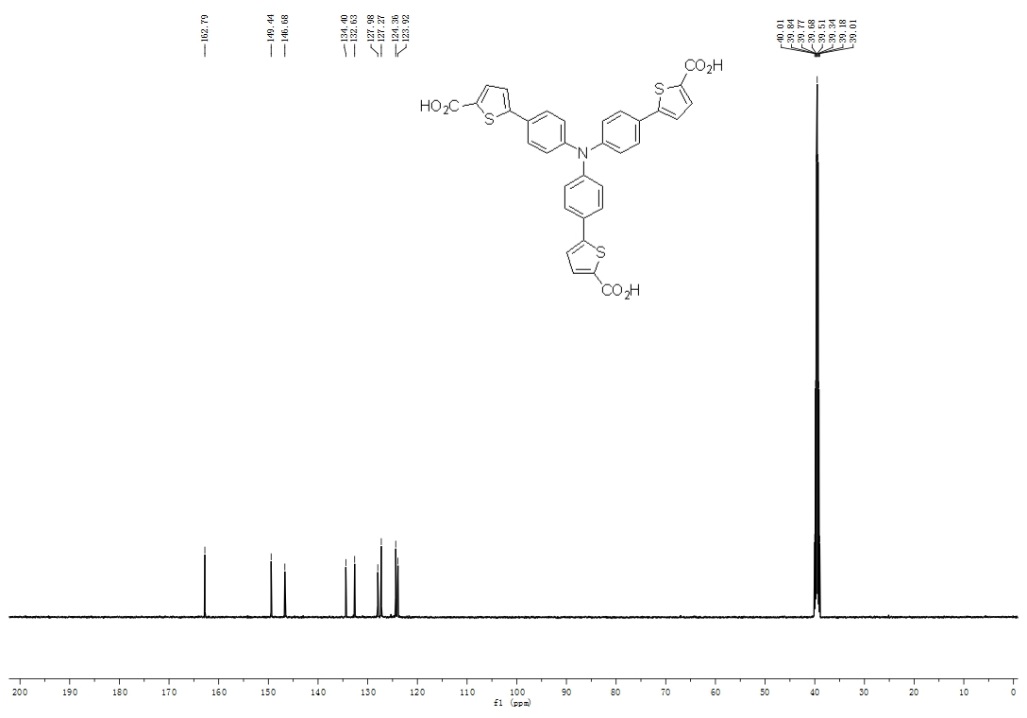


**Supplementary Figure 2.** ^13^C NMR spectra of H_3_**TCTA**.


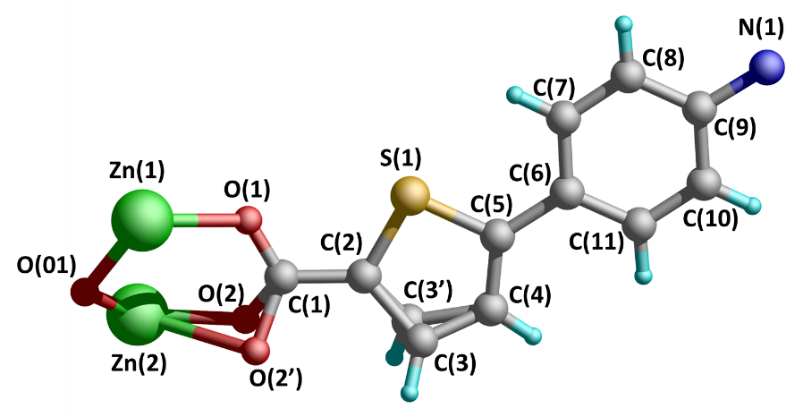


**Supplementary Figure 3.** The ball-and-stick diagram of Zn−**TCTA** in a unit with atomic-numbering scheme. **Selected bond lengths (Å):** Zn(1)−O(01) 1.951(1), Zn(1)−O(1) 2.146(3), Zn(2)−O(01) 1.867(1), Zn(2)−O(2) 1.961(4), Zn(2)−O(2’) 2.526(4), C(1)−O(1) 1.205(4), C(1)−O(2) 1.261(5), C(1)−O(2’) 1.385(5), C(1)−C(2) 1.449(5), S(1)−C(2) 1.701(3), S(1)−C(5) 1.698(3), C(2)−C(3) 1.513(9), C(2)−C(3’) 1.269(9), C(3)−C(4) 1.361(9), C(3’)−C(4) 1.538(9), C(4)−C(5) 1.356(5), C(5)−C(6) 1.475(4), C(6)−C(7) 1.380(5), C(7)−C(8) 1.384(5), C(8)−C(9) 1.391(5), C(9)−N(1) 1.417(3). **Selected angles** (**^o^):** Zn(1)−O(01)−Zn(2) 110.5(1), O(01)−Zn(1)−O(1) 99.7(1), O(01)−Zn(2)−O(2) 106.3(1), O(01)−Zn(2)−O(2’) 92.0(1). **Symmetry code:** x, y, z


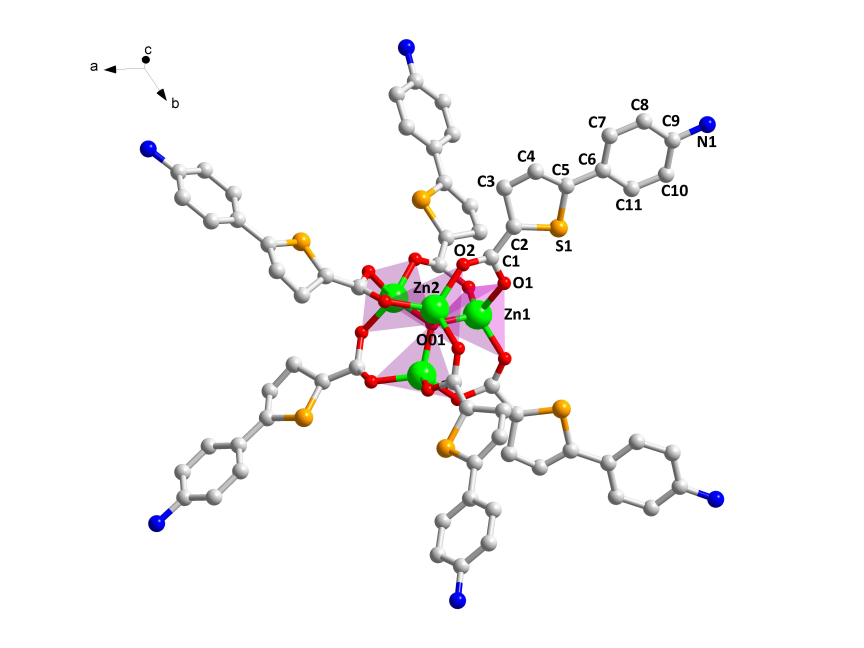


Supplementary Figure 4. The ball-and-stick diagram of Zn−TCTA showing the heavily distorted metal node. The hydrogen and the disordered atoms were omitted for clarity.


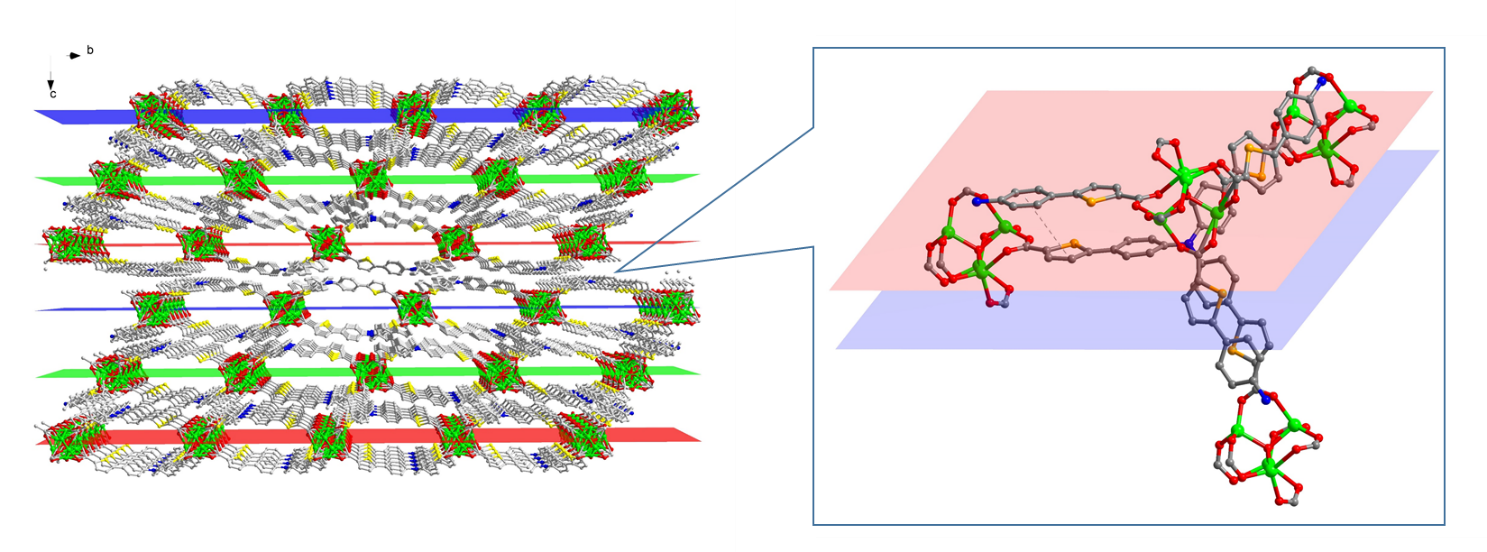


**(a)**

**(b)**

Supplementary Figure 5. The interlayer stacking mode of catalyst. (a) The ABCABC π···π stacking, and (b) the typical J-aggregate mode in a head-to-tail fashion between the 4-thienylphenyl moieties of TCTA ligands of adjacent layers of Zn−TCTA. The neighbouring layers are shown in blue, green, and red, respectively. The hydrogen and the disordered atoms were omitted for clarity. Selected interaction distances (Å): phenyl centroid···thiophenyl centroid 4.02, π···π parallel interplanar distance 3.54. Selected angles (^o^): π···π slippage angle 28.2.


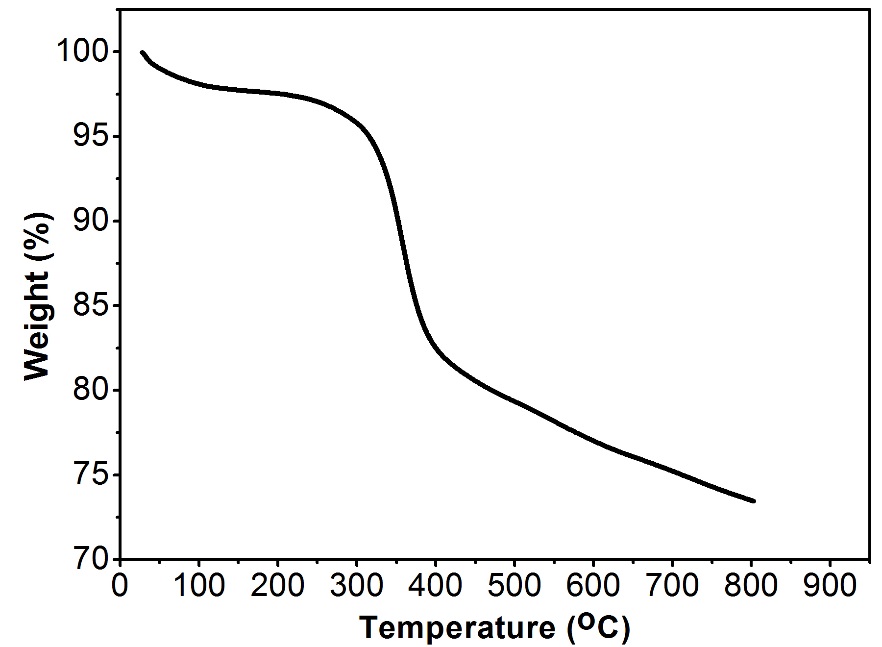


Supplementary Figure 6. Thermogravimetric figure of Zn−TCTA. The skeleton of Zn−TCTA is stable before 350 ^o^C. The first weight loss of *ca.* 3 % between 28 and 110 ^o^C is attributed to the loss of atmospheric water absorbed on the surface of Zn−TCTA crystals. Thermogravimetric analysis (TGA) was carried out at a ramp rate of 10 °C min^-1^ in a nitrogen flow.


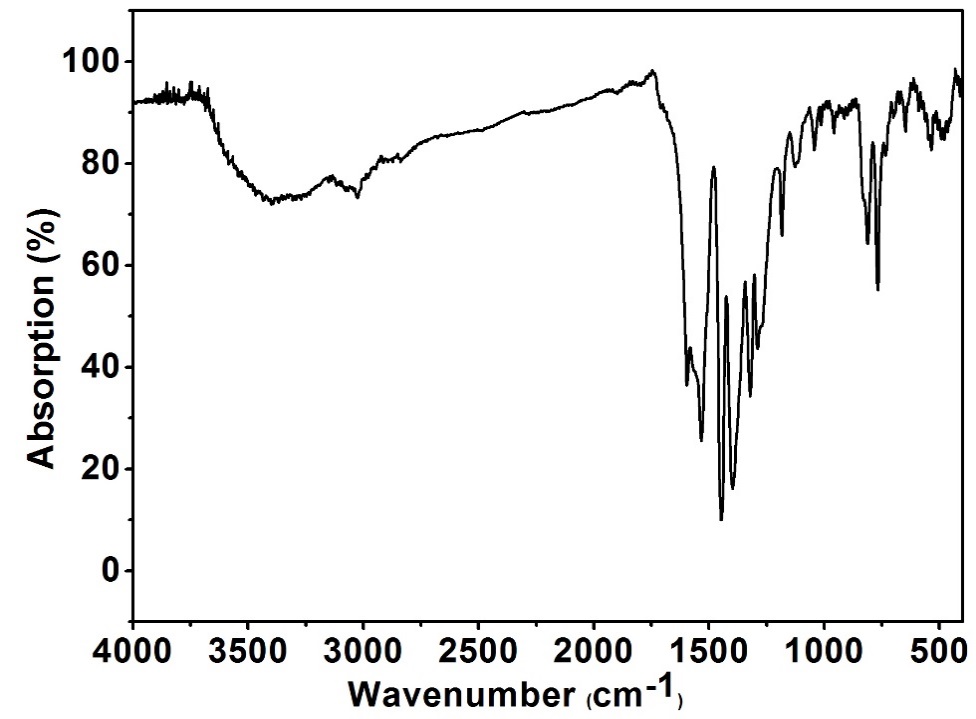


Supplementary Figure 7. FTIR spectra of Zn−TCTA.


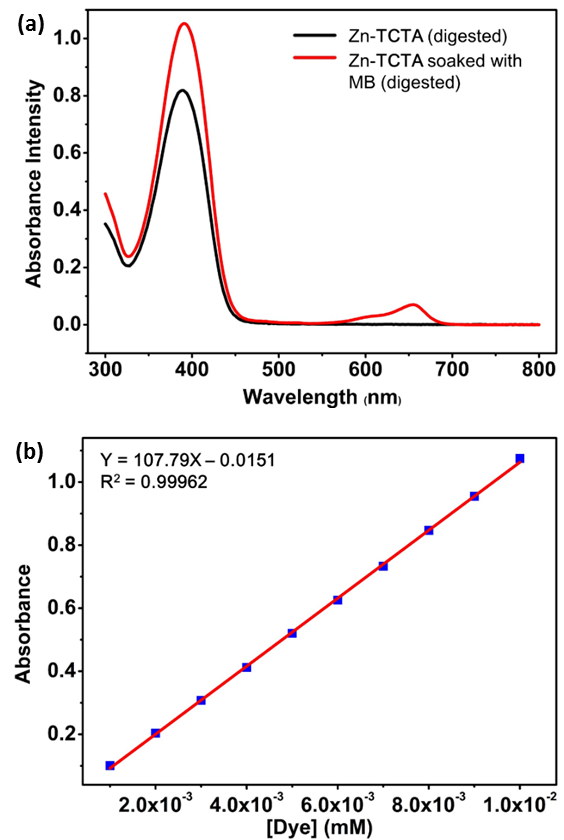


Supplementary Figure 8. Characterization of dye uptake amount of coordination polymer. (a) UV-Vis measurements of methylene blue dye released from Zn−TCTA. (b) The standard linear relationship between the absorption and the concentration.


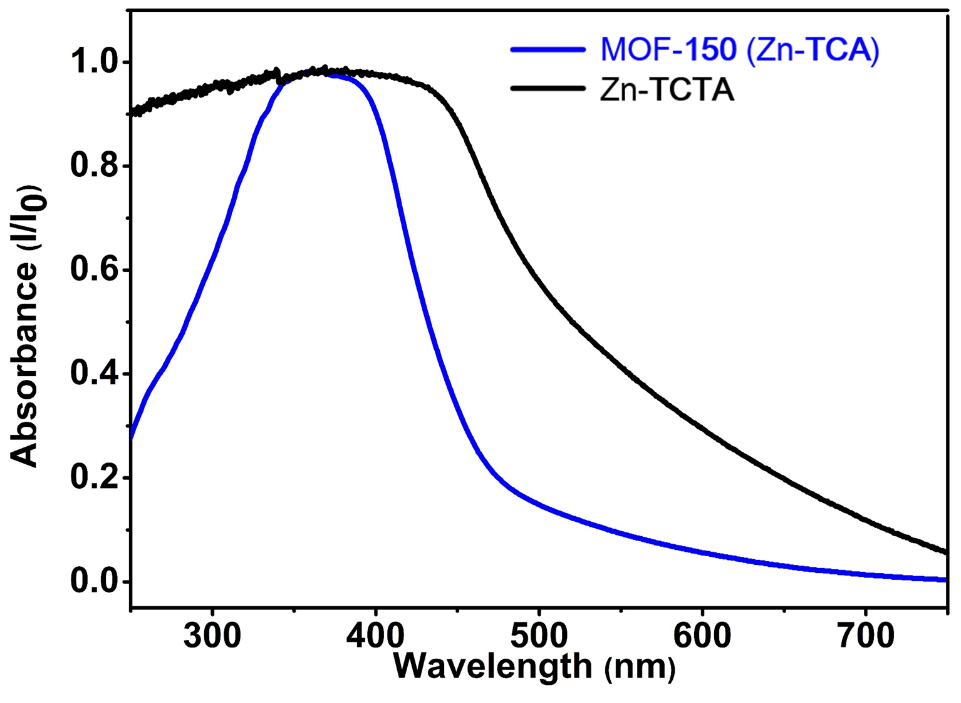


Supplementary Figure 9. Comparison of solid state UV-Vis absorption spectra of MOF−150 (blue line) and Zn−TCTA (black line), respectively.


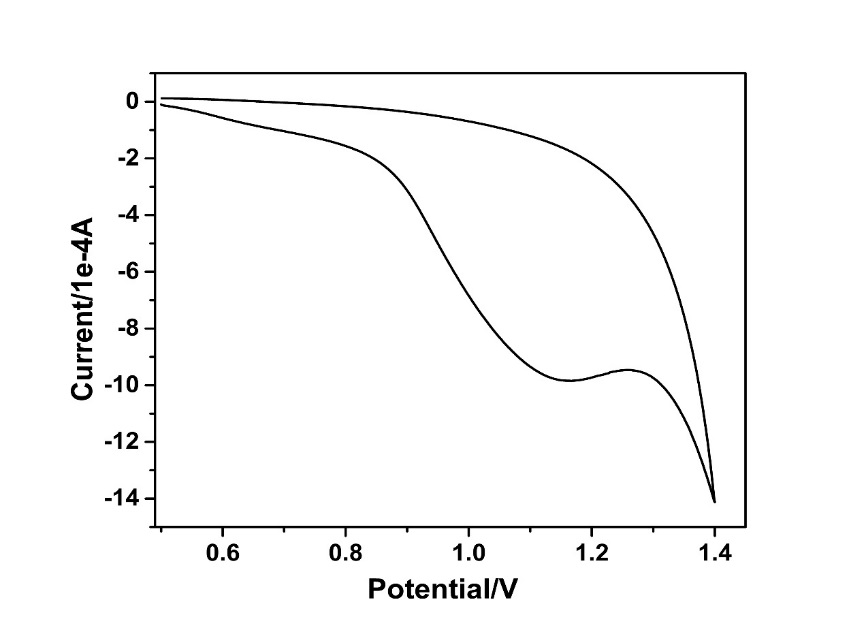


Supplementary Figure 10. Solid-state CV of Zn−TCTA. The Measurement was performed by using a three-electrode system in an aqueous solution of KNO_3_ (0.1 M) at a scan rate of 100 mV s^−1^, in the range 0.5−1.4 V.


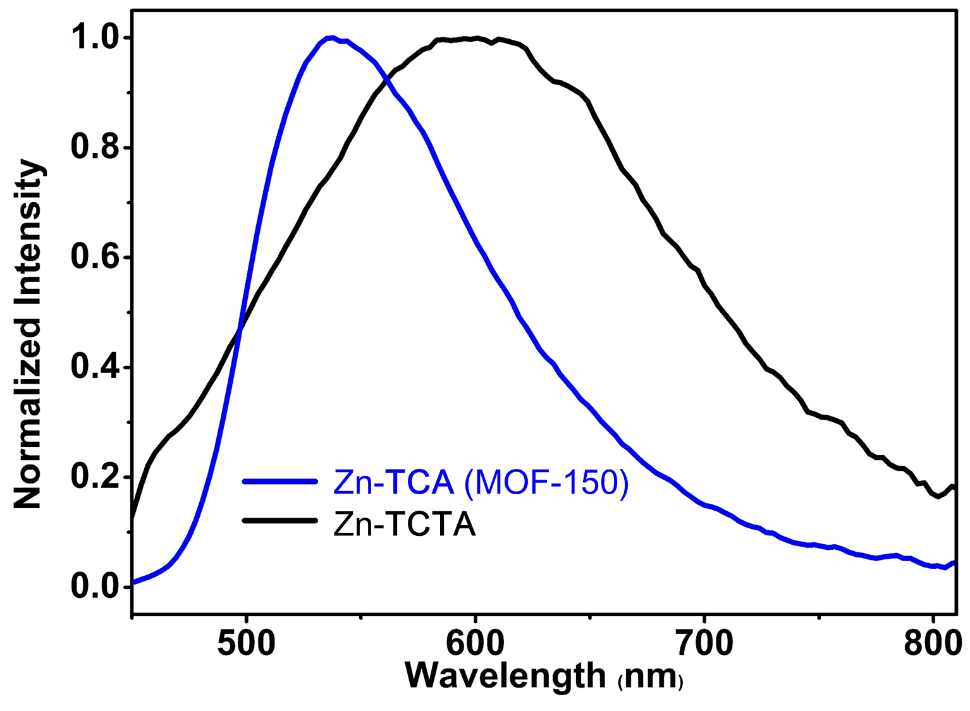


Supplementary Figure 11. Comparison of solid state emission spectra of MOF−150 (blue line) and Zn−TCTA (black line), respectively.


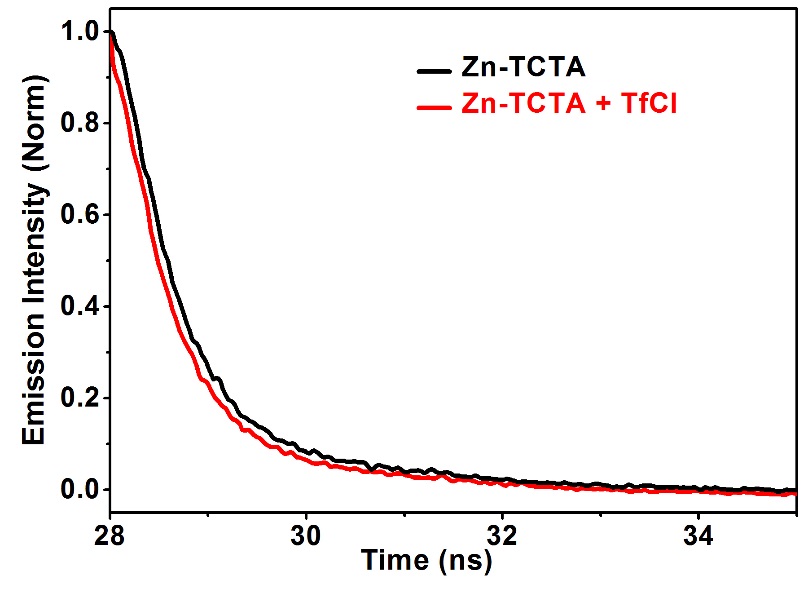


Supplementary Figure 12. Transient emission spectra of Zn−TCTA well suspended in acetonitrile (black line; *τ* = 2.12 ns) and the one upon addition of TfCl (red line; *τ* = 1.57 ns). The intensities were recorded at 546 nm, on excitation at 390 nm.


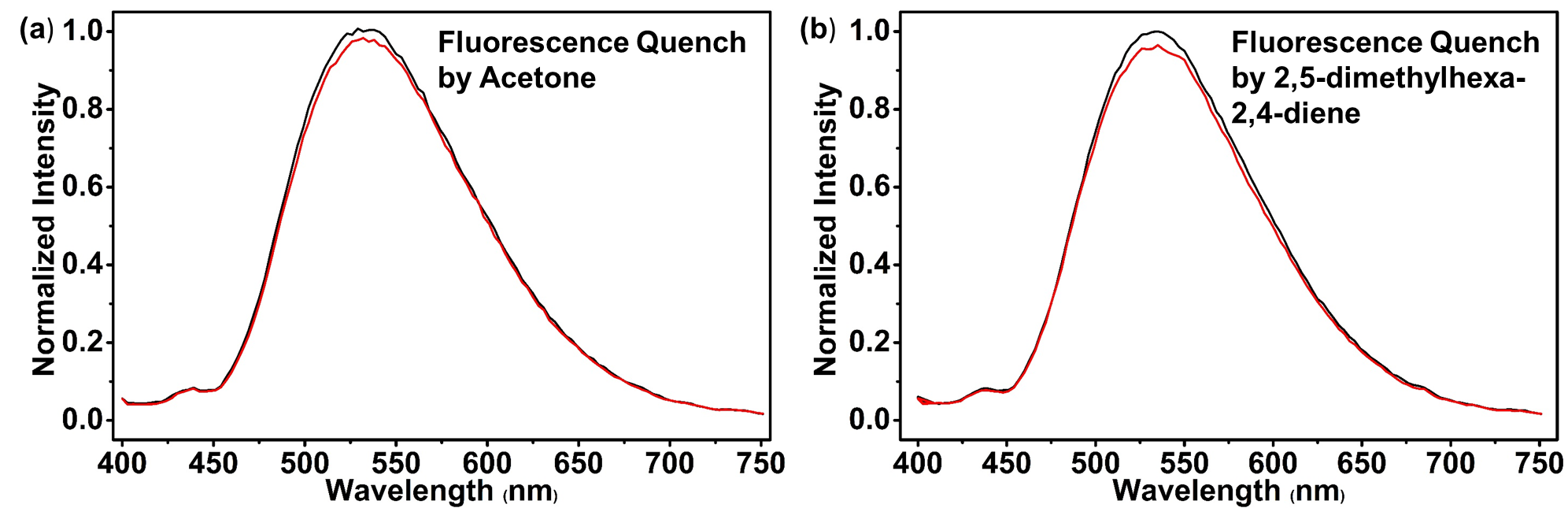


Supplementary Figure 13. Comparison of fluorescence spectra of Zn–TCTA well suspended in acetonitrile (black line) and the one (red line) upon the addition of triplet quencher (a) acetone (0.25 mmol, the same amount as the substrate 1a used in Table 1, 2a) or (b) 2,5-dimethylhexa-2,4-diene (0.25 mmol). The excitation was at 390 nm, and the intensity was recorded at 546 nm.


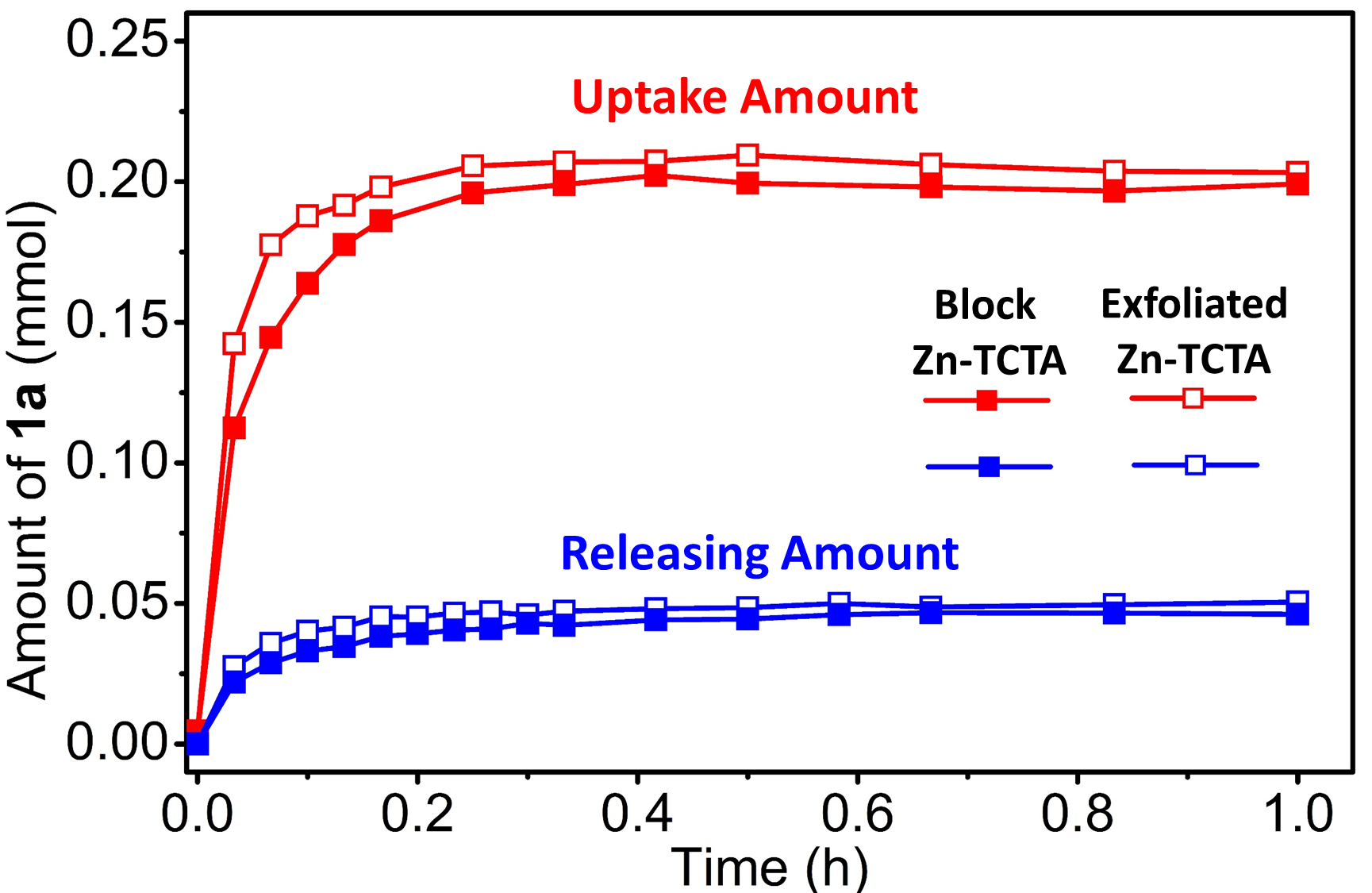


Supplementary Figure 14. Comparison of substrate 1a ingress and egress by block Zn−TCTA and the recovered exfoliated sample after reaction, respectively.


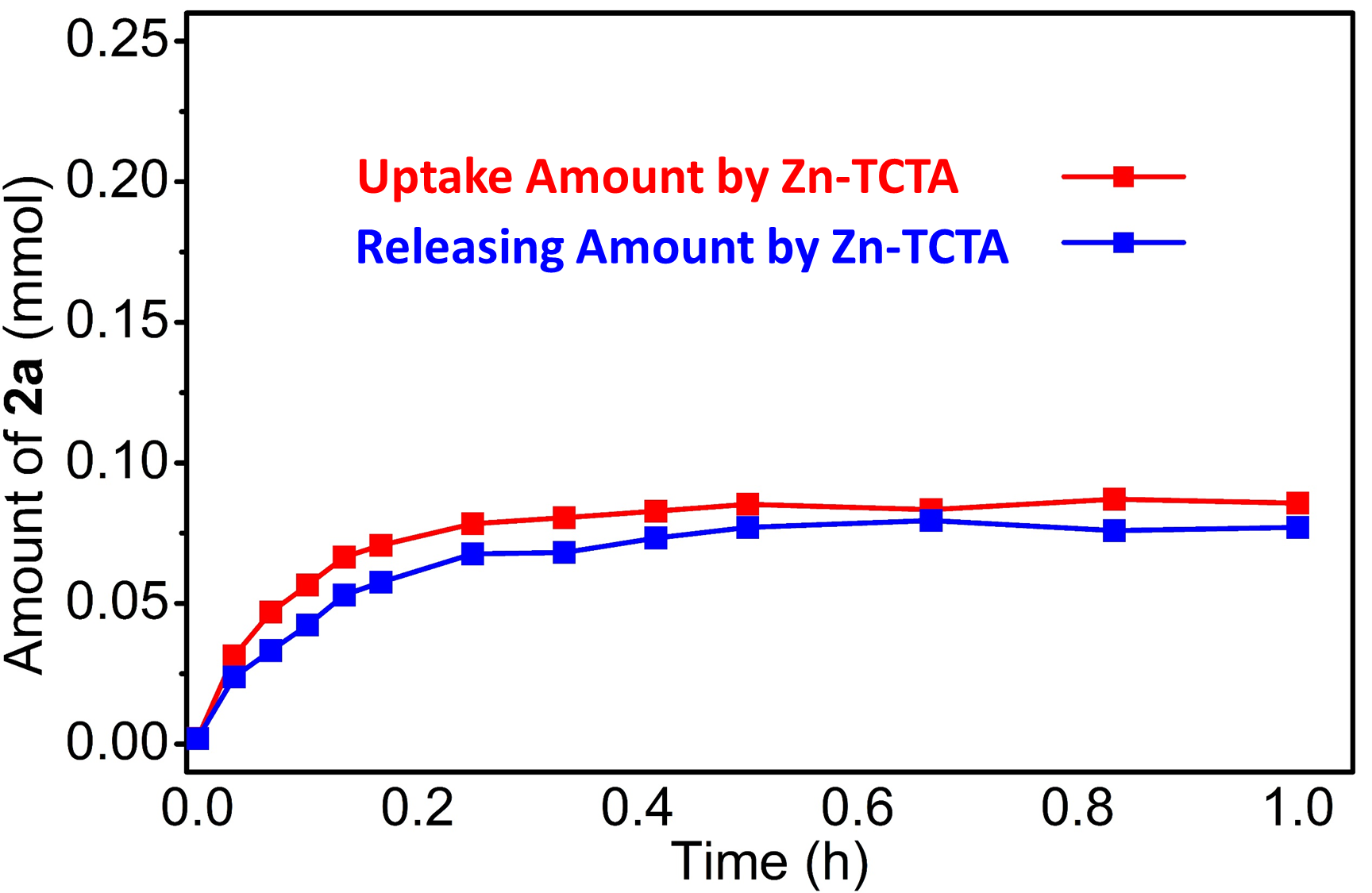


Supplementary Figure 15. Ingress and egress of target product 2a by block crystal Zn−TCTA.


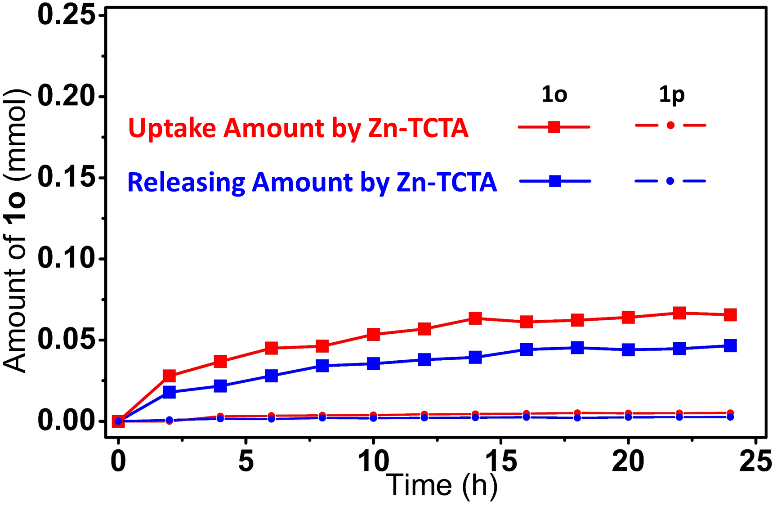


Supplementary Figure 16. Comparison of ingress (red line) and egress (blue line) of substrates 1o (square) and 1p (dot) on block crystal Zn−TCTA, respectively. Owing to the high boiling points of 1o and 1p, the uptake and release amounts were monitored by ^1^H NMR and the ingress/egress experiments were conducted in CD_3_CN instead of CH_3_CN.


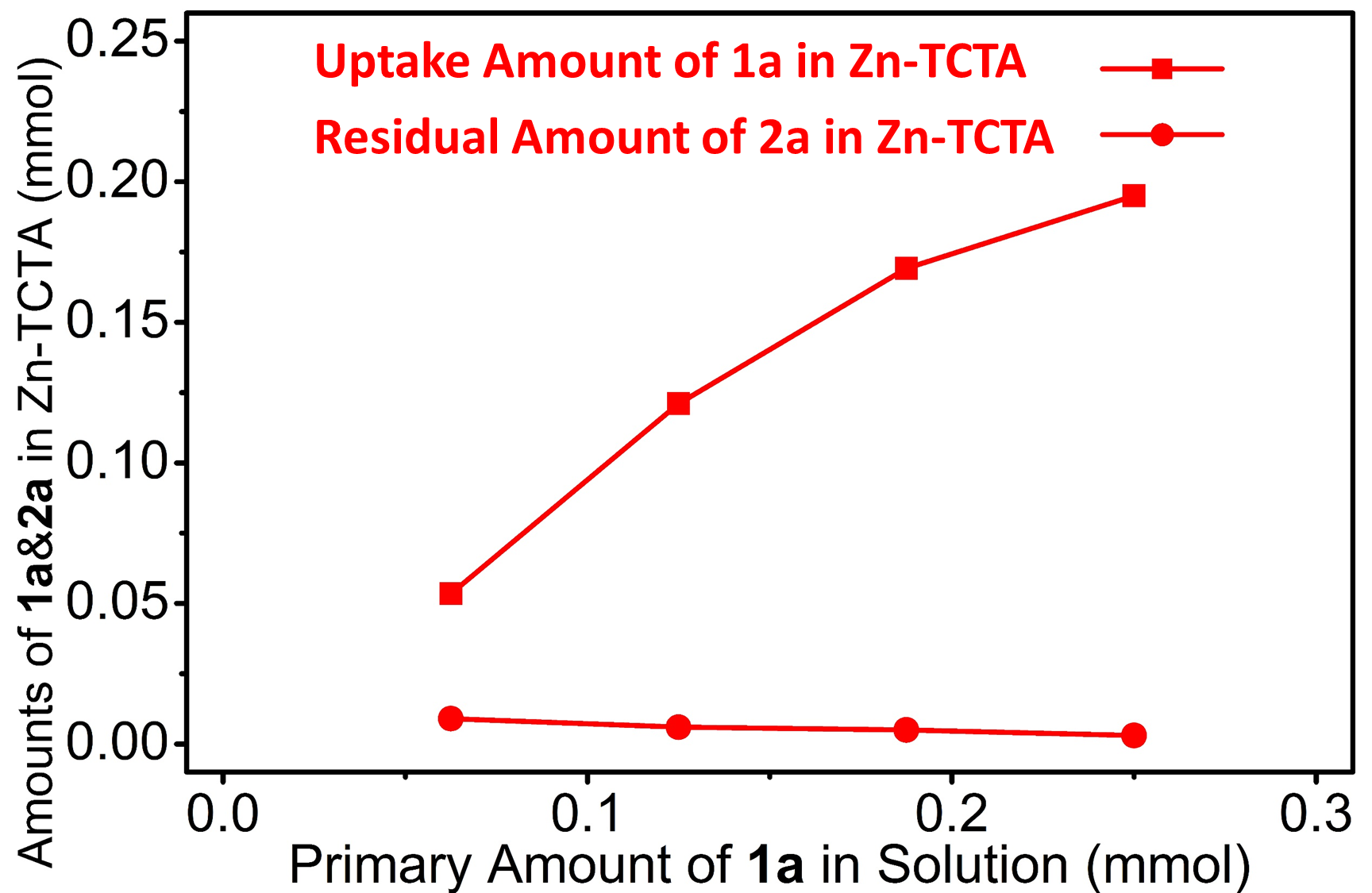


Supplementary Figure 17. Competitive sorption of substrate 1a and product 2a within Zn−TCTA in the presence of different primary amount of substrate 1a.


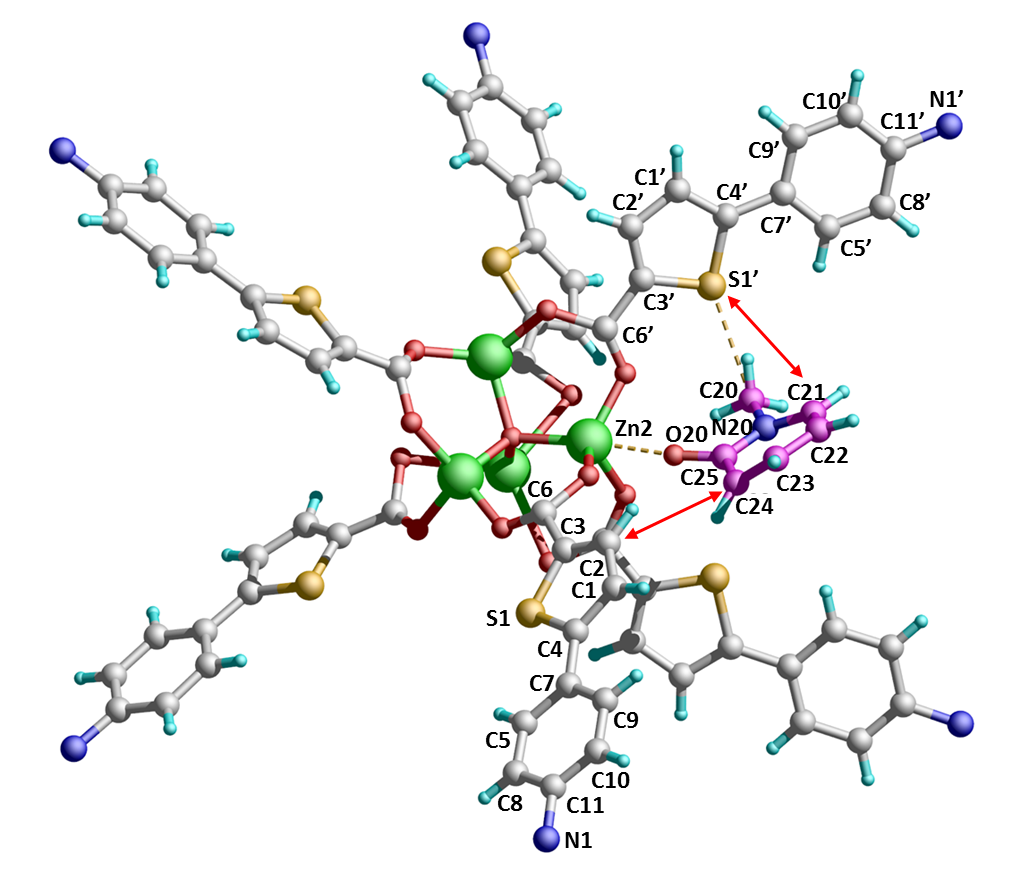


Supplementary Figure 18. Framework of 1a@Zn−TCTA in a unit with selected labelling scheme, showing the coordination interactions between the unprotected zinc atom and the carbonyl oxygen of 1a, and the C−H···S interaction between the thiophene sulfur and *N*-methyl of 1a. The heavily disordered guest molecules were only shown in one set for clarity (occupation of atoms of 1a = 1/3). Selected bond/interaction distances (Å): Zn(2)···O(20) 2.20, S(1′)···C(20) 2.87. Selected NON-bond distances (Å): the nearest distance between 1a and TCTA moiety C(2)···C(24) 3.75 < S(1′)···C(21) 4.11. Selected angles (^o^): C(25)−O(20)···Zn(2) 169.6, N(20)−C(20)···S(1′) 111.3. Symmetry codes: -x+y, -x, z; ′ -y, x-y, z.


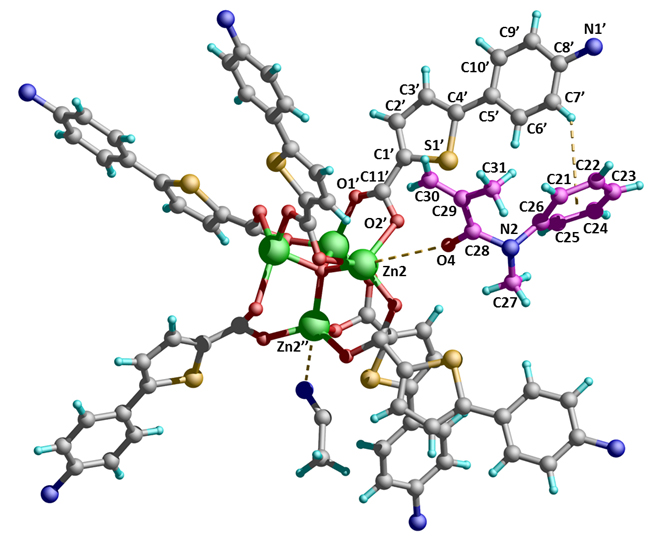


Supplementary Figure 19. Framework of 3a@Zn−TCTA in a unit with selected labelling scheme, showing the weak coordination effect between unprotected zinc atom and carbonyl oxygen of 3a, and the C−H···π interaction between phenyl moiety of TCTA ligand and phenyl moiety of 3a. The heavily disordered guest molecules were only shown in one set for clarity (occupation of atoms of 3a = 1/3). Selected bond/interaction distances (Å): Zn(2)···O(4) 3.76, C(7′)···π centroid 4.06, C(7′)···π plane 3.95, C(7′)···C(23) 4.07 (nearest inter-carbon atomic distance of C−H···π). Selected angles (^o^): C(28)−O(4)···Zn(2) 164.5, C(7′)−H(7′)···π centroid 138.9. Symmetry codes: -y, x-y, z; ′ -x+1/3, -x+y+2/3, -z+1/6, ′′ x, y, z.


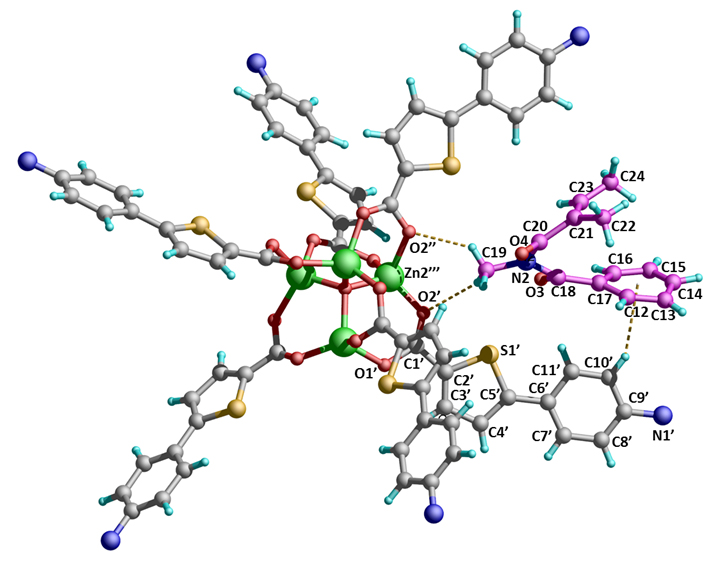


Supplementary Figure 20. Illustration of non-covalent interactions between encapsulated 3f in a unit of Zn−TCTA (upper). The heavily disordered guest molecules were only shown in one set for clarity (occupation of atoms of 3f = 1/3). Selected interaction distances (Å): O(2′)···C(19) 3.46, O(2′′)···C(19) 3.35, C(10′)···π centroid 3.74, C(10′)···π plane 3.66, C(10′)···C(17) 3.80 (nearest inter-carbon atomic distance of C−H···π). Selected angles (^o^): average of C(19)−H(19A)···O(2′) and C(19)−H(19B)···O(2′′) 135.9, C(10′)−H(10′)···π centroid 148.5. Symmetry codes: y+1/3, x+2/3, -z+1/6; ′ x-y+1/3, -y+2/3, -z+1/6, ′′ x, y, z; ′′′ -x+y, -x, z.


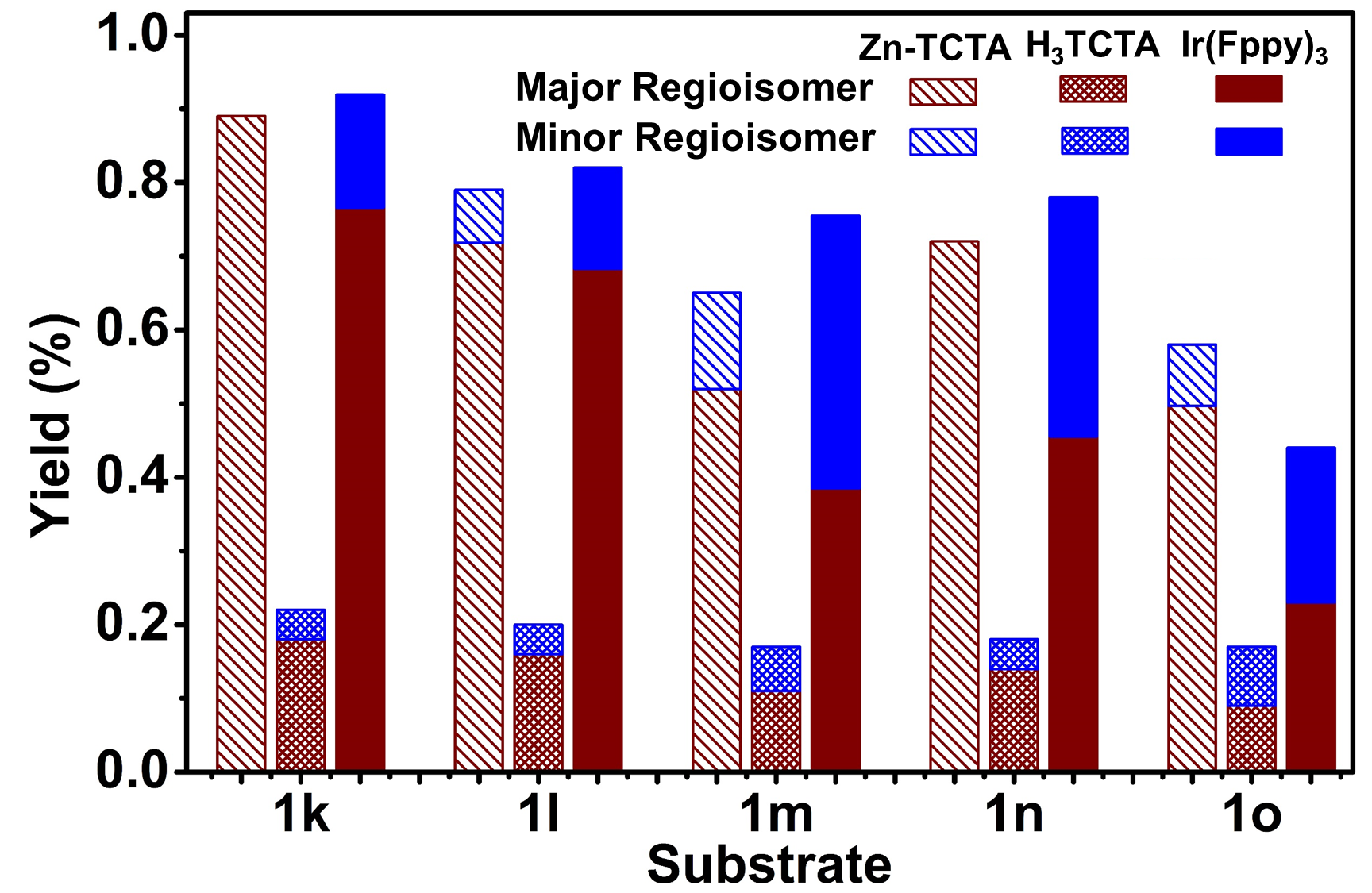


Supplementary Figure 21. Comparison of histograms of regioselectivities and of using Zn−TCTA, H_3_ TCTA, and *fac*-Ir(Fppy)_3_, respectively.


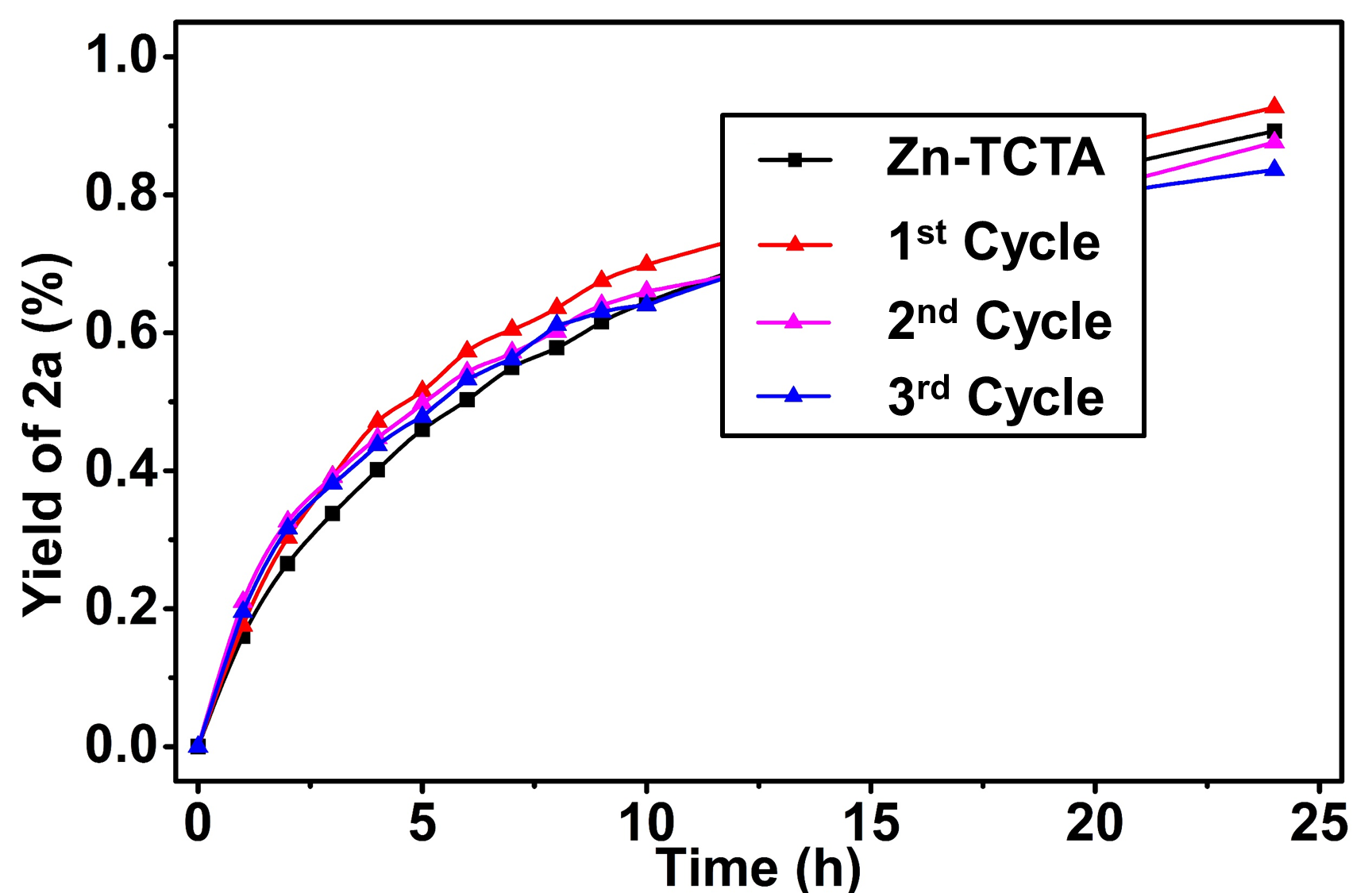


Supplementary Figure 22. Comparison of time-conversion plots of the trifluoromethylation of 1a using fresh Zn–TCTA and the recycled ones.


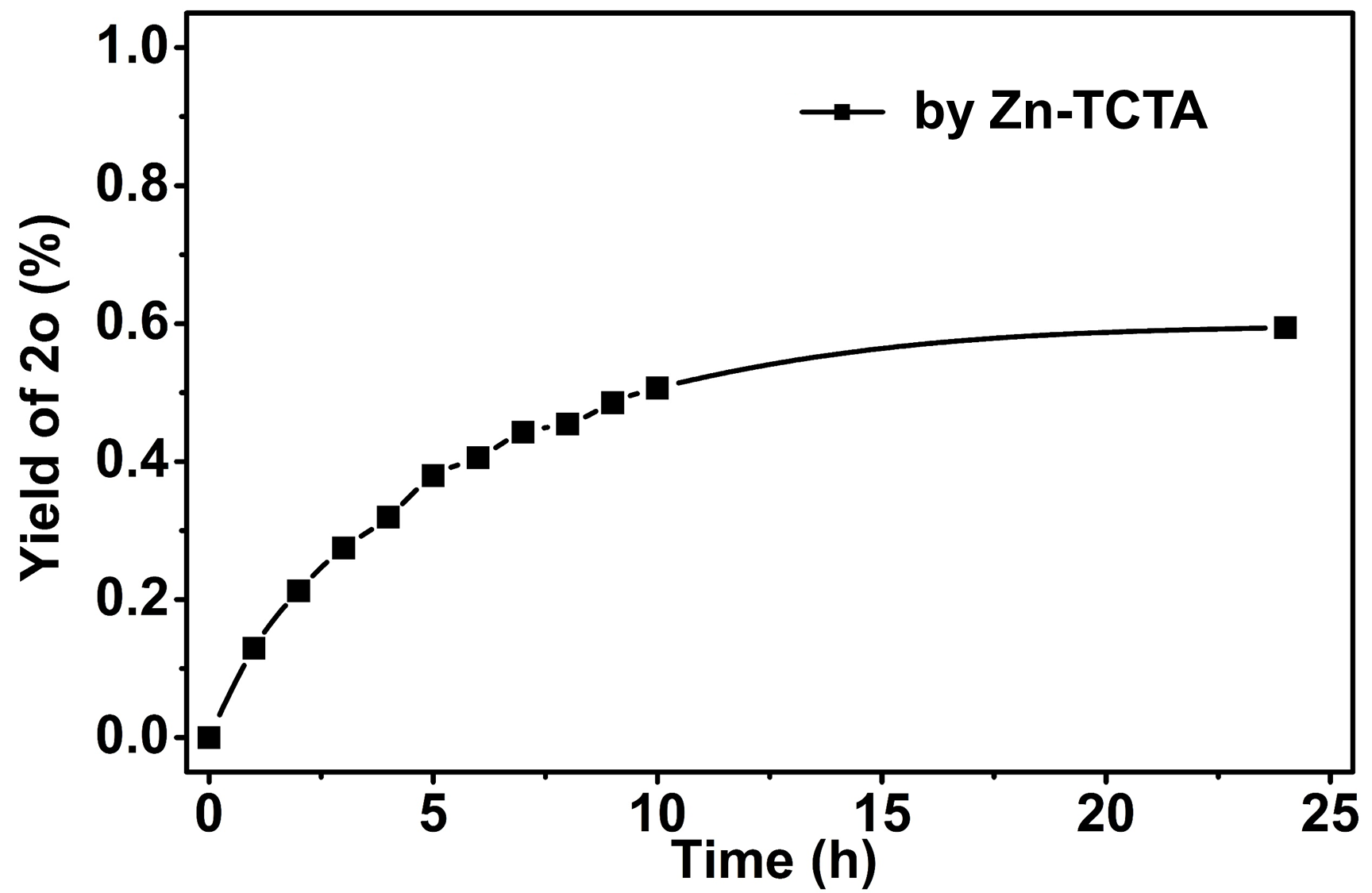


Supplementary Figure 23. The time-conversion plots of the trifluoromethylation of 1o in the presence of Zn–TCTA.


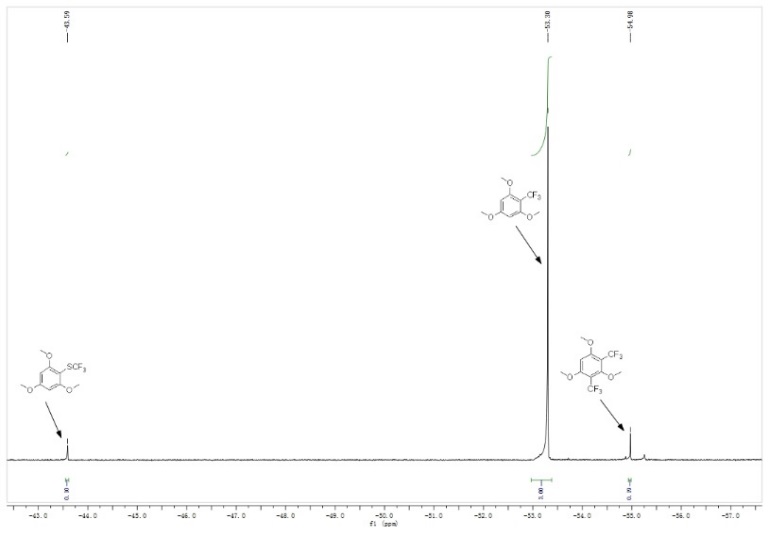

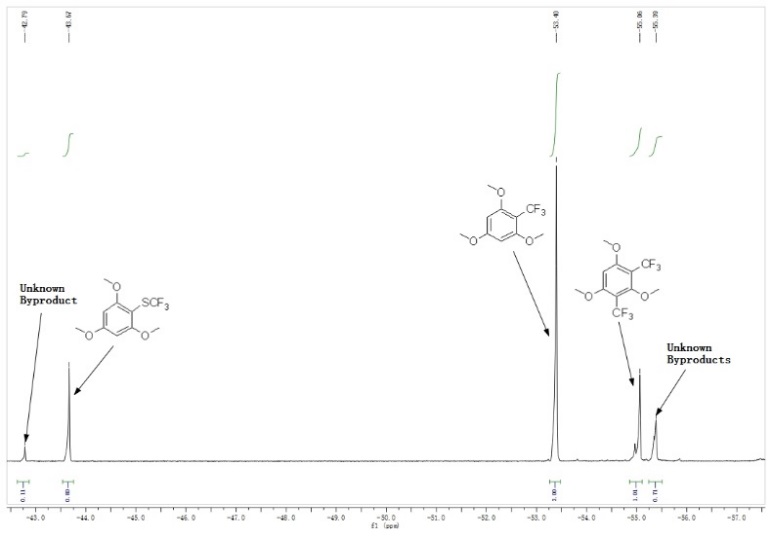


**(b)**

**(a)**

Supplementary Figure 24. Comparison of crude ^19^F NMR spectra (in CD_3_CN) of photoreactions of 1j catalysed by Zn−TCTA (a) and MOF−150 (b), respectively. The peaks of electrophilic byproduct (trifluoromethyl)(2,4,6-trimethoxyphenyl)sulfane^16^ and the radical type over-trifluoromethylated byproduct 1,3,5-trimethoxy-2,4-bis(trifluoromethyl)benzene were assigned according to literatures^10^. It is postulated that the *in situ* exfoliation of Zn–TCTA helped to improve the mass transfer ability and light transmittance of the photocatalyst, increasing the photocatalytic efficiency and suppressing over-trifluoromethylation and other non-photoirradiative side reactions, especially when electron-rich arenes were employed.


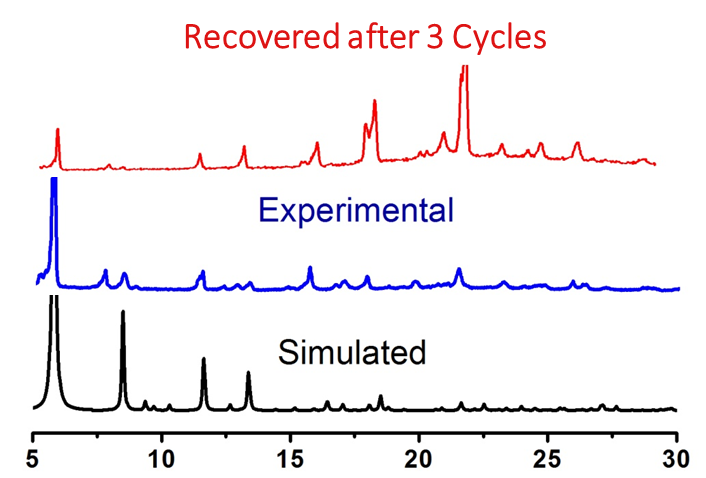


Supplementary Figure 25. Simulated (black) and experimental (blue) XRD patterns of the Zn–TCTA crystal and the experimental pattern of the catalyst recovered after 3 cycles of catalysis (red).


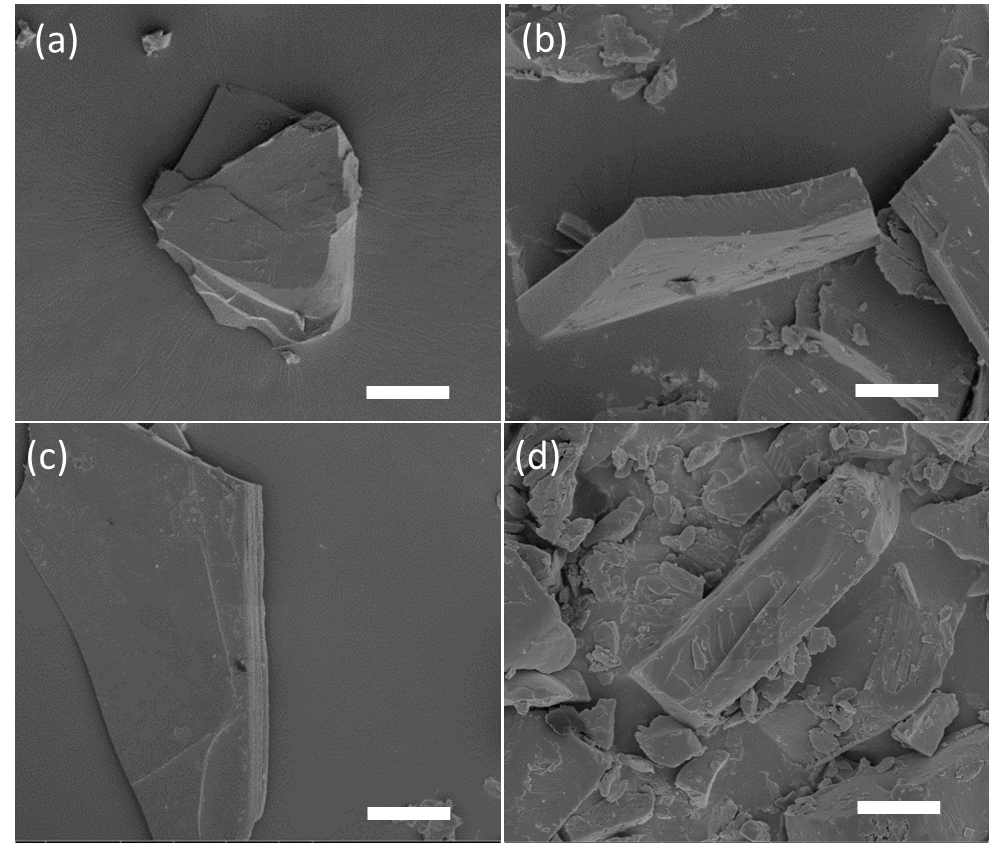


Supplementary Figure 26. SEM images of catalyst. The fresh Zn−TCTA crystals with size of *ca.* 100~300 μm (a). The less effective *in situ* exfoliations of Zn−TCTA in the absence of light (b) or stirring (c), respectively. When stirring was absent, the photoreaction mixture was shaken by a vortex reactor instead. In comparison, when mortar was used, the Zn−TCTA block crystals could be grounded to particles with decreased size, but not effectively exfoliated to thin layers (d). Inset scale bars of a and c, 50 μm; for b and d, 4 μm. The exfoliation was postulated to be due to breaking of the interlayer π-π stacking by the comprehensive effects of mechanical shearing forces during stirring, weak interactions relevant to the solvents and other organic compounds, and interlayer charge repulsions during the photoredox cycles.


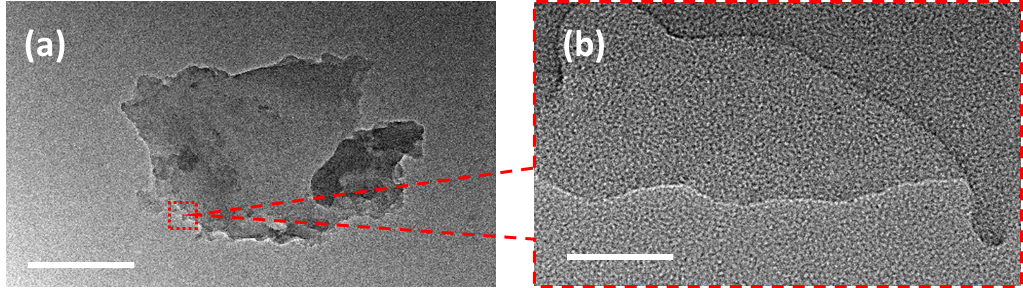


Supplementary Figure 27. TEM images of catalyst. Exfoliated thin layers of Zn−TCTA (inset, scale bar, 500 nm) (a) and a magnified image showing the step-like laminated cross sections (inset, scale bar, 20 nm) (b, also shown as Fig. 1f).


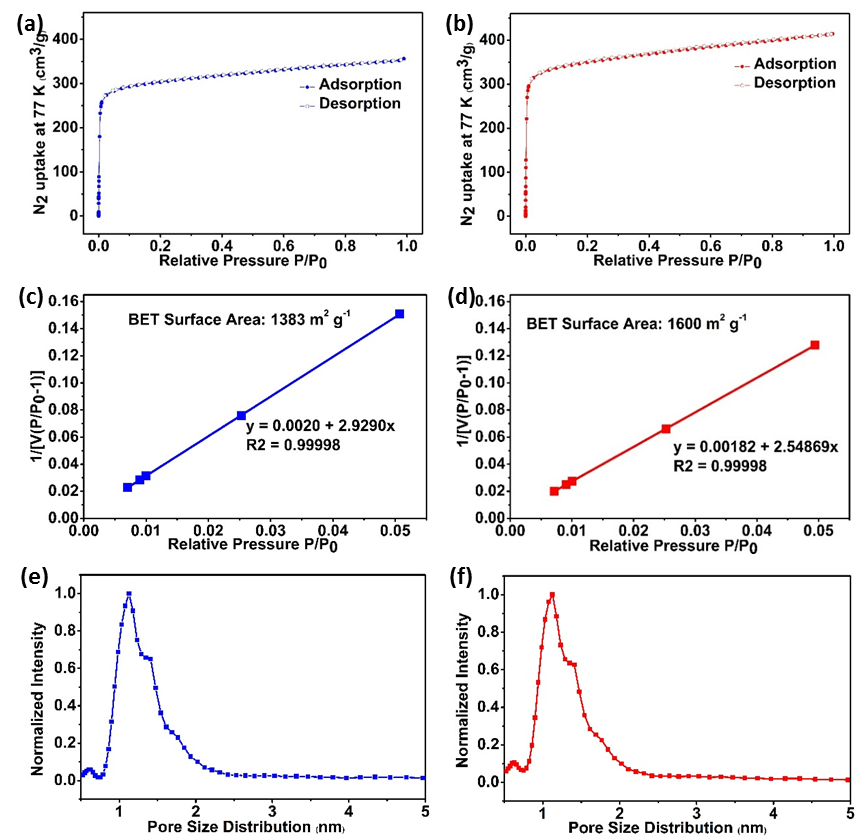


Supplementary Figure 28. Characterization and analyses on N_2_ adsorption/desorption of catalyst. (a and b) N_2_ adsorption/desorption isotherms (filled symbols: adsorption; open symbols: desorption) collected at 77K. (c and d) BET plots. (e and f) Pore size distributions determined by the DFT method from N_2_ sorption isotherms. Fresh Zn-TCTA crystals (blue; a, c, and e), the recovered catalyst after 3 cycles (red; b, d, and f).


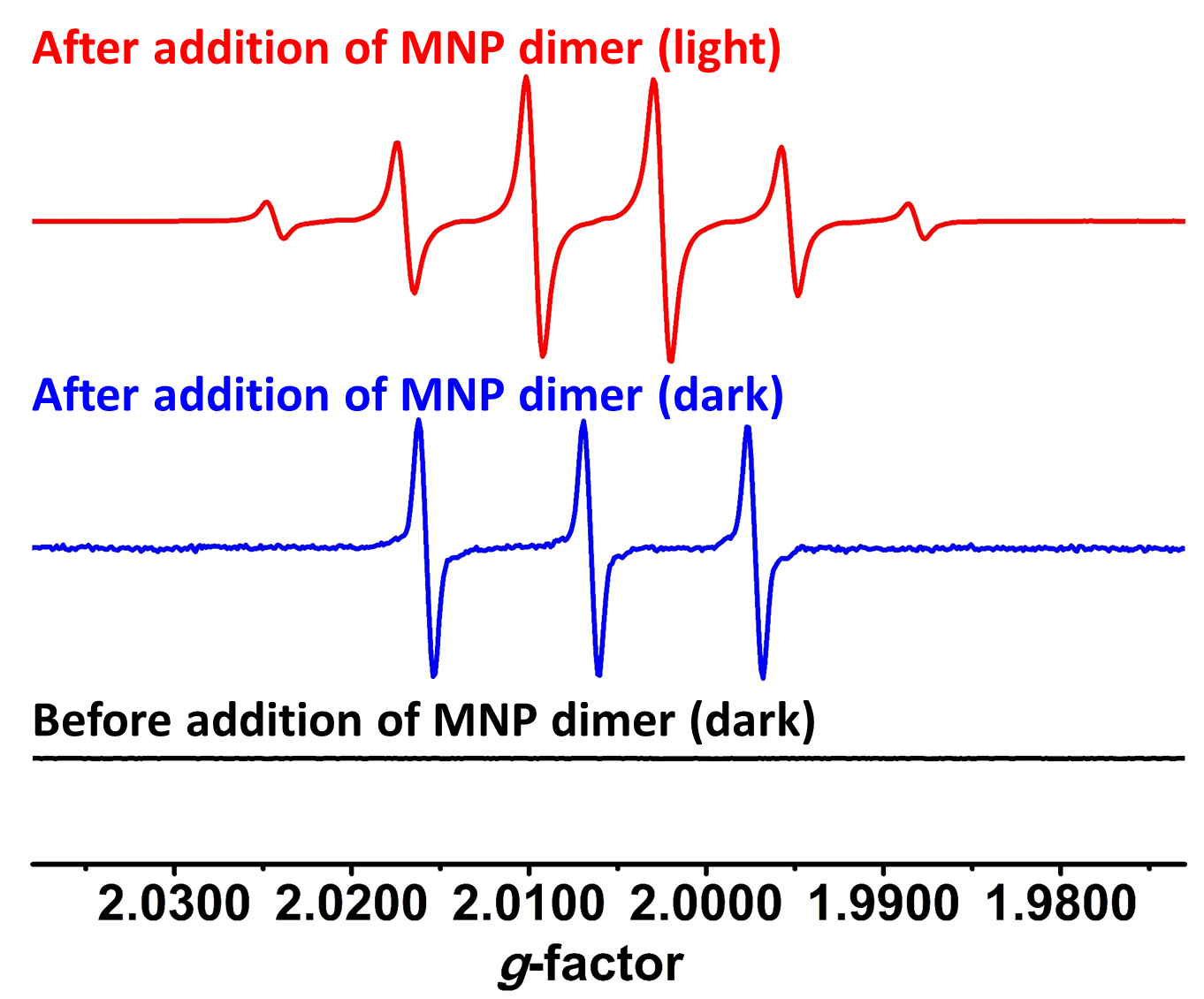


Supplementary Figure 29. EPR spectra of reaction mixture before (lower, black) and after (middle, blue) addition of a typical CF_3_ radical scavenger 2-methyl-2-nitrosopropane (MNP) dimer without visible light irradiation, and the one after visible light irradiation for 3 min (above, red).

NMR Spectrum of Substrates and Photocatalytic Products.


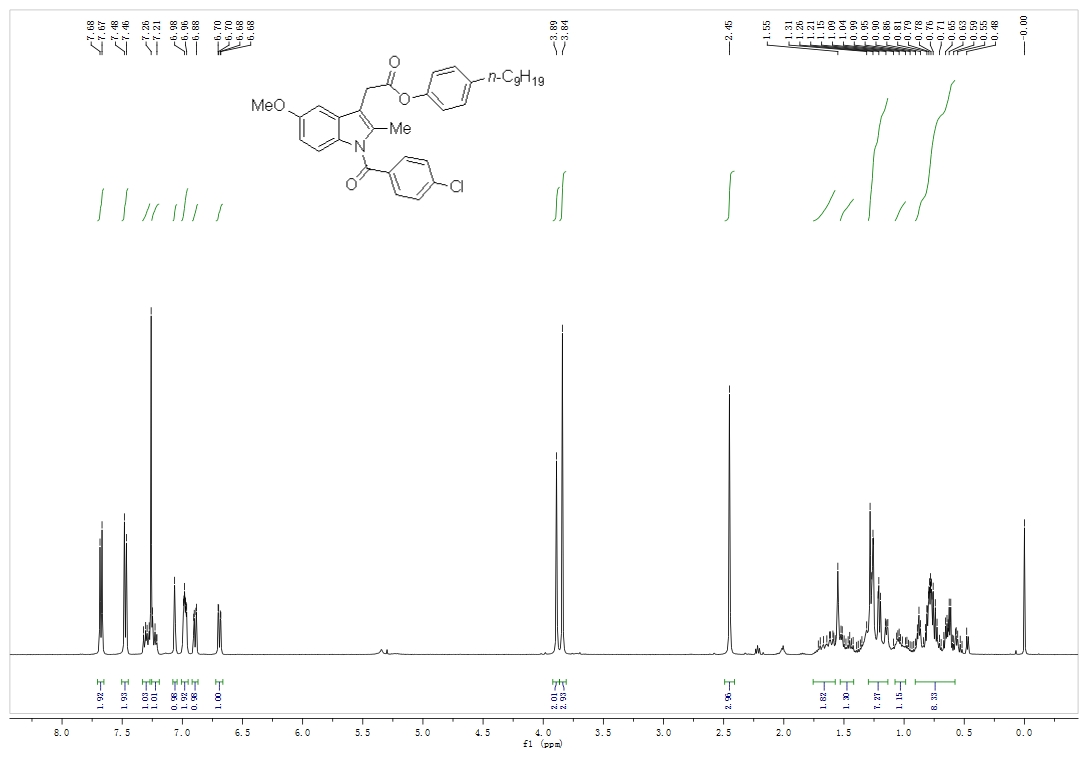


Supplementary Figure 30. ^1^H-NMR spectra of compound 1p.


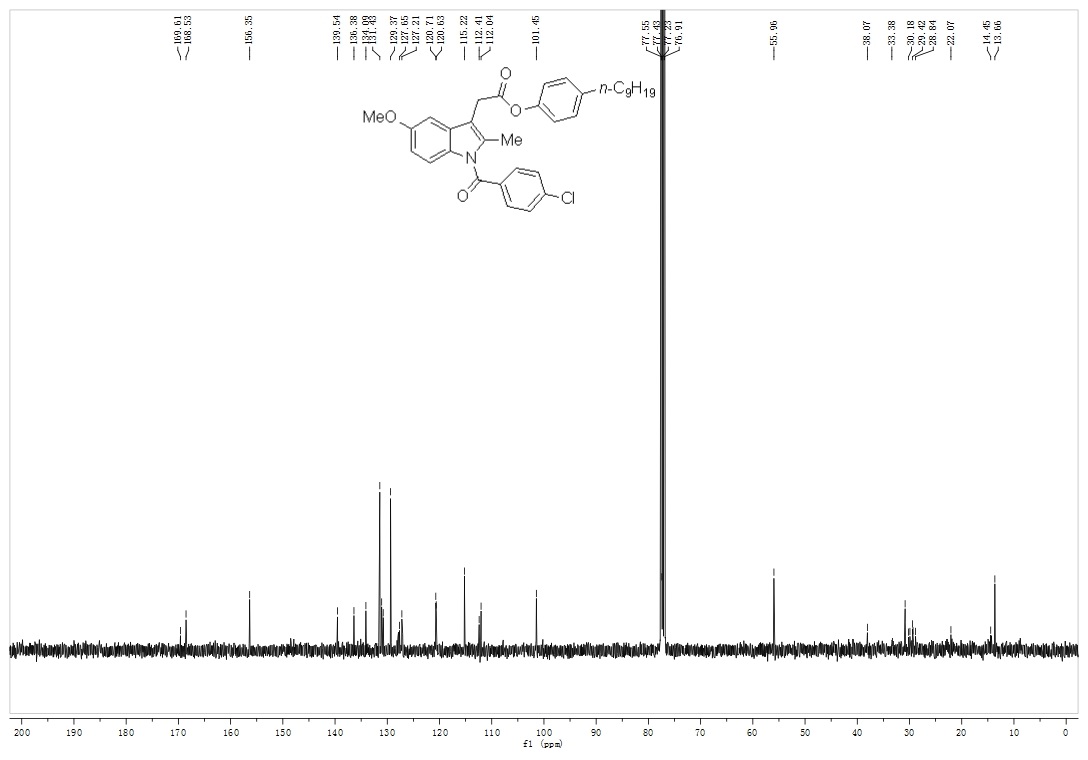


Supplementary Figure 31. ^13^C-NMR spectra of compound 1p.


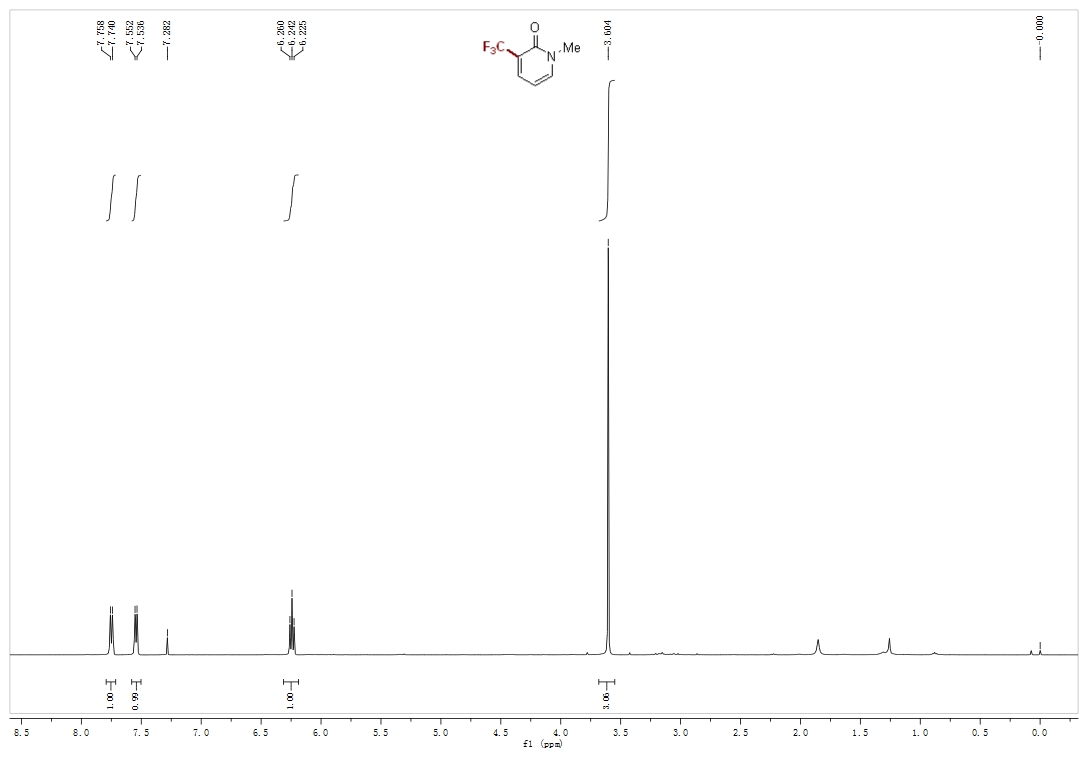


Supplementary Figure 32. ^1^H-NMR spectra of compound 2a.


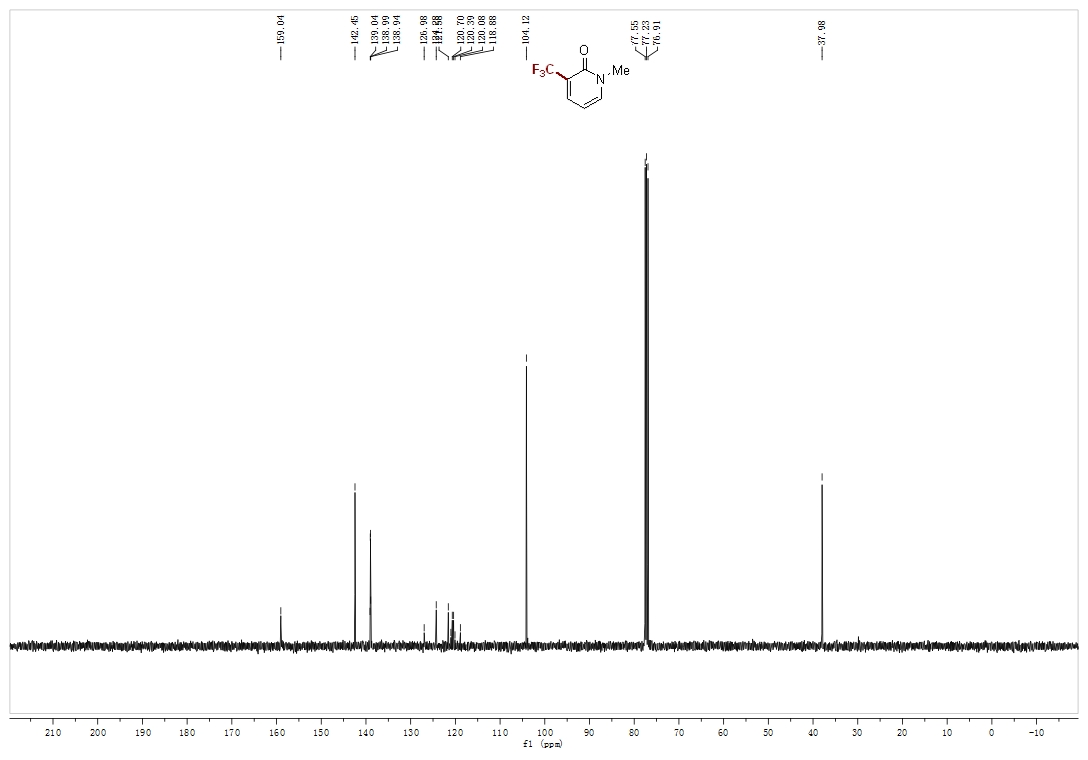


Supplementary Figure 33. ^13^C-NMR spectra of compound 2a.


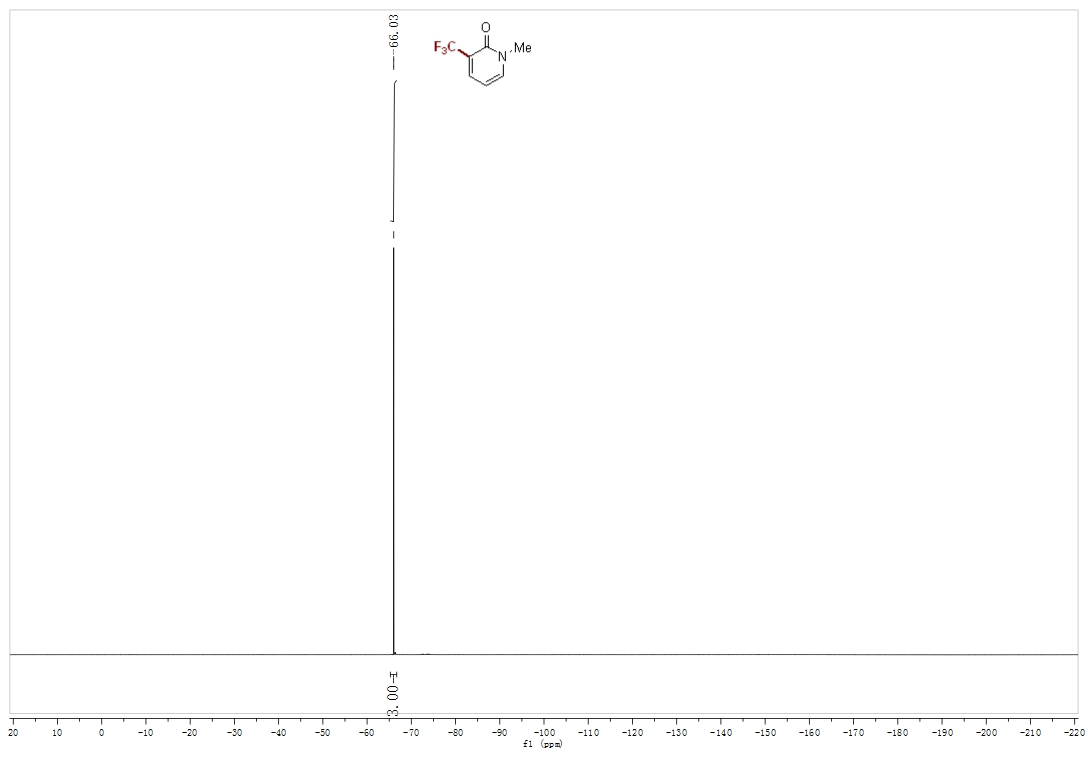


Supplementary Figure 34. ^19^F-NMR spectra of compound 2a.


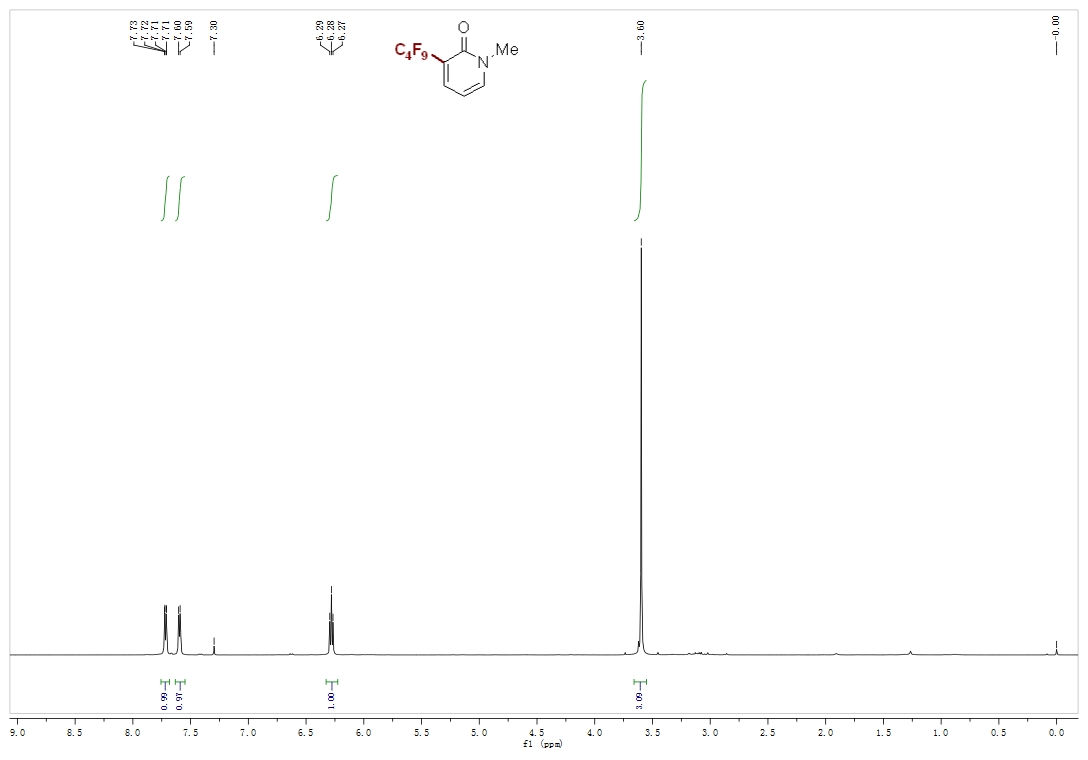


Supplementary Figure 35. ^1^H-NMR spectra of compound 2aa.


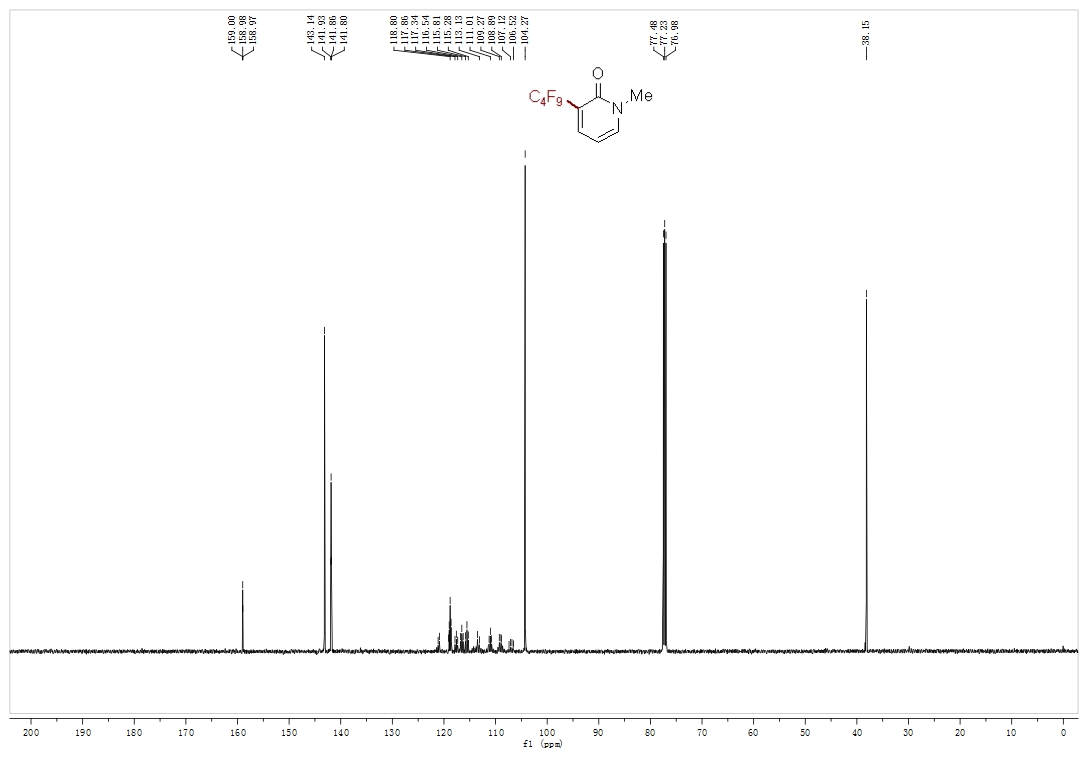


Supplementary Figure 36. ^13^C-NMR spectra of compound 2aa.


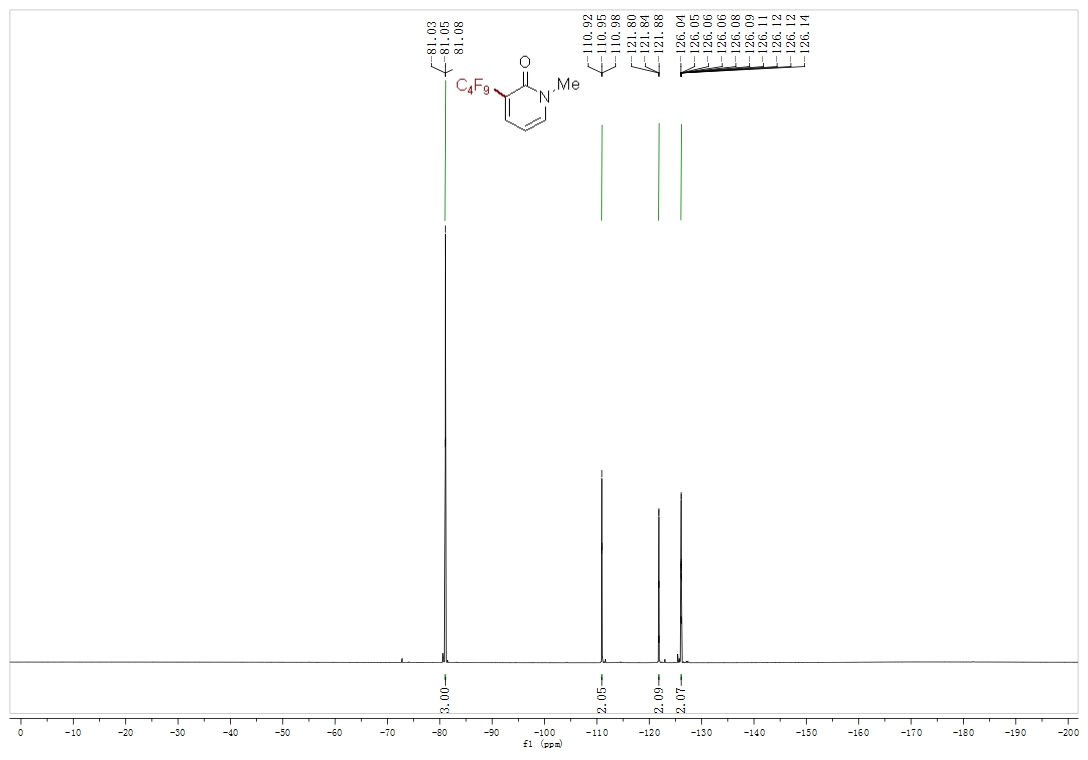


Supplementary Figure 37. ^19^F-NMR spectra of compound 2aa.


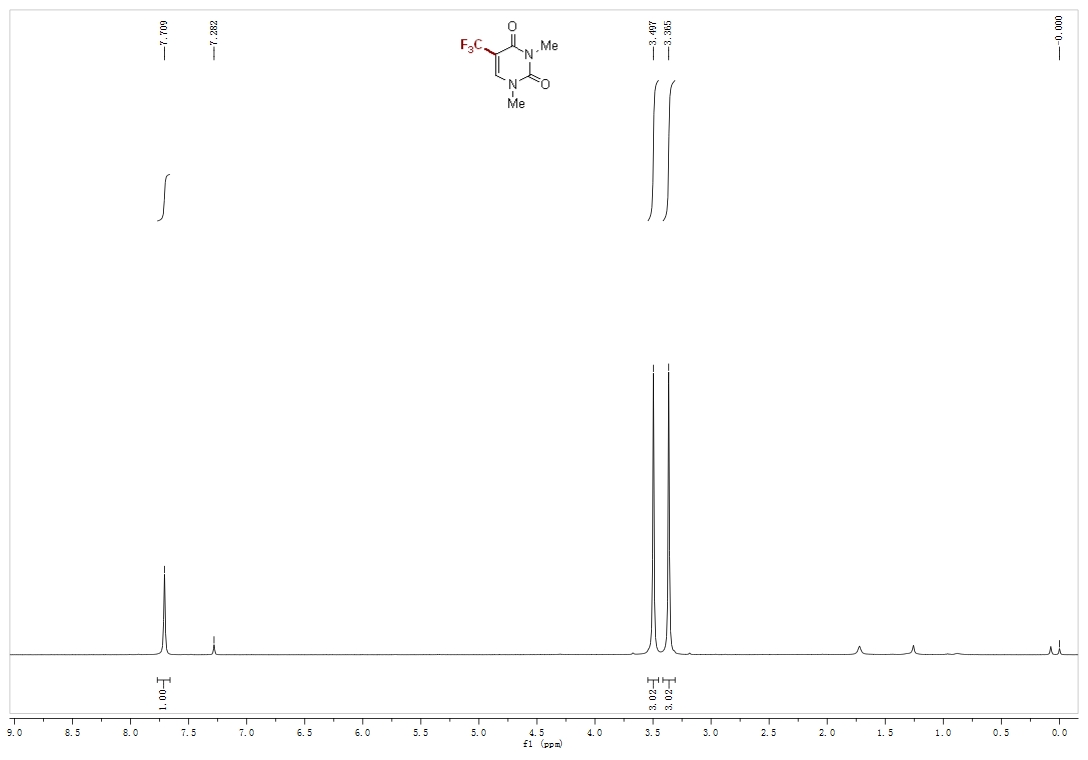


Supplementary Figure 38. ^1^H-NMR spectra of compound 2b.


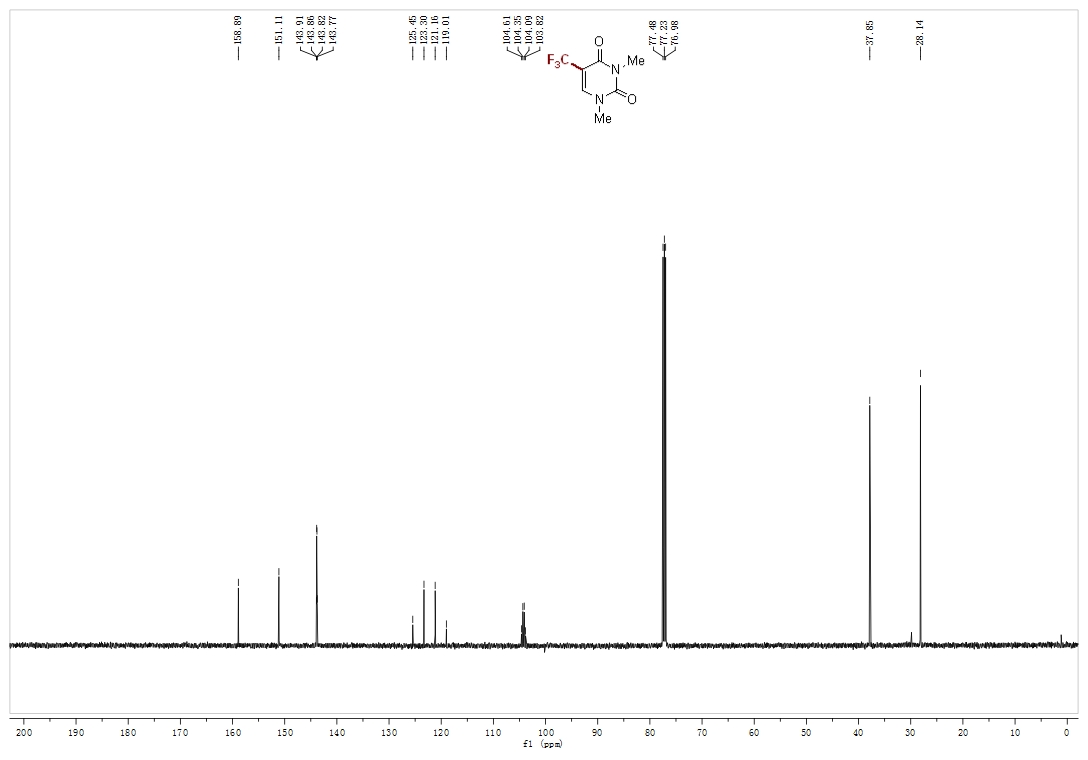


Supplementary Figure 39. ^13^C-NMR spectra of compound 2b.


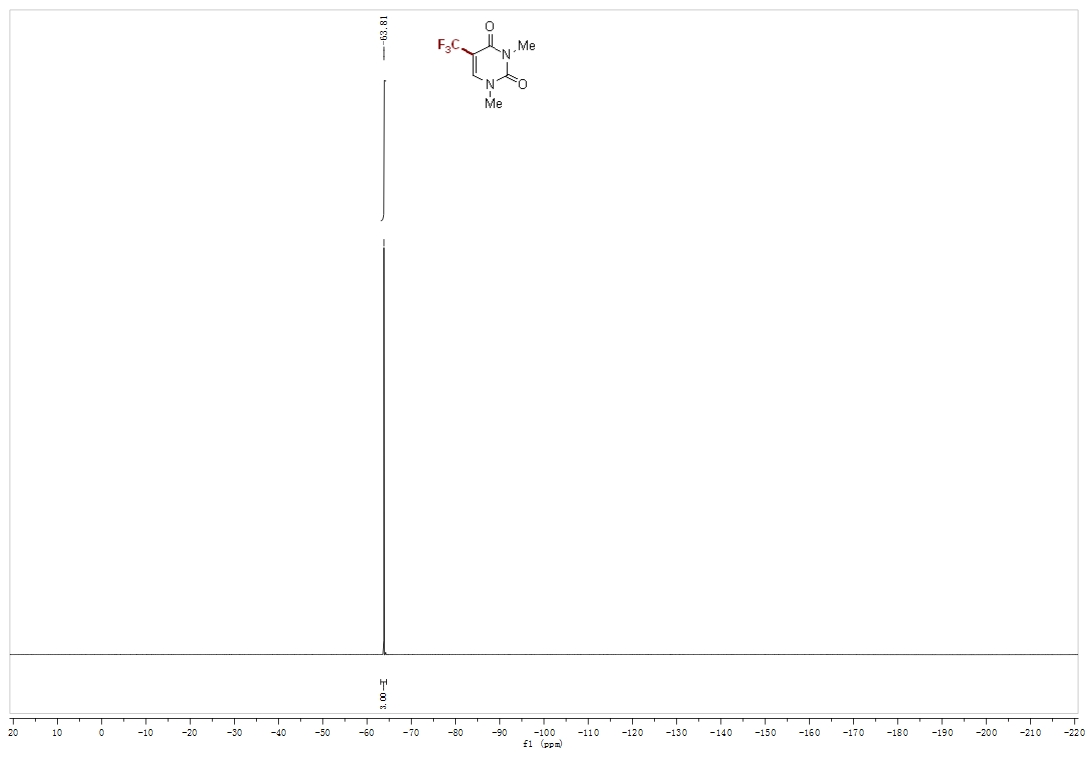


Supplementary Figure 40. ^19^F-NMR spectra of compound 2b.


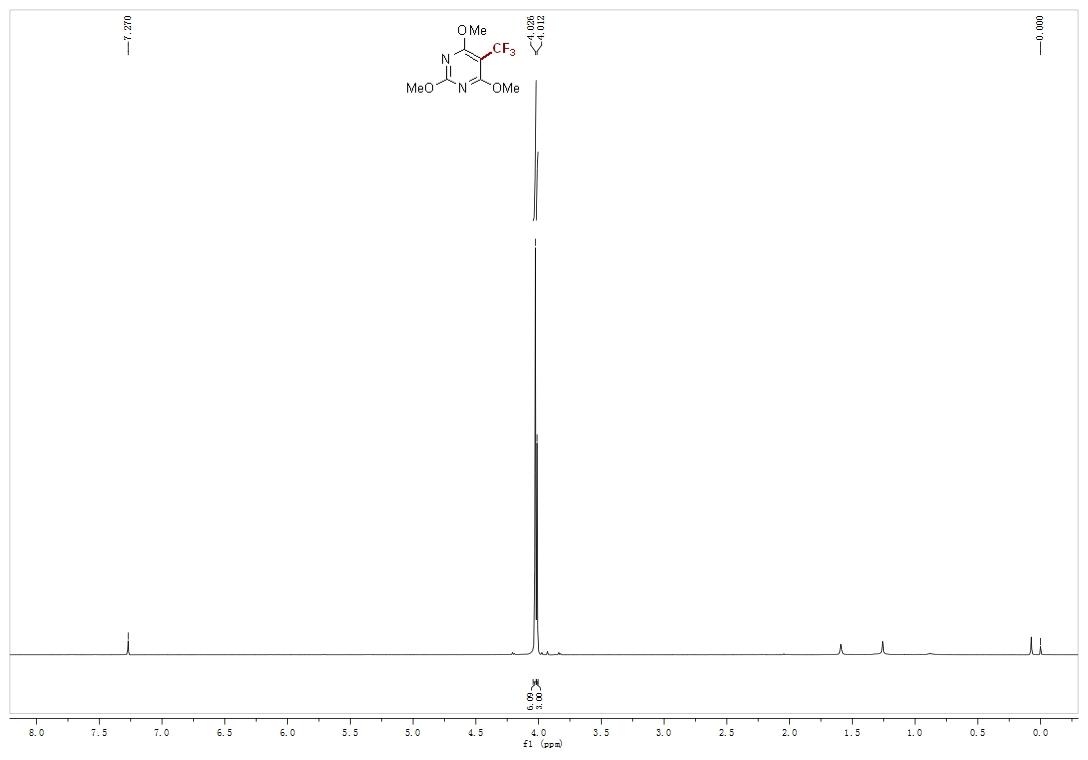


Supplementary Figure 41. ^1^H-NMR spectra of compound 2c.


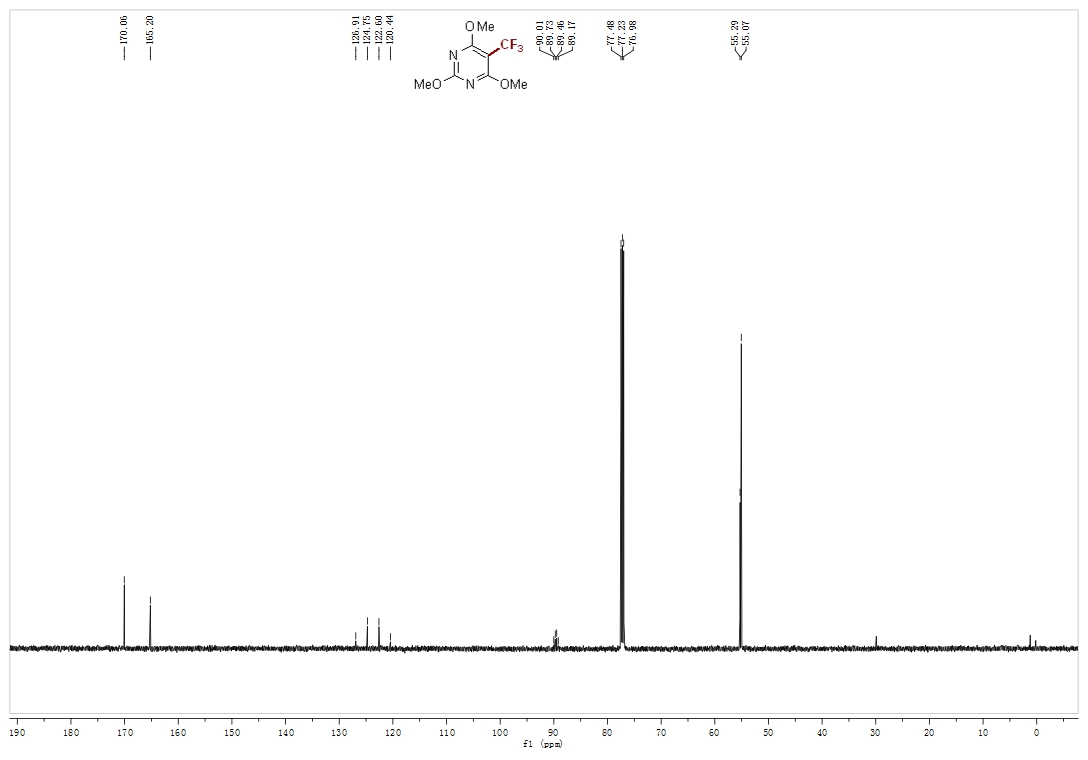


Supplementary Figure 42. ^13^C-NMR spectra of compound 2c.


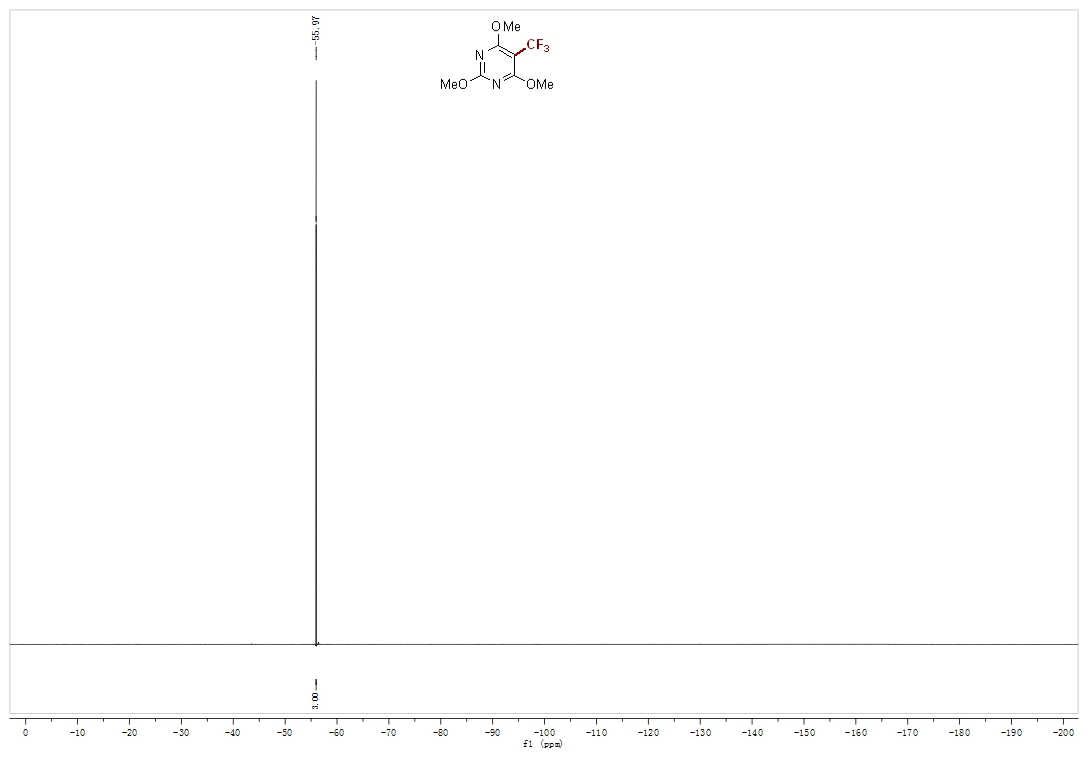


Supplementary Figure 43. ^19^F-NMR spectra of compound 2c.


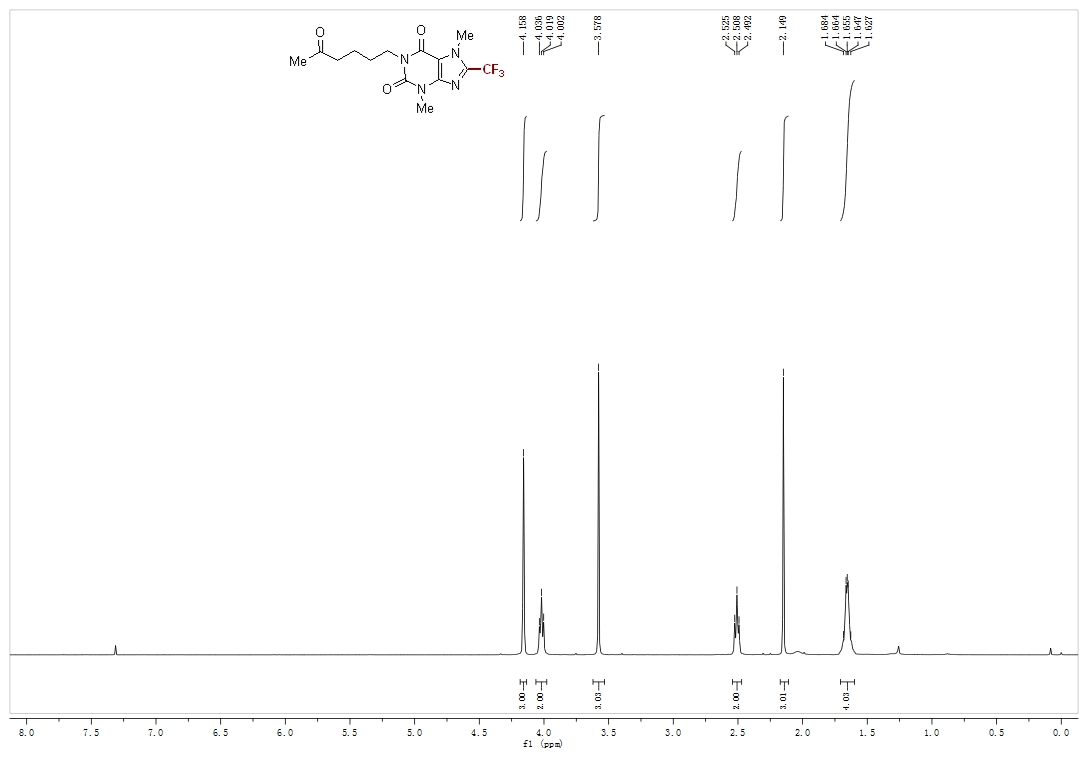


Supplementary Figure 44. ^1^H-NMR spectra of compound 2d.


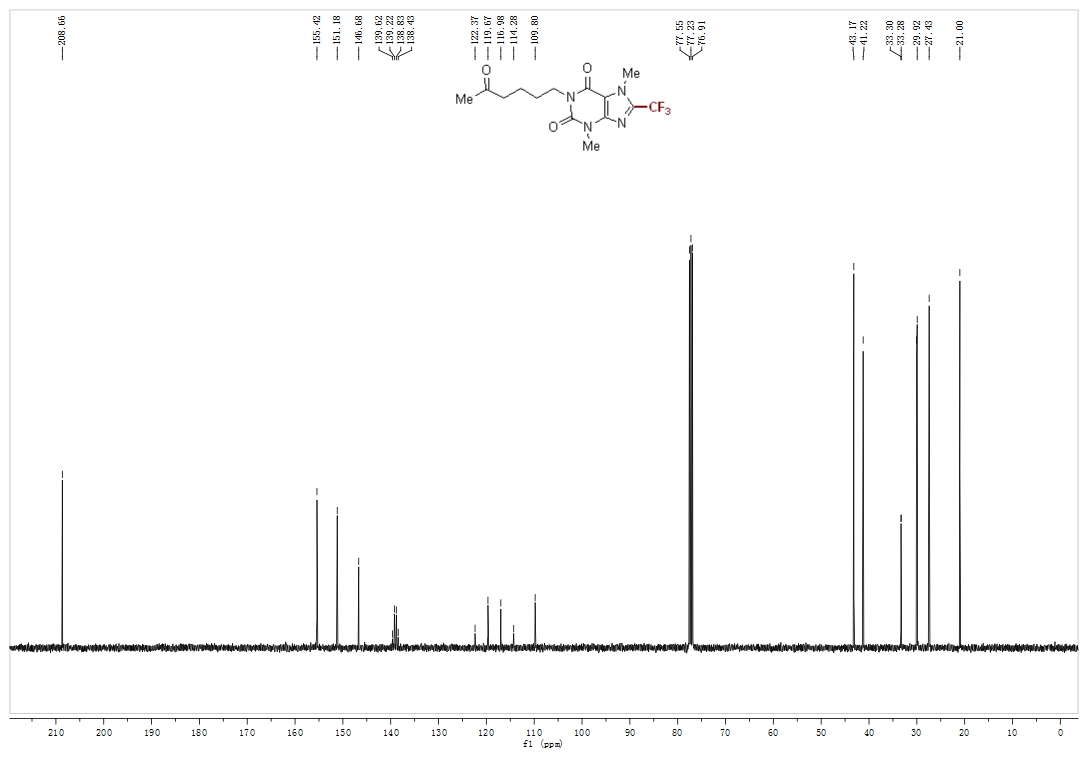


Supplementary Figure 45. ^13^C-NMR spectra of compound 2d.


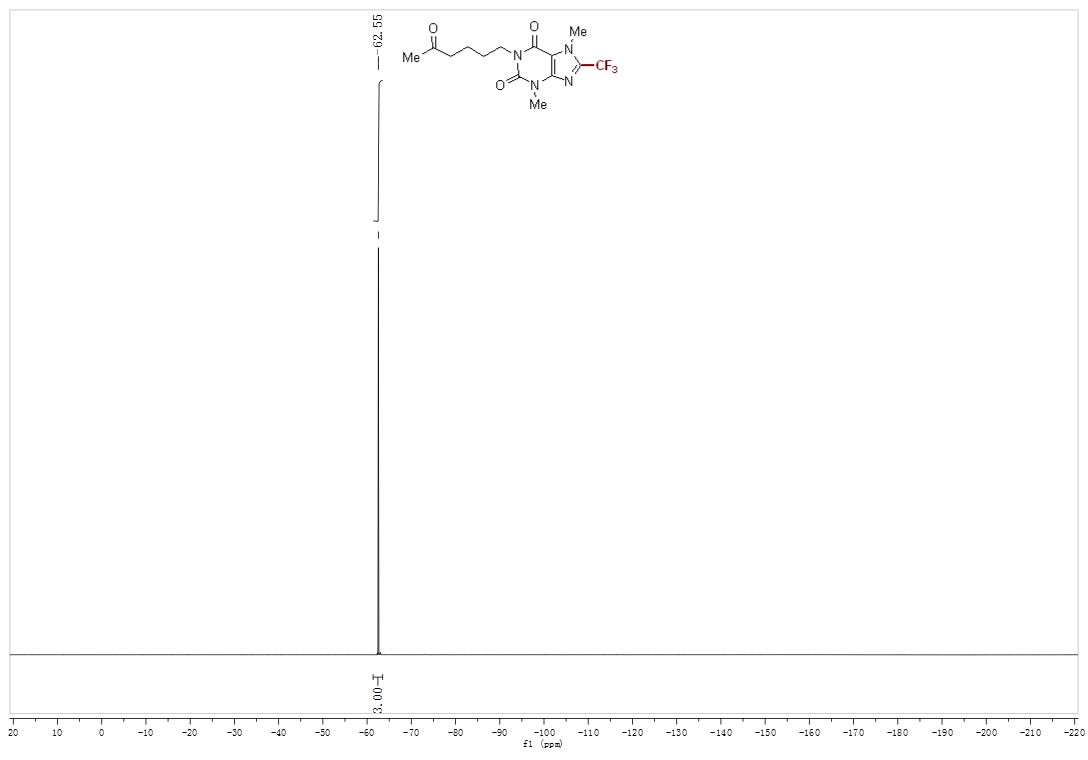


Supplementary Figure 46. ^19^F-NMR spectra of compound 2d.


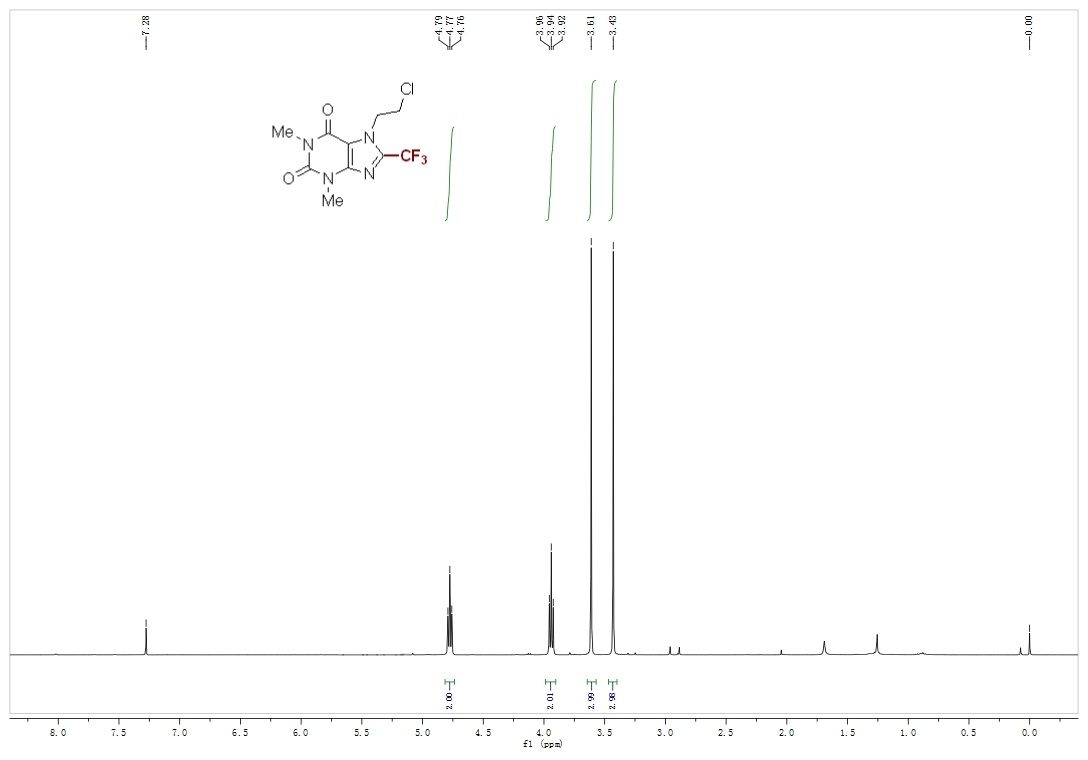


Supplementary Figure 47. ^1^H-NMR spectra of compound 2e.


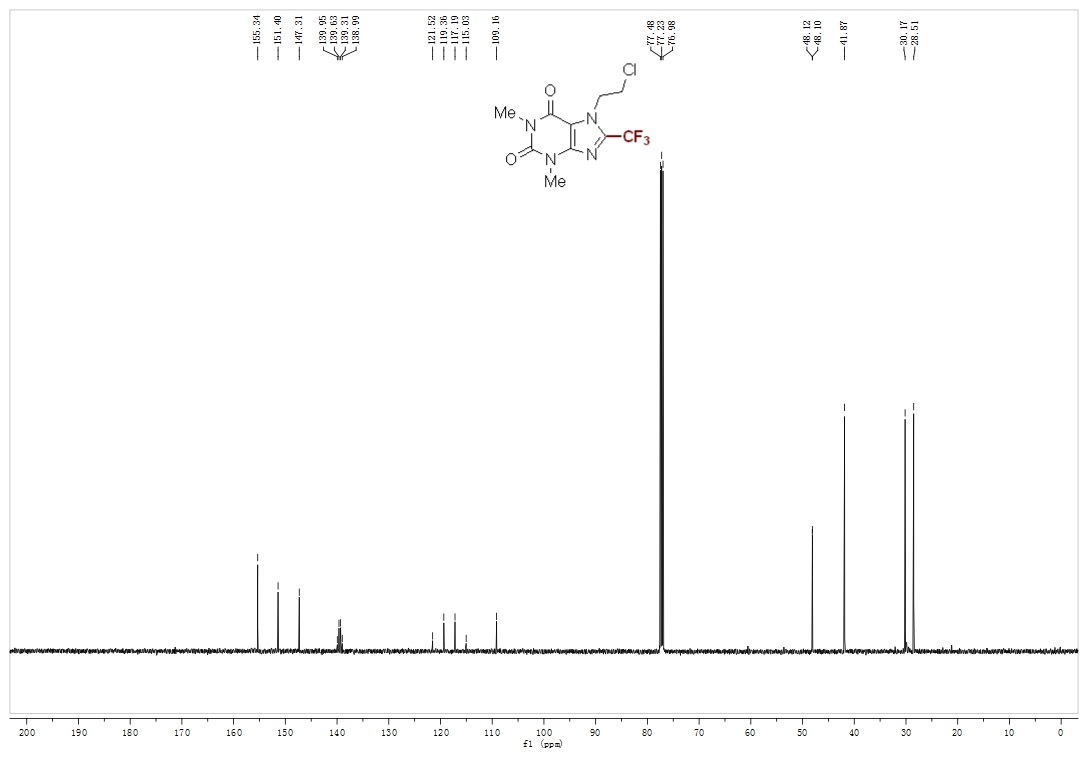


Supplementary Figure 48. ^13^C-NMR spectra of compound 2e.


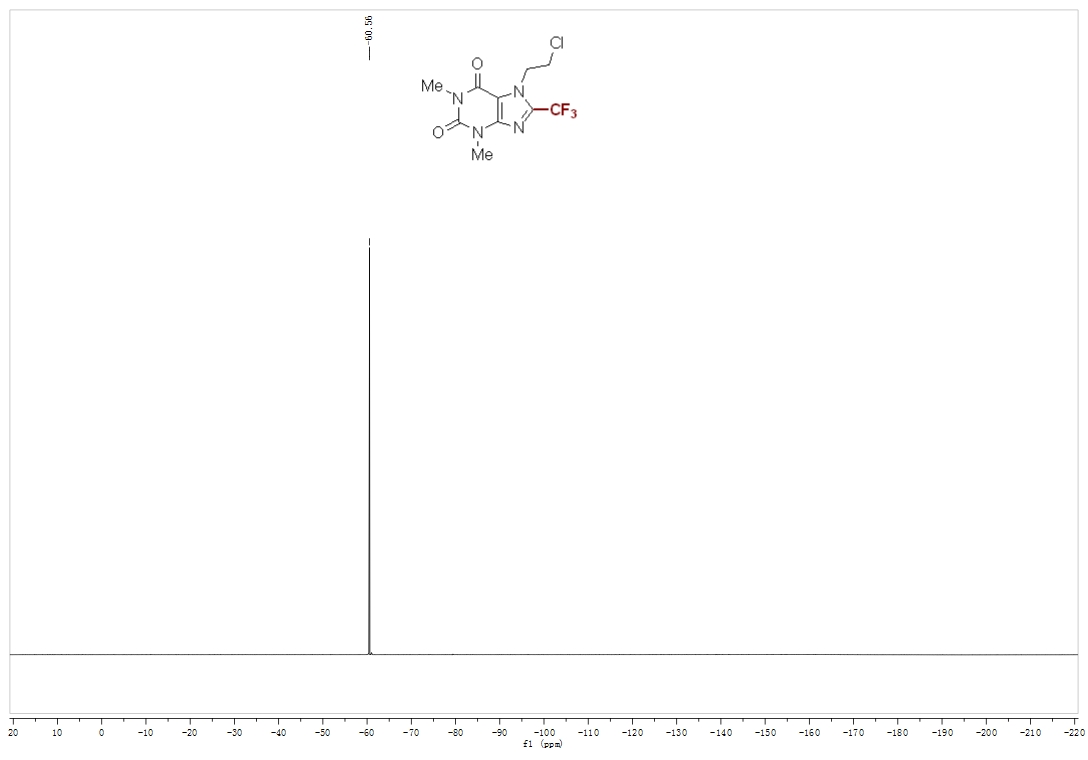


Supplementary Figure 49. ^19^F-NMR spectra of compound 2e.


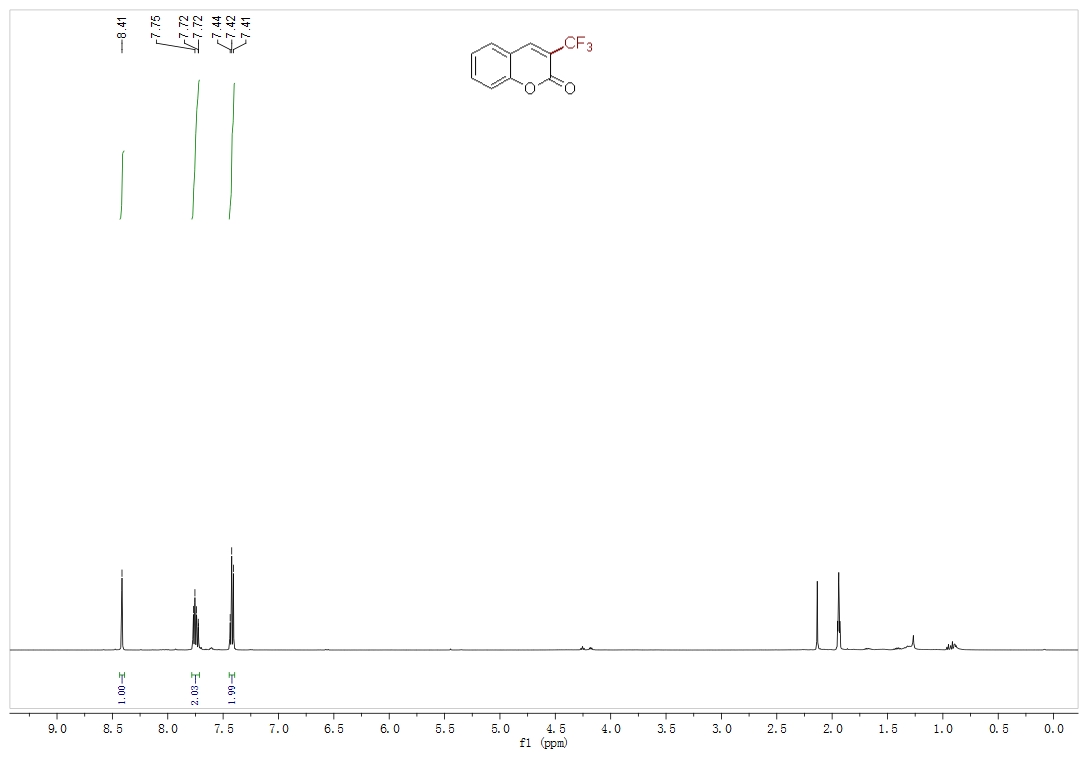


Supplementary Figure 50. ^1^H-NMR spectra of compound 2f.


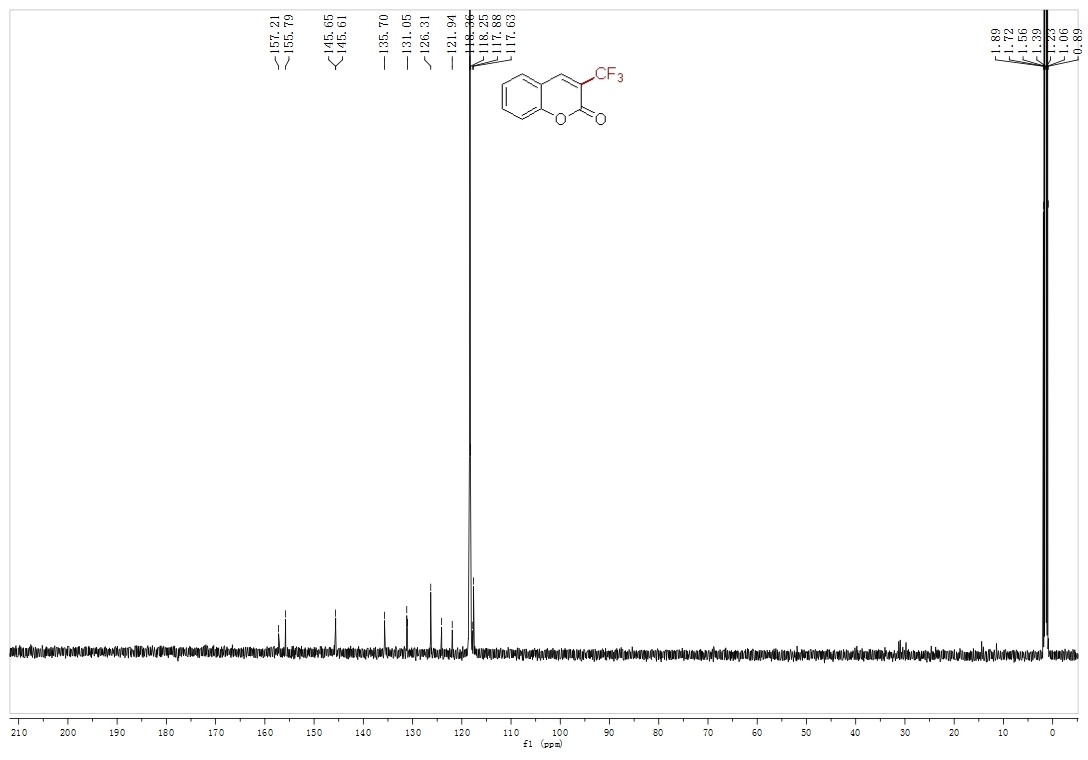


Supplementary Figure 51. ^13^C-NMR spectra of compound 2f.


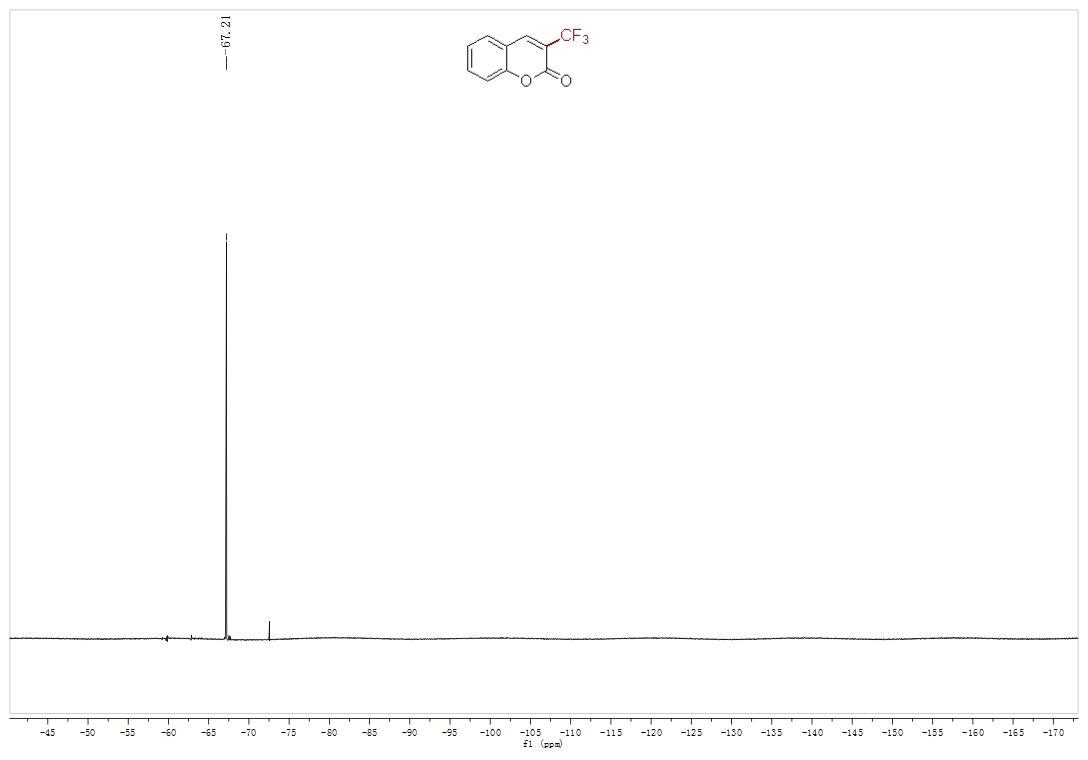


Supplementary Figure 52. ^19^F-NMR spectra of compound 2f.


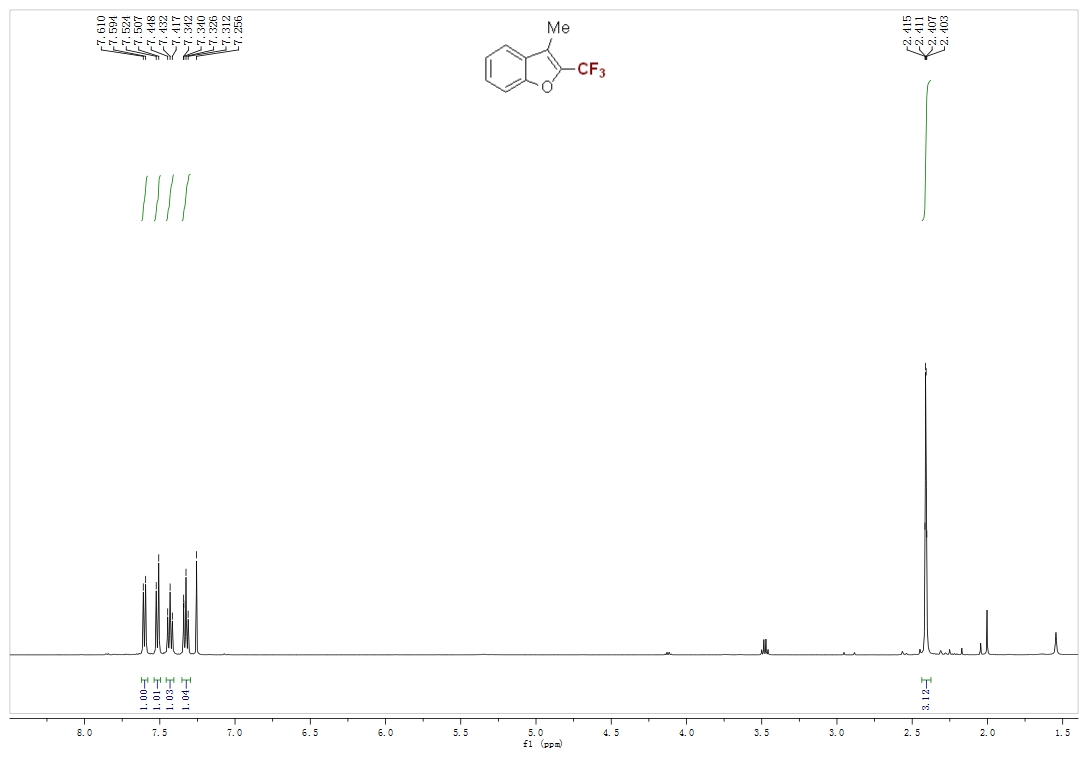


Supplementary Figure 53. ^1^H-NMR spectra of compound 2g.


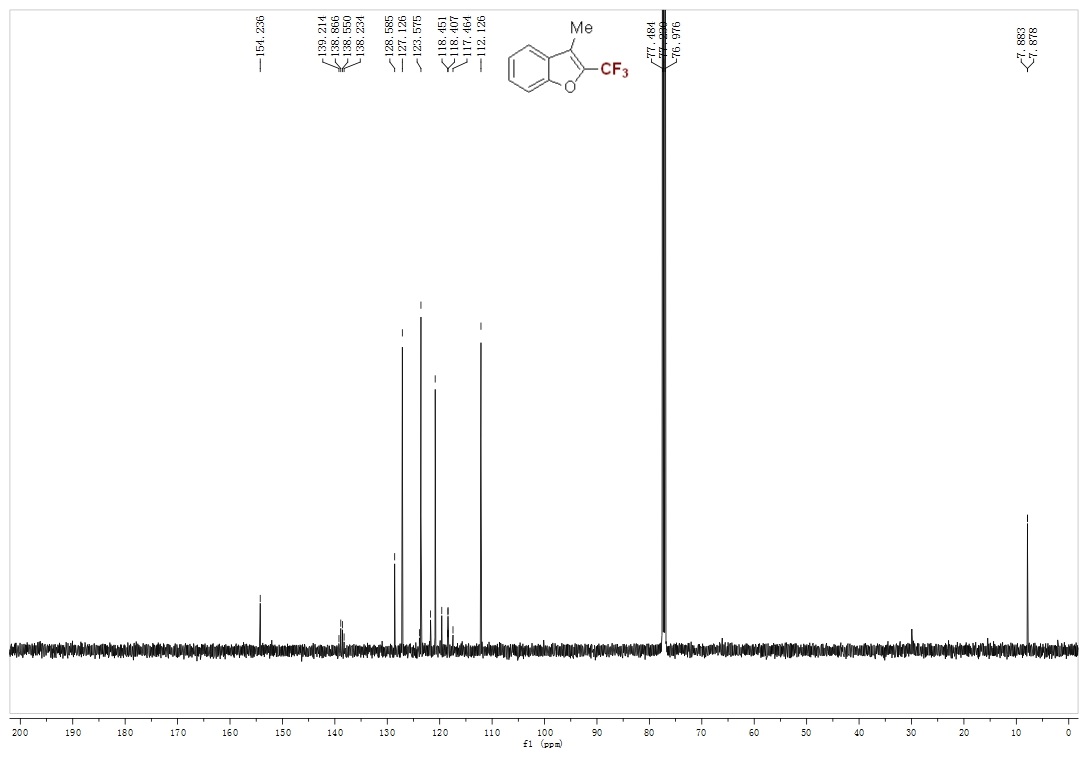


Supplementary Figure 54. ^13^C-NMR spectra of compound 2g.


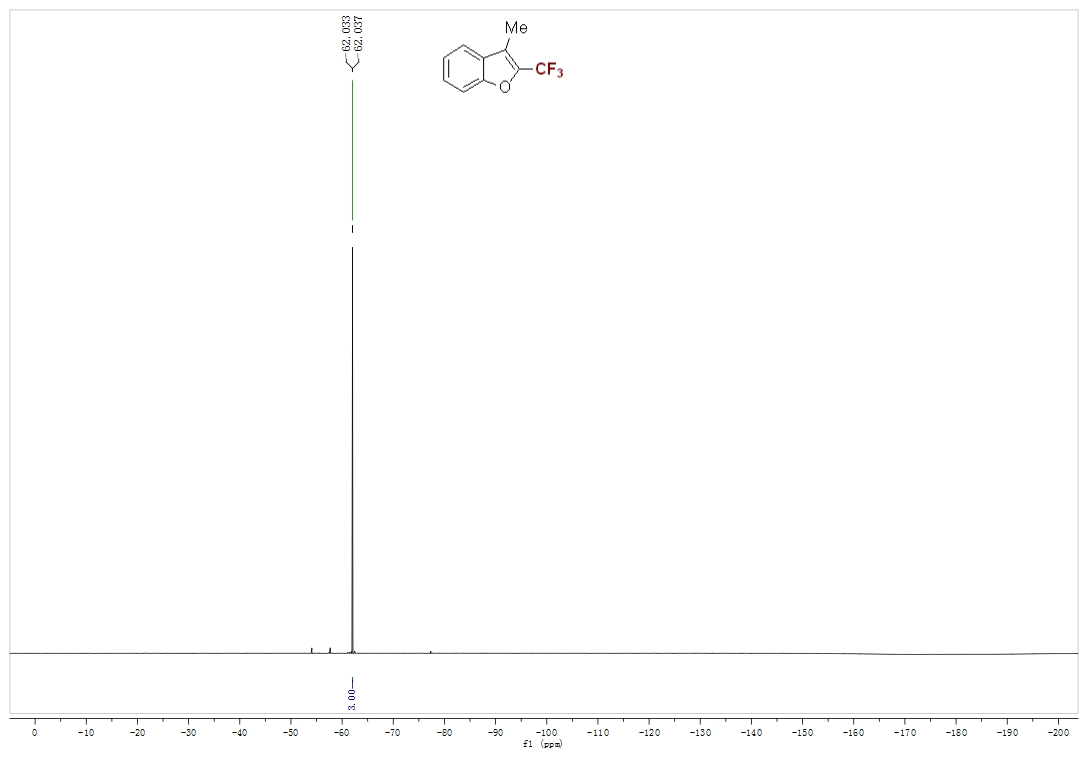


Supplementary Figure 55. ^19^F-NMR spectra of compound 2g.


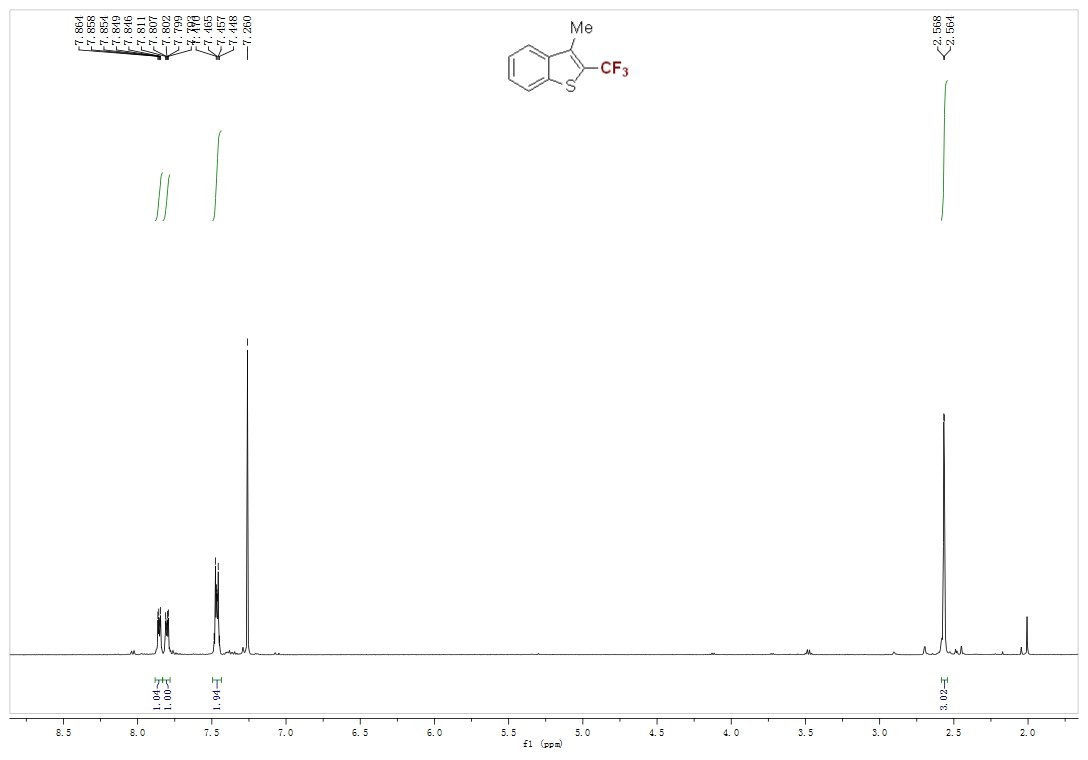


Supplementary Figure 56. ^1^H-NMR spectra of compound 2h.


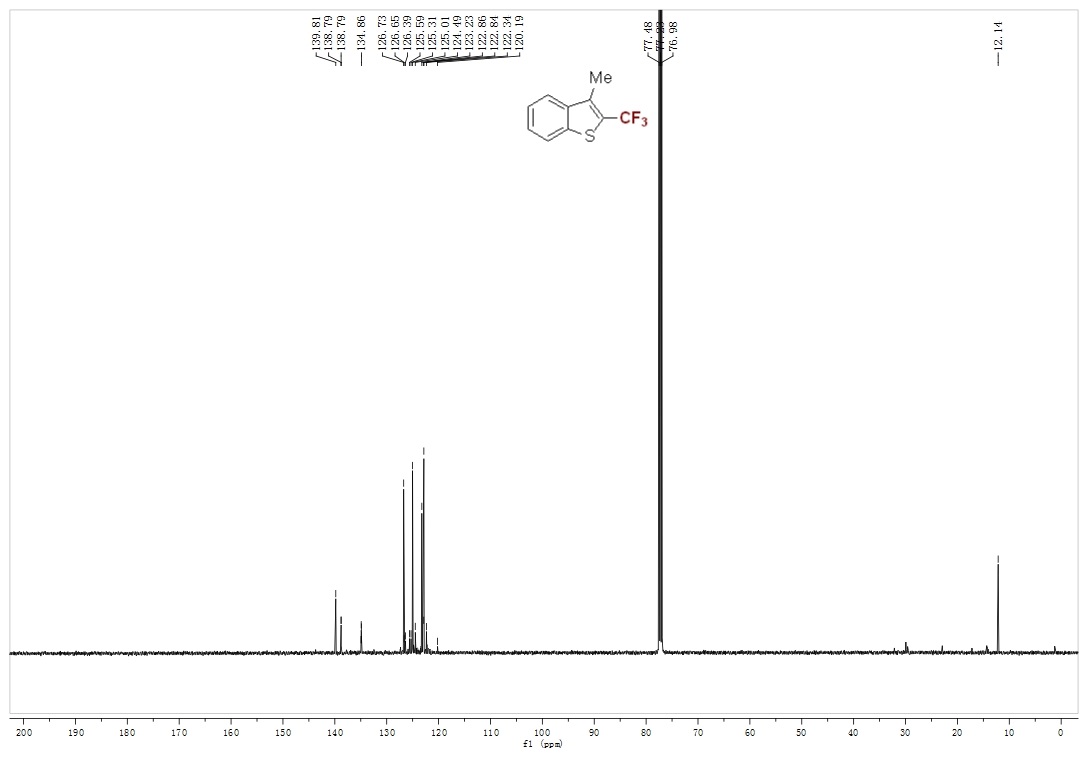


Supplementary Figure 57. ^13^C-NMR spectra of compound 2h.


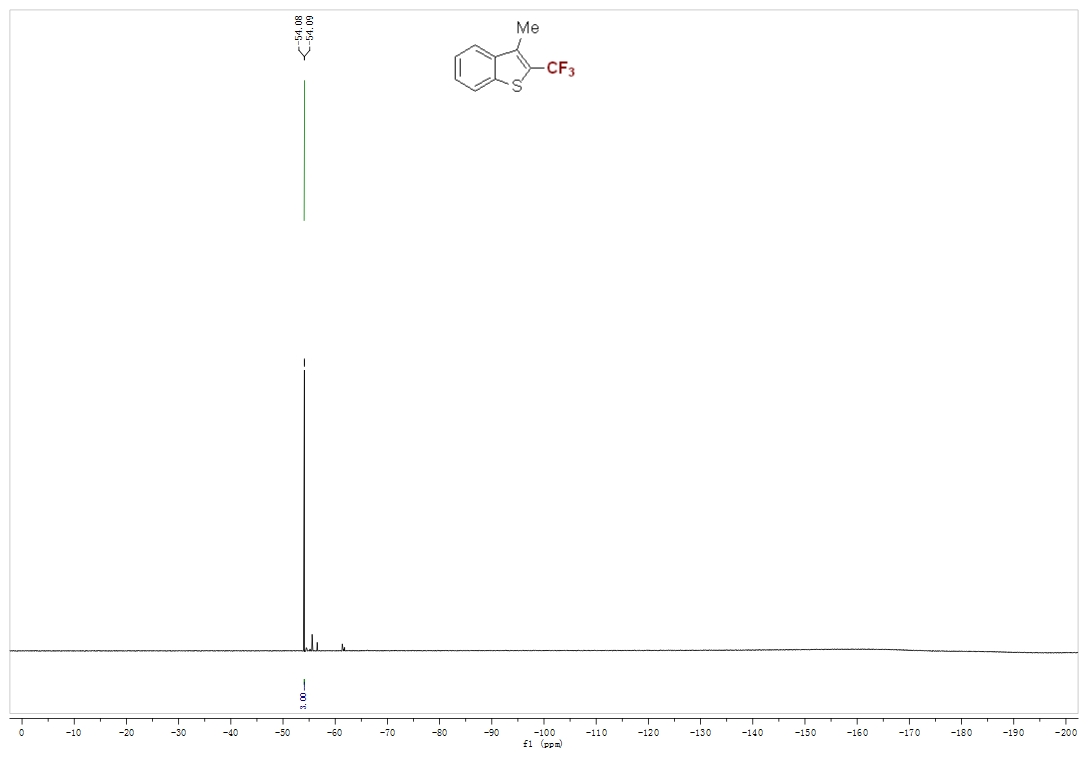


Supplementary Figure 58. ^19^F-NMR spectra of compound 2h.


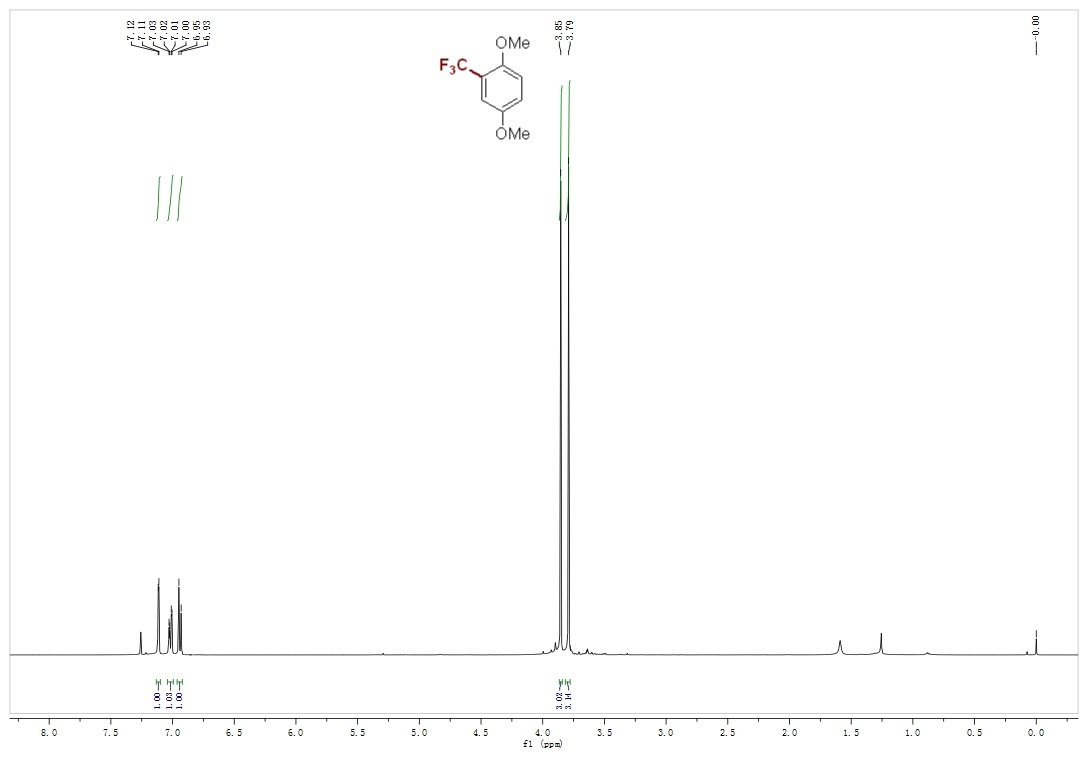


Supplementary Figure 59. ^1^H-NMR spectra of compound 2i.


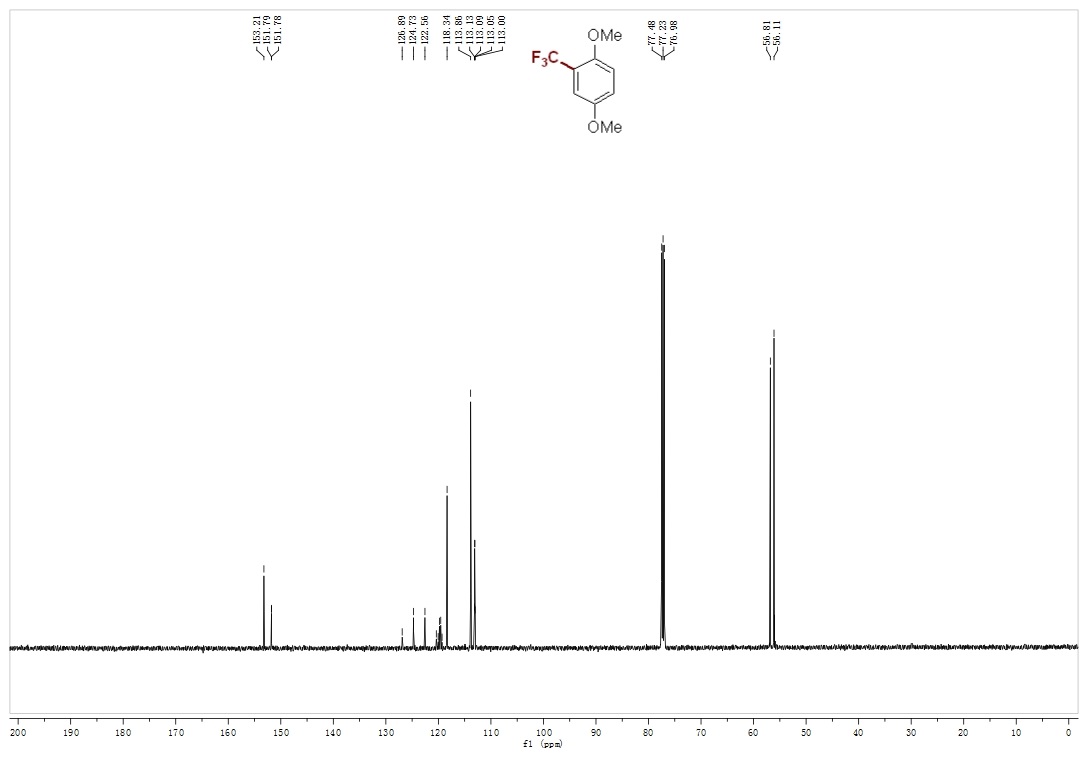


Supplementary Figure 60. ^13^C-NMR spectra of compound 2i.


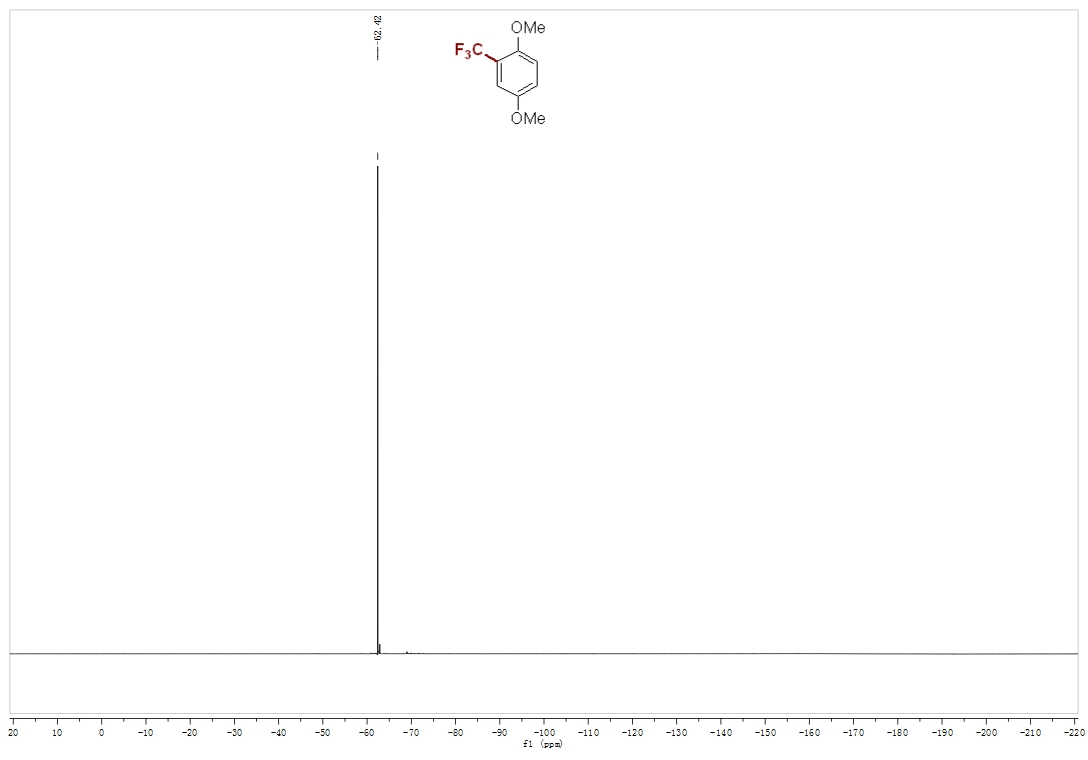


Supplementary Figure 61. ^19^F-NMR spectra of compound 2i.


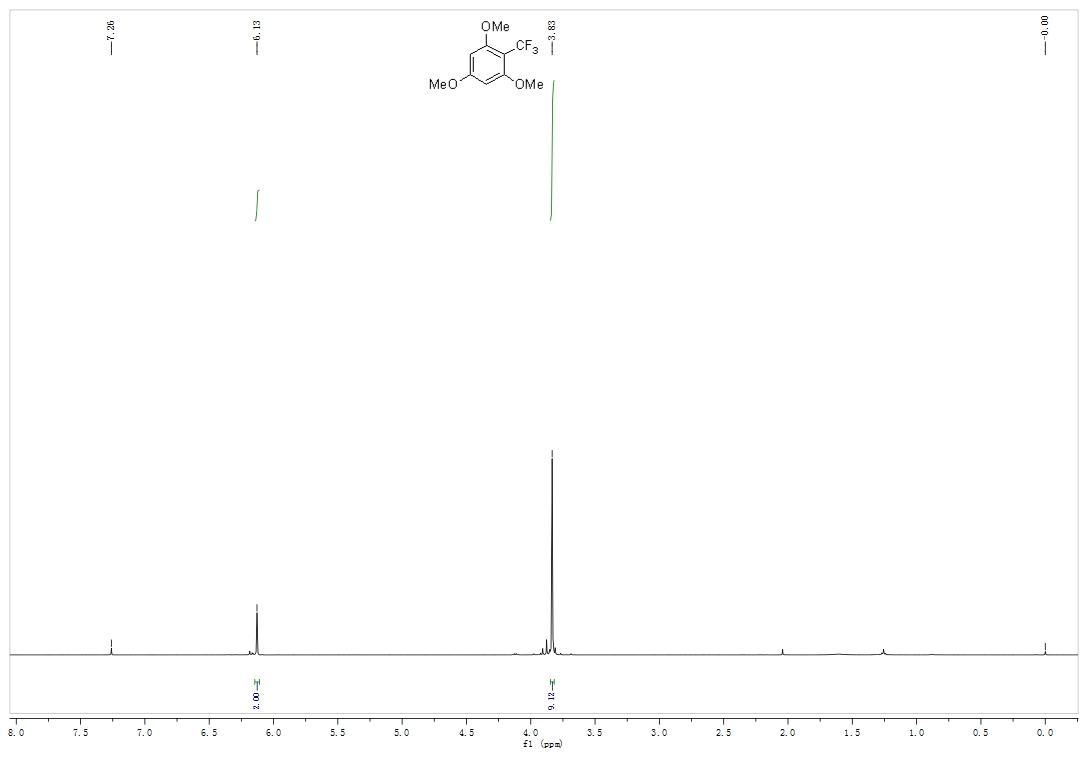


Supplementary Figure 62. ^1^H-NMR spectra of compound 2j.


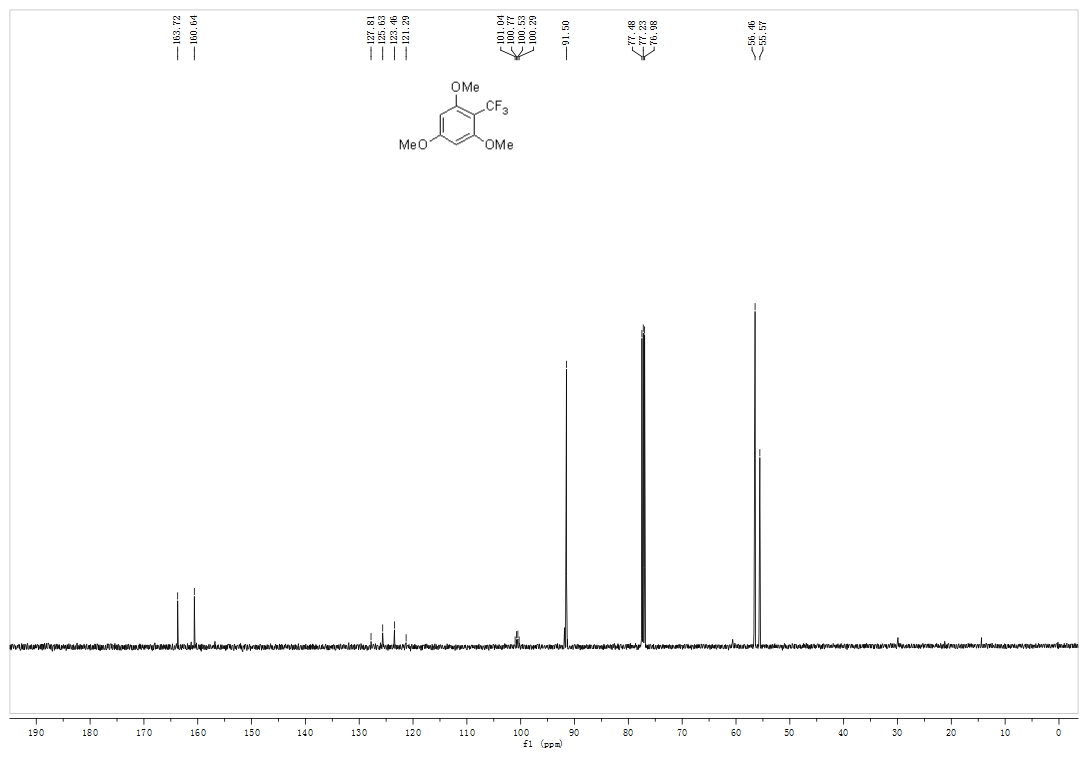


Supplementary Figure 63. ^13^C-NMR spectra of compound 2j.


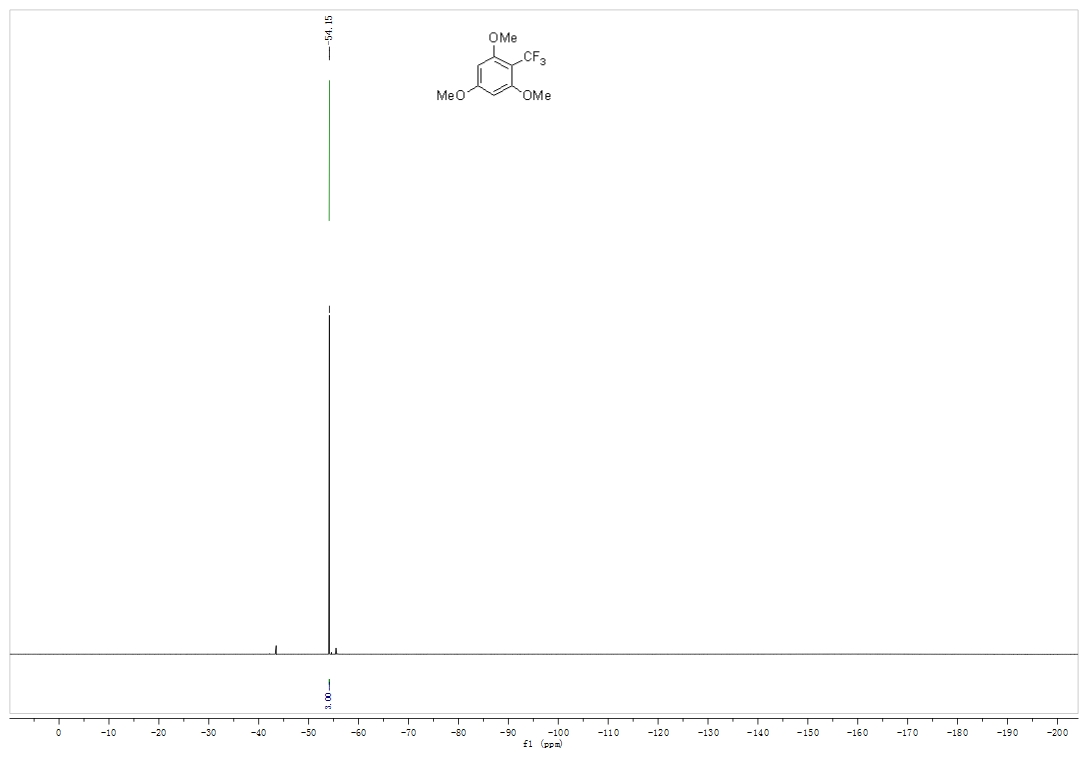


Supplementary Figure 64. ^19^F-NMR spectra of compound 2j.


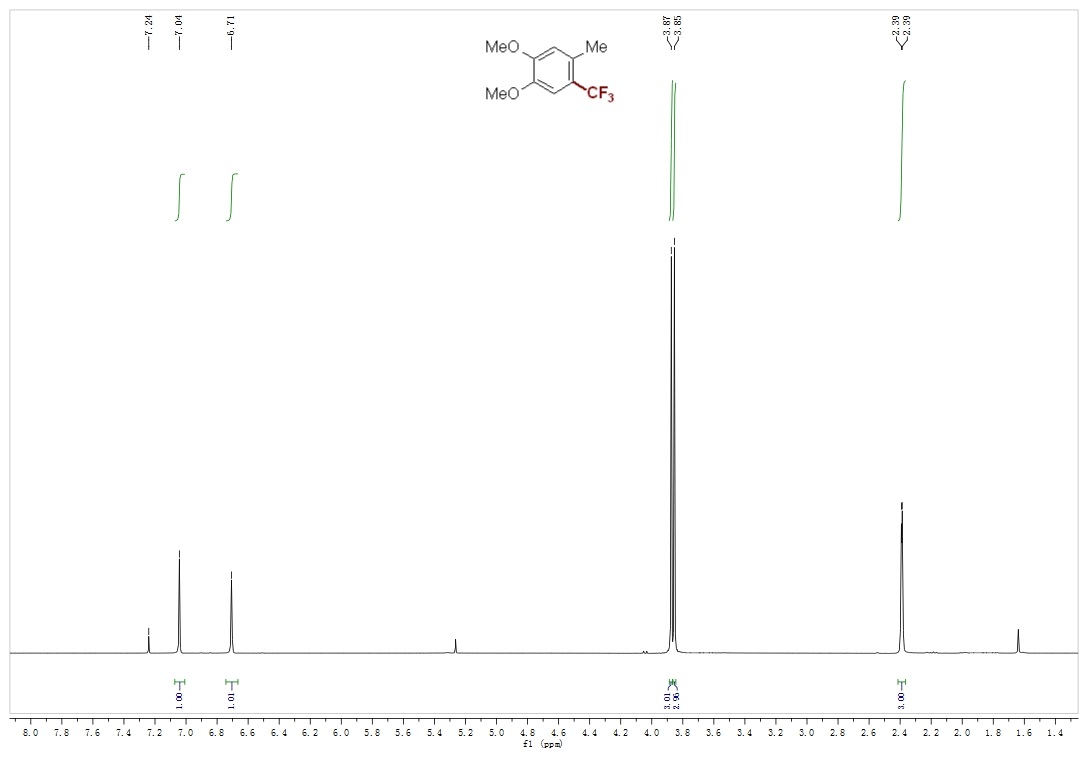


Supplementary Figure 65. ^1^H-NMR spectra of compound 2k.


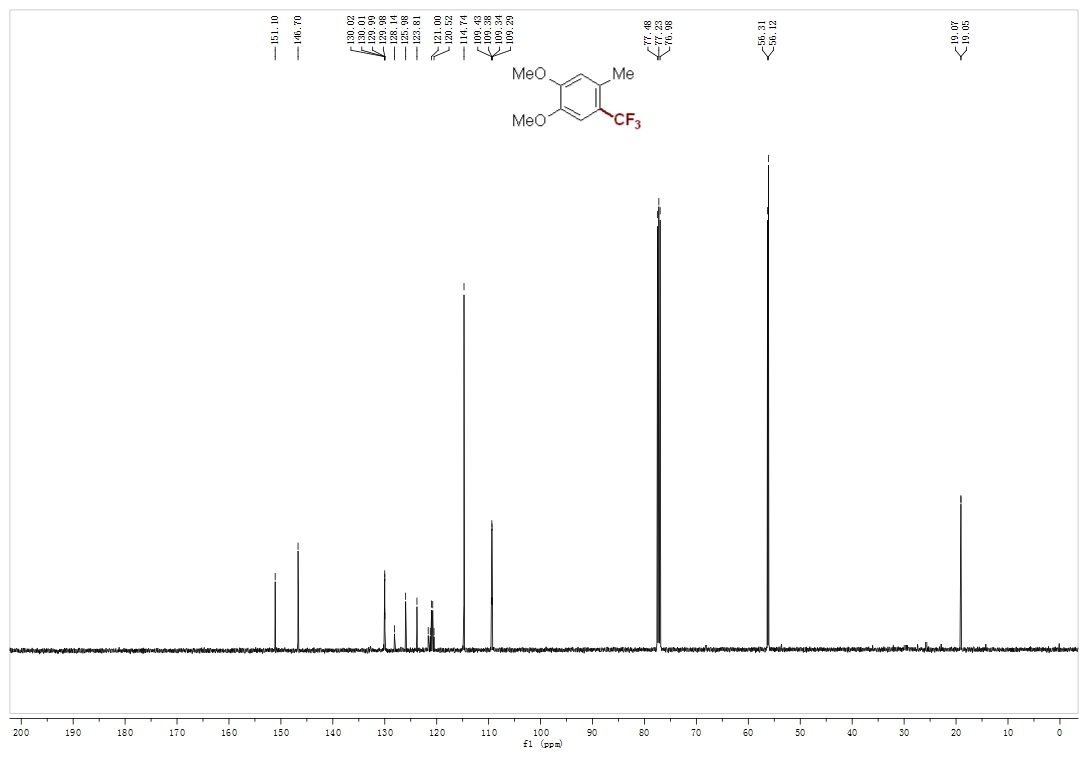


Supplementary Figure 66. ^13^C-NMR spectra of compound 2k.


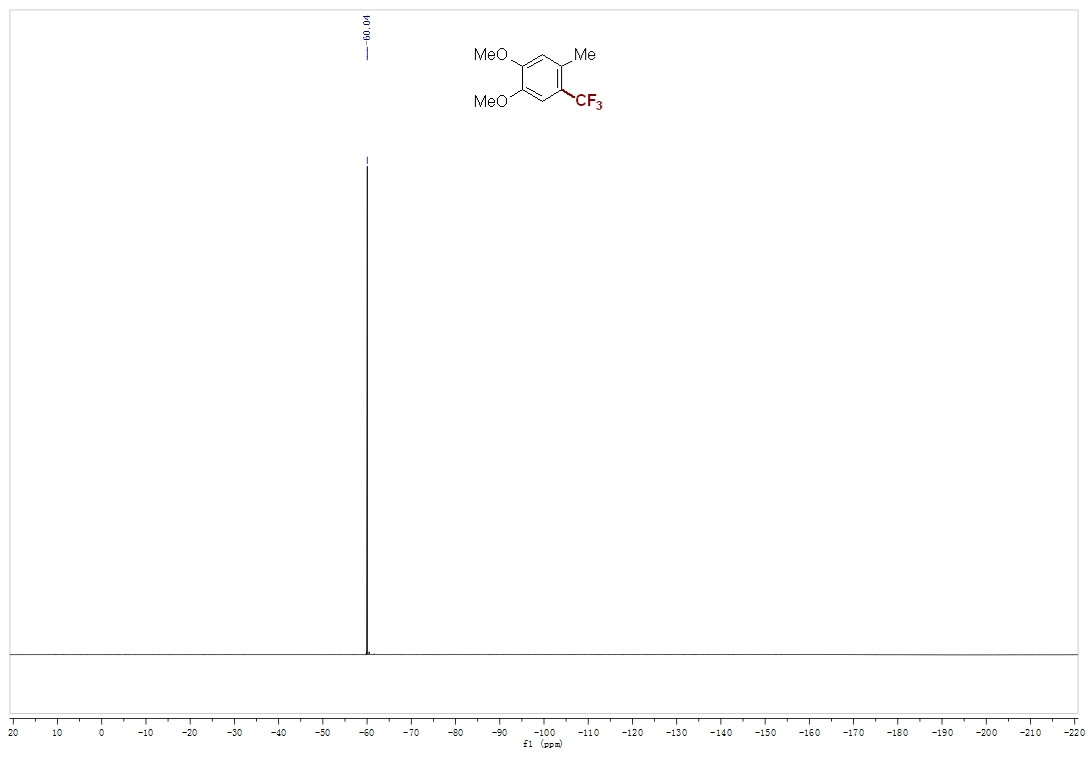


Supplementary Figure 67. ^19^F-NMR spectra of compound 2k.


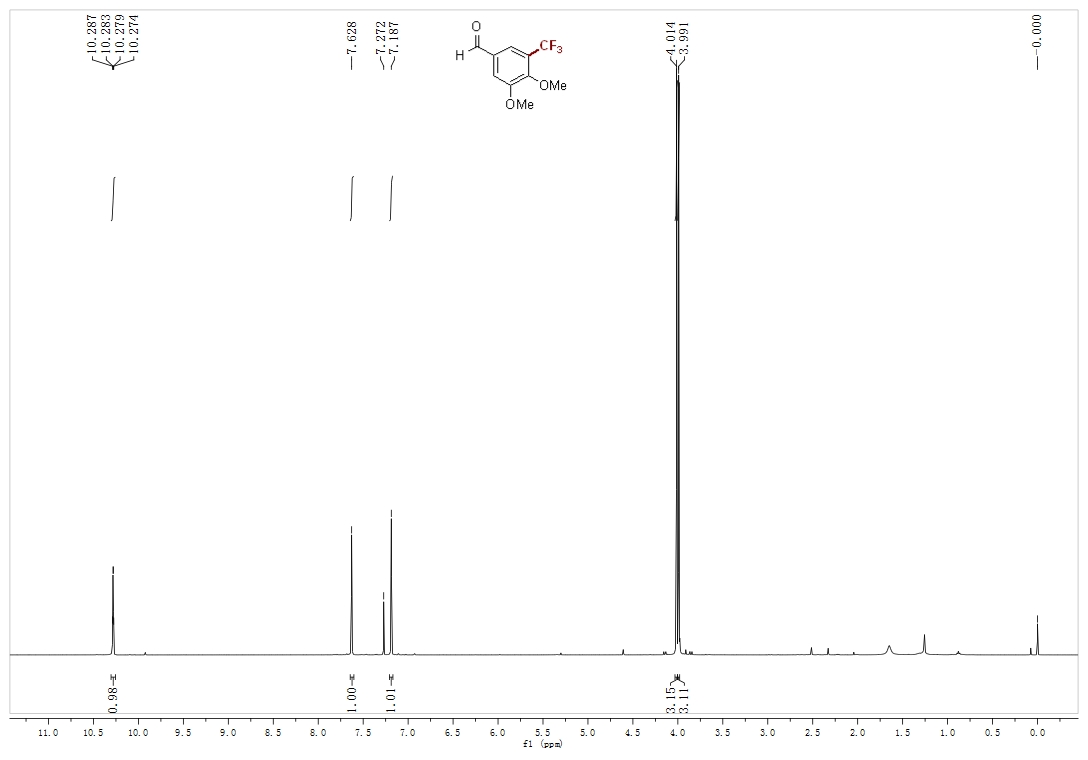


Supplementary Figure 68. ^1^H-NMR spectra of compound 2l.


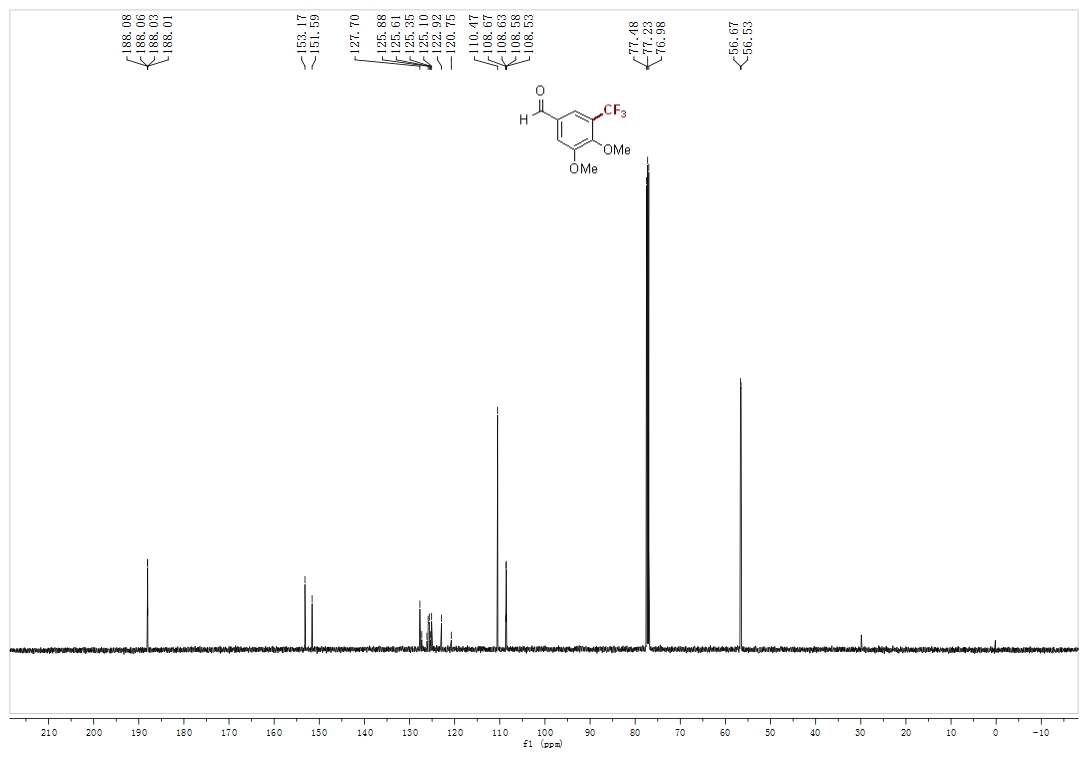


Supplementary Figure 69. ^13^C-NMR spectra of compound 2l.


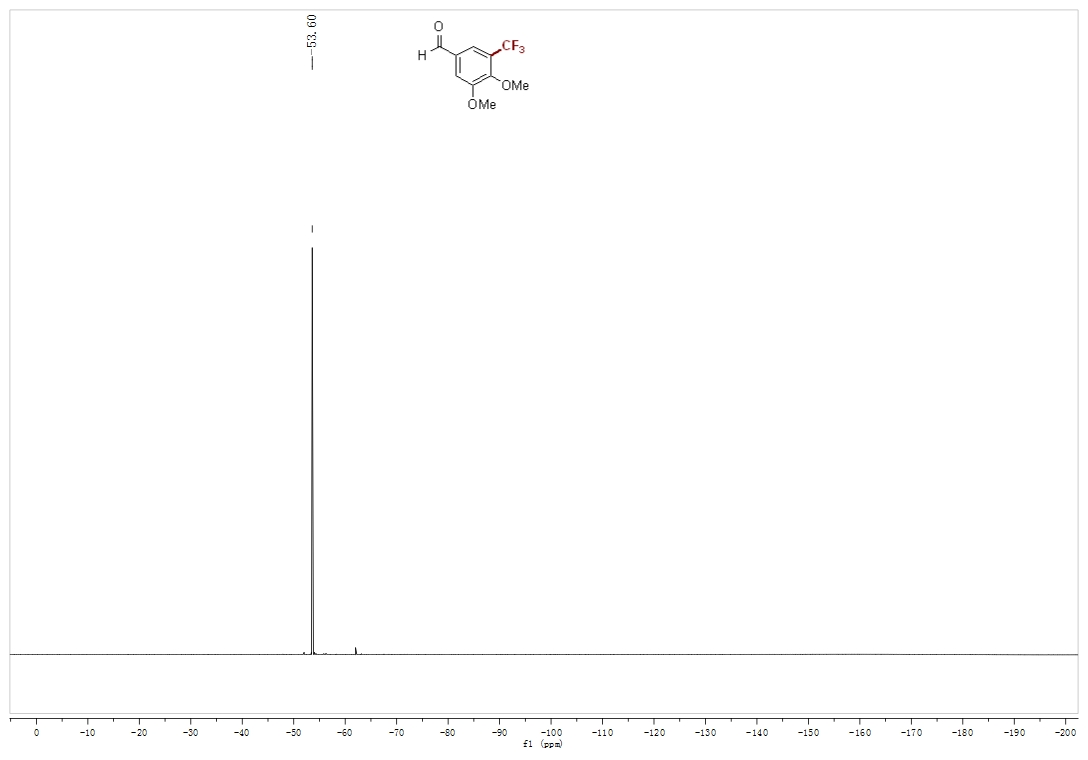


Supplementary Figure 70. ^19^F-NMR spectra of compound 2l.


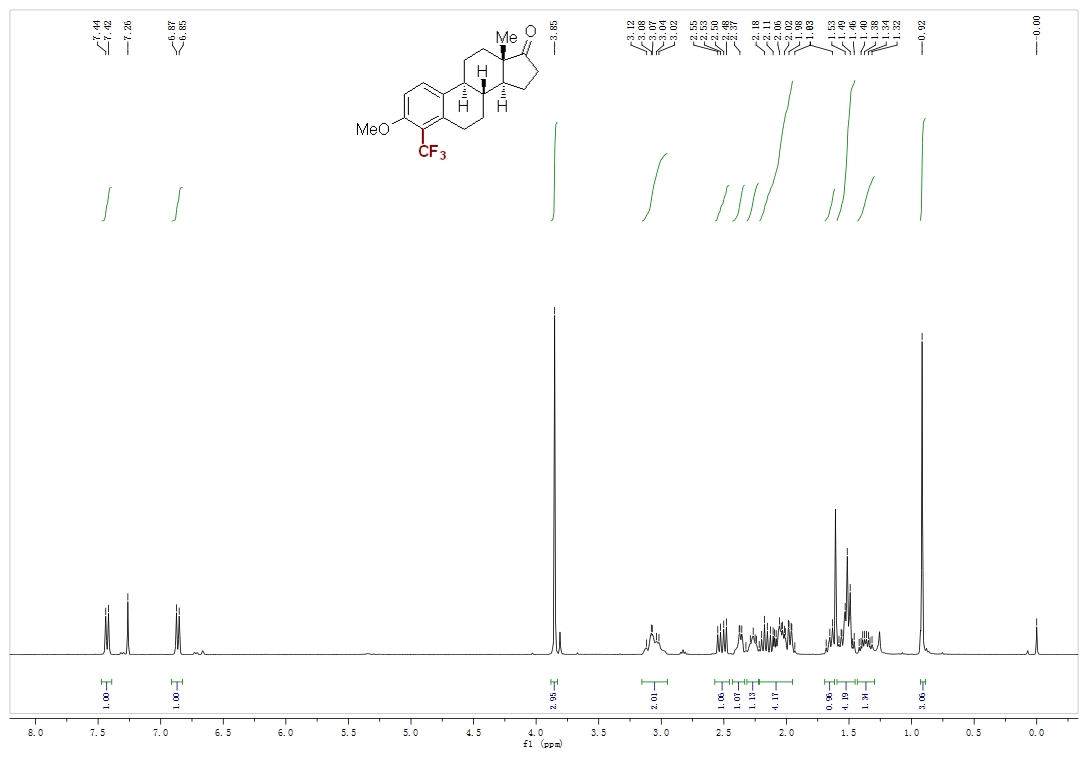


Supplementary Figure 71. ^1^H-NMR spectra of compound 2m.


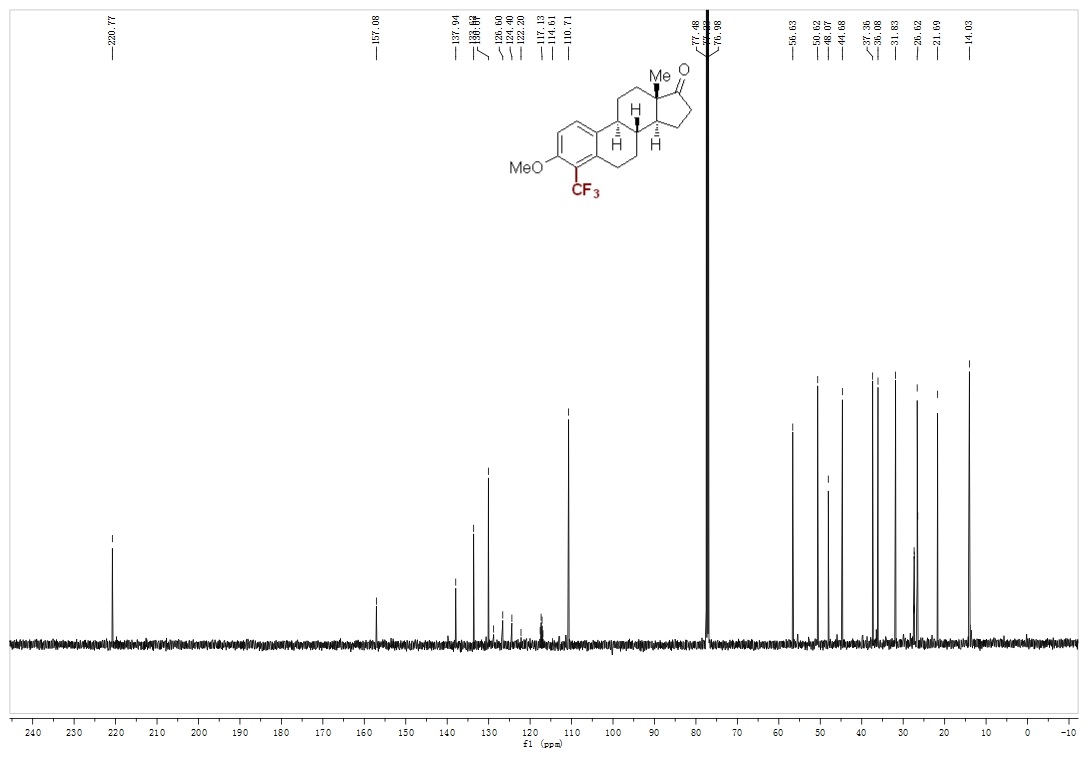


Supplementary Figure 72. ^13^C-NMR spectra of compound 2m.


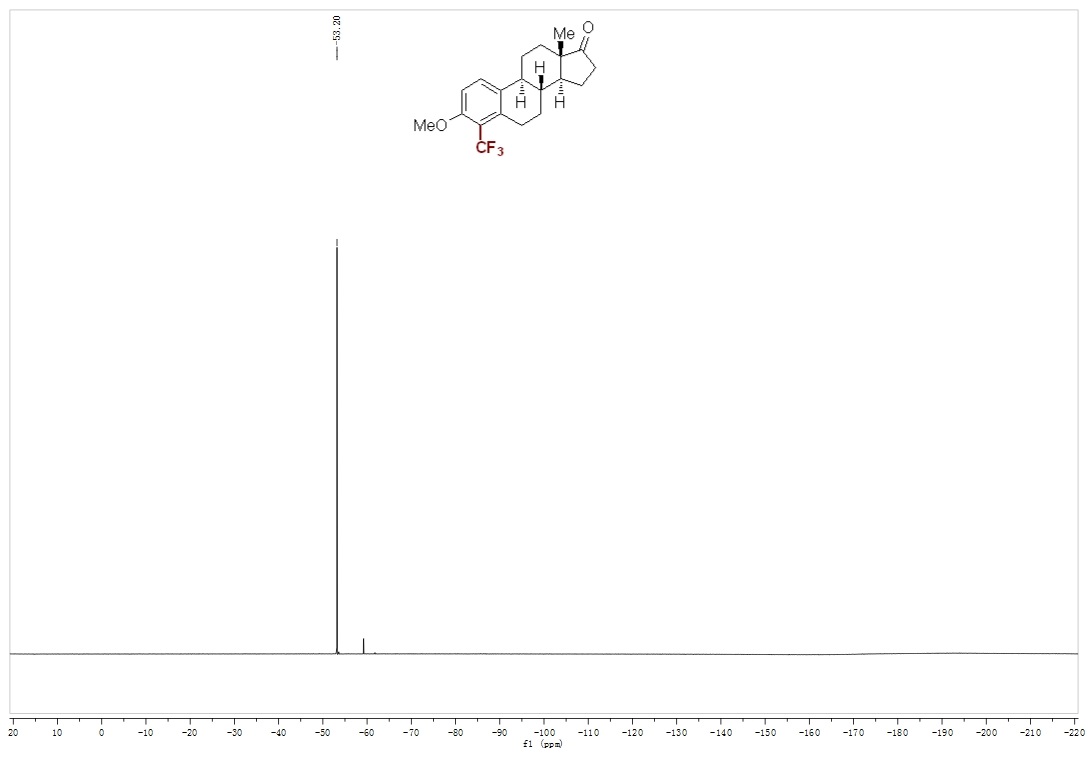


Supplementary Figure 73. ^19^F-NMR spectra of compound 2m.


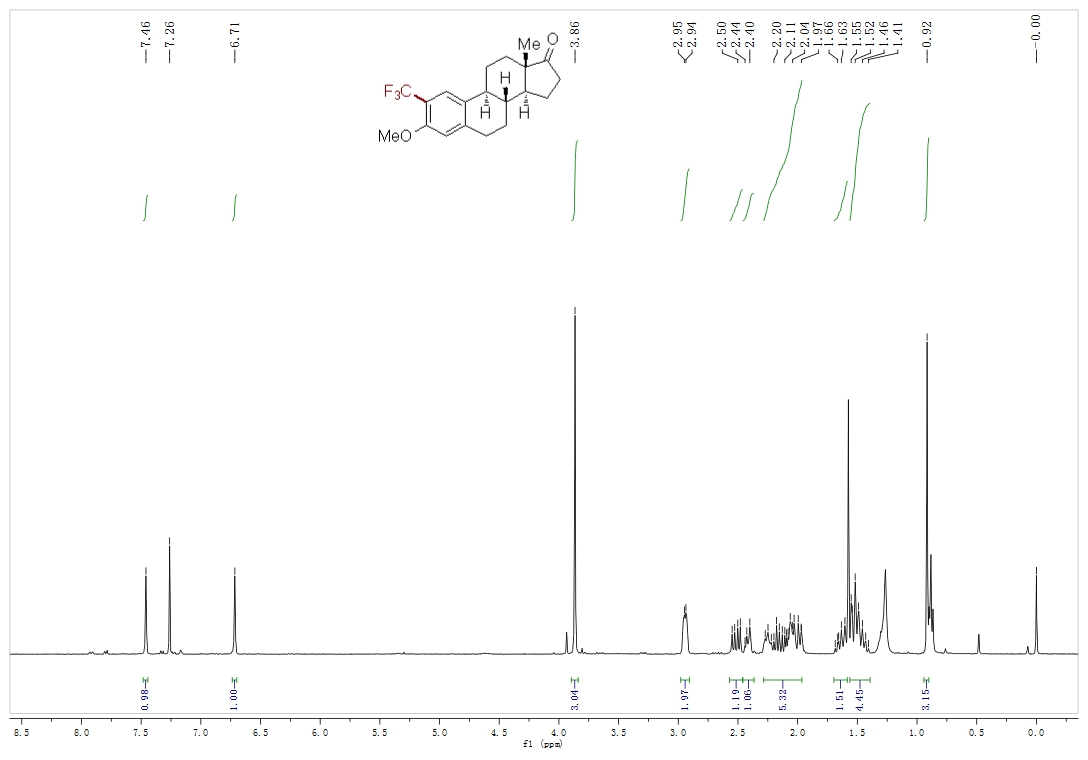


Supplementary Figure 74. ^1^H-NMR spectra of compound 2m'.


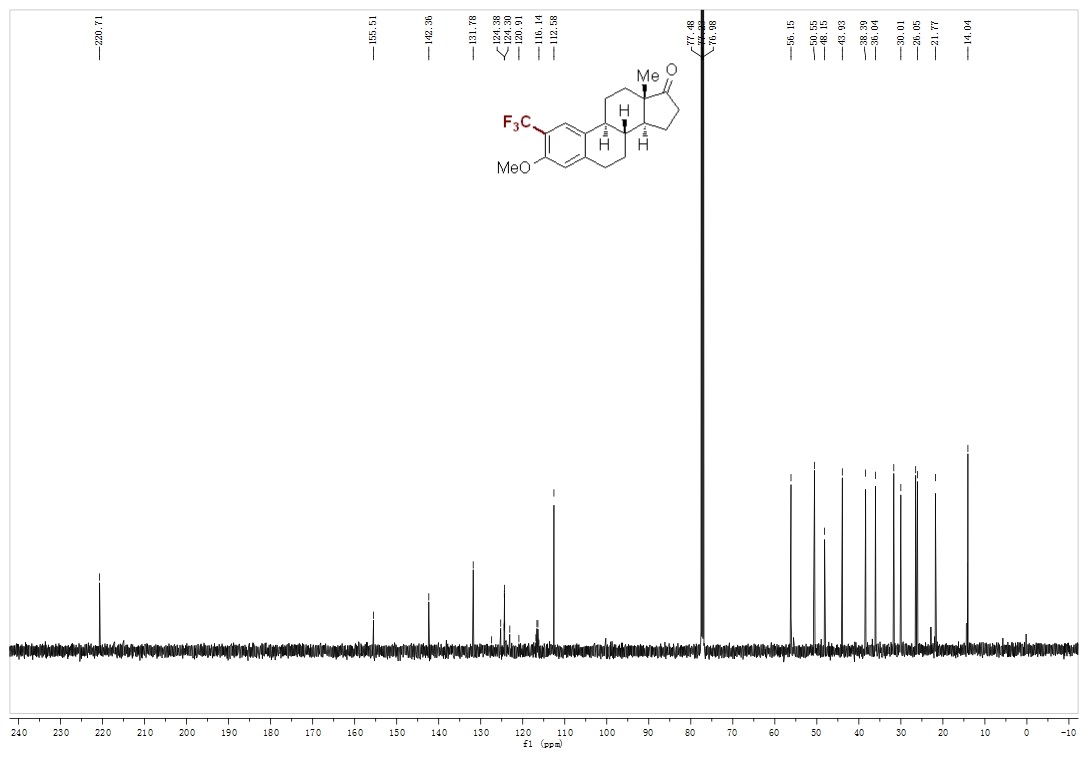


Supplementary Figure 75. ^13^C-NMR spectra of compound 2m'.


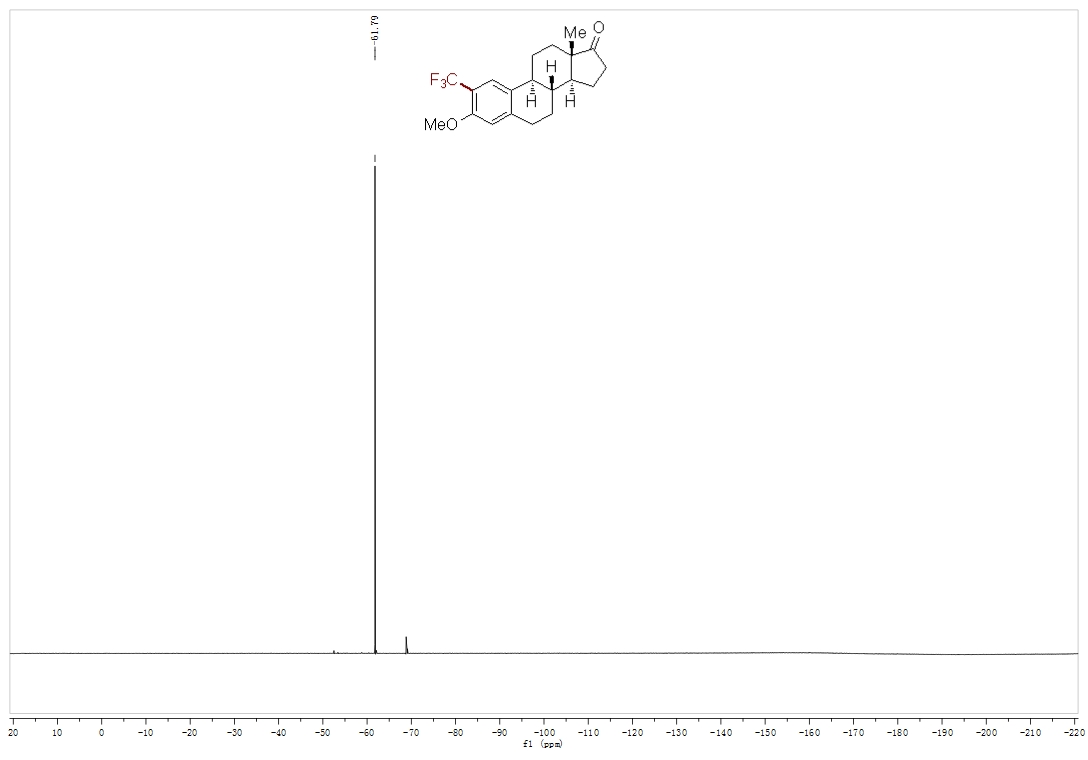


Supplementary Figure 76. ^19^F-NMR spectra of compound 2m'.


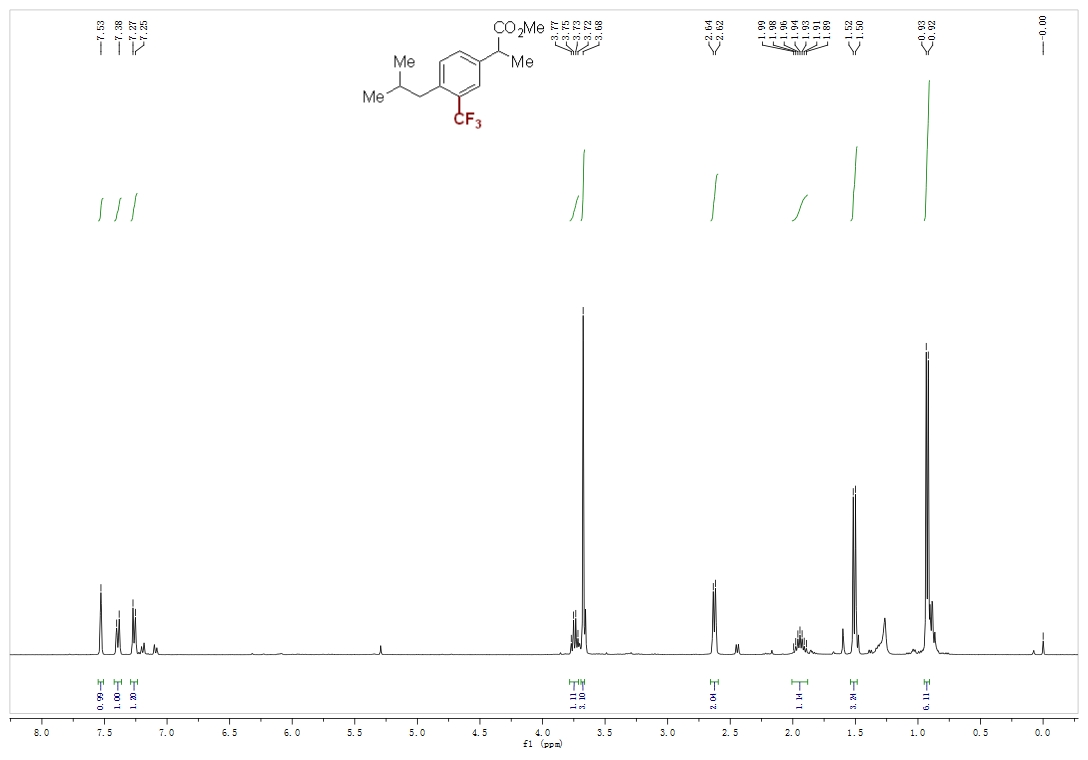


Supplementary Figure 77. ^1^H-NMR spectra of compound 2n.


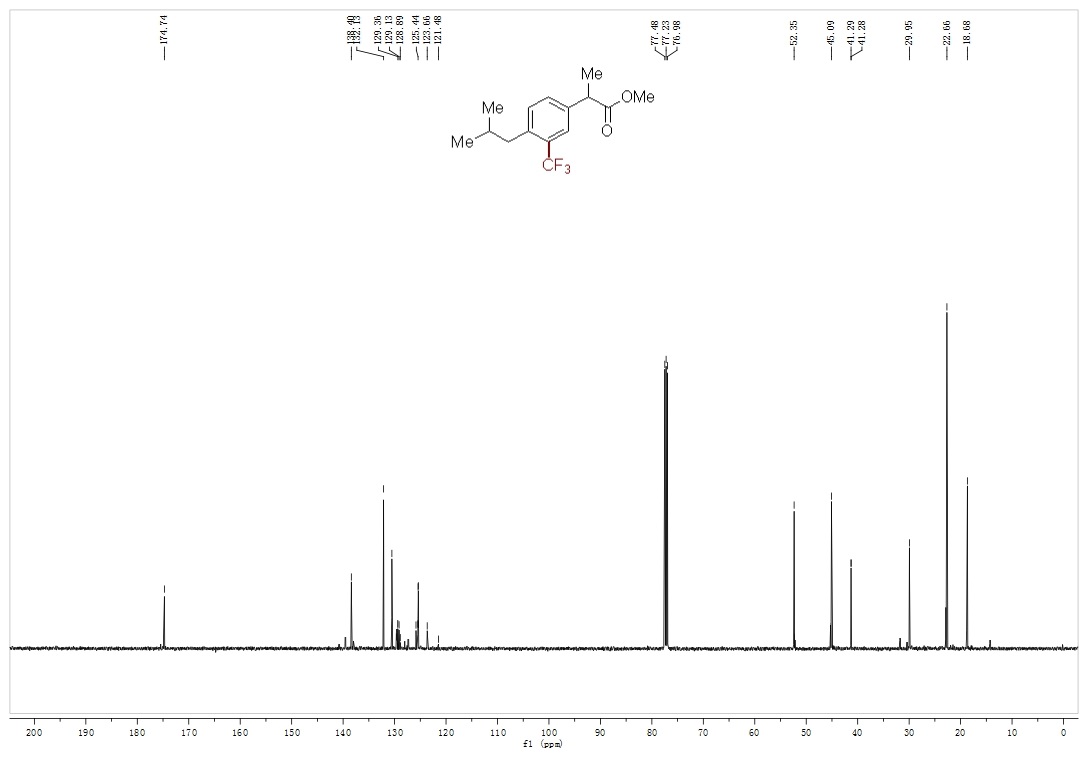


Supplementary Figure 78. ^13^C-NMR spectra of compound 2n.


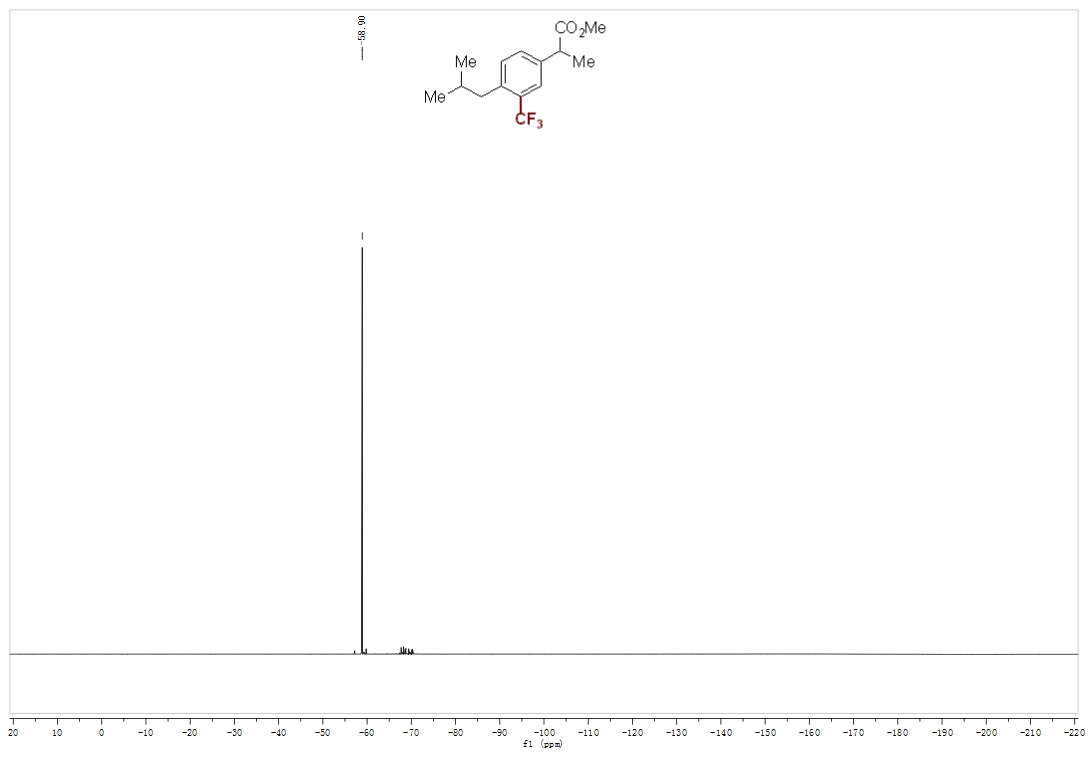


Supplementary Figure 79. ^19^F-NMR spectra of compound 2n.


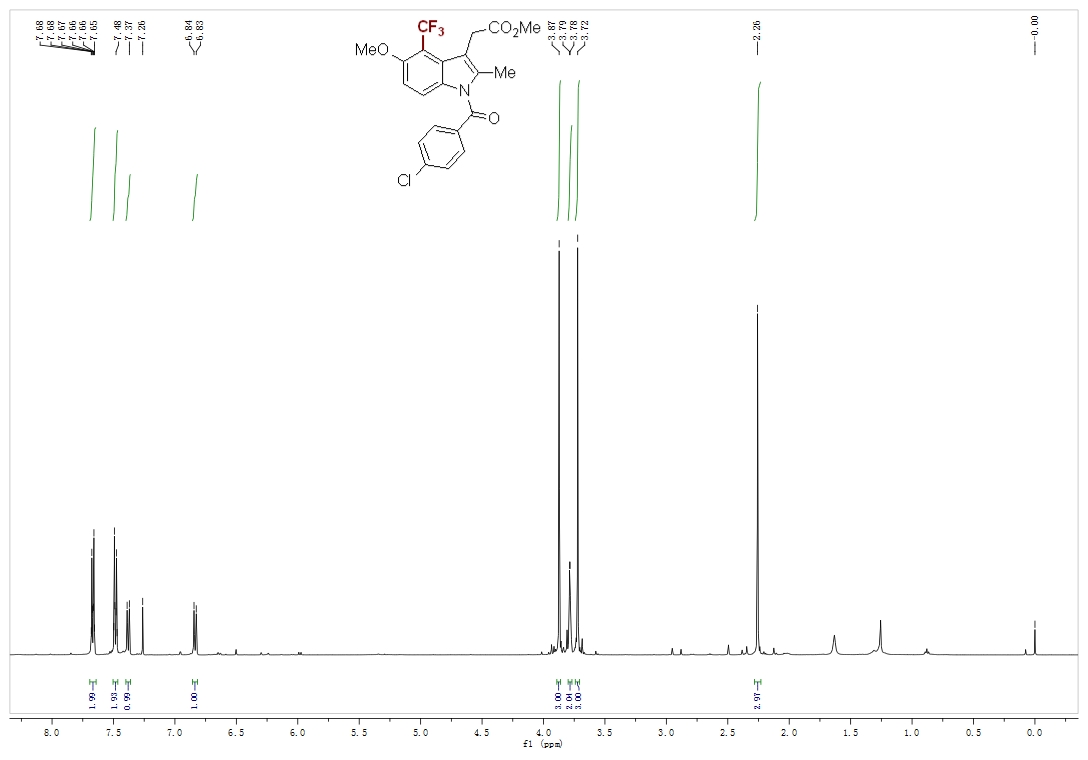


Supplementary Figure 80. ^1^H-NMR spectra of compound 2o.


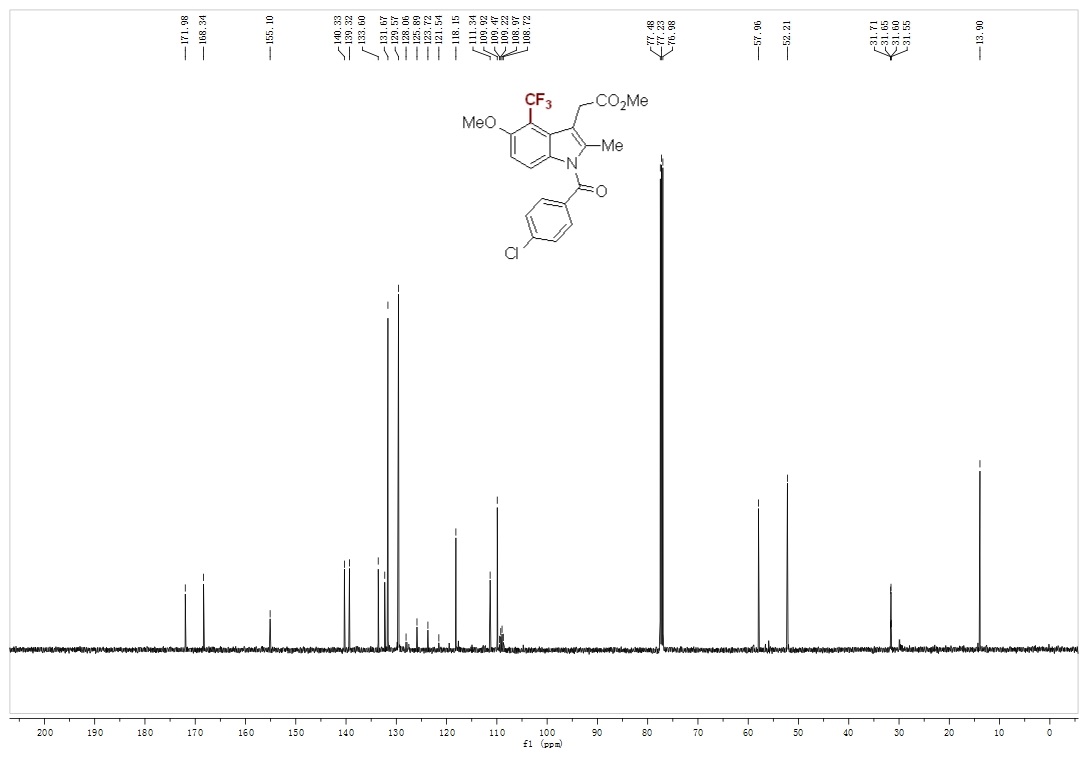


Supplementary Figure 81. ^13^C-NMR spectra of compound 2o.


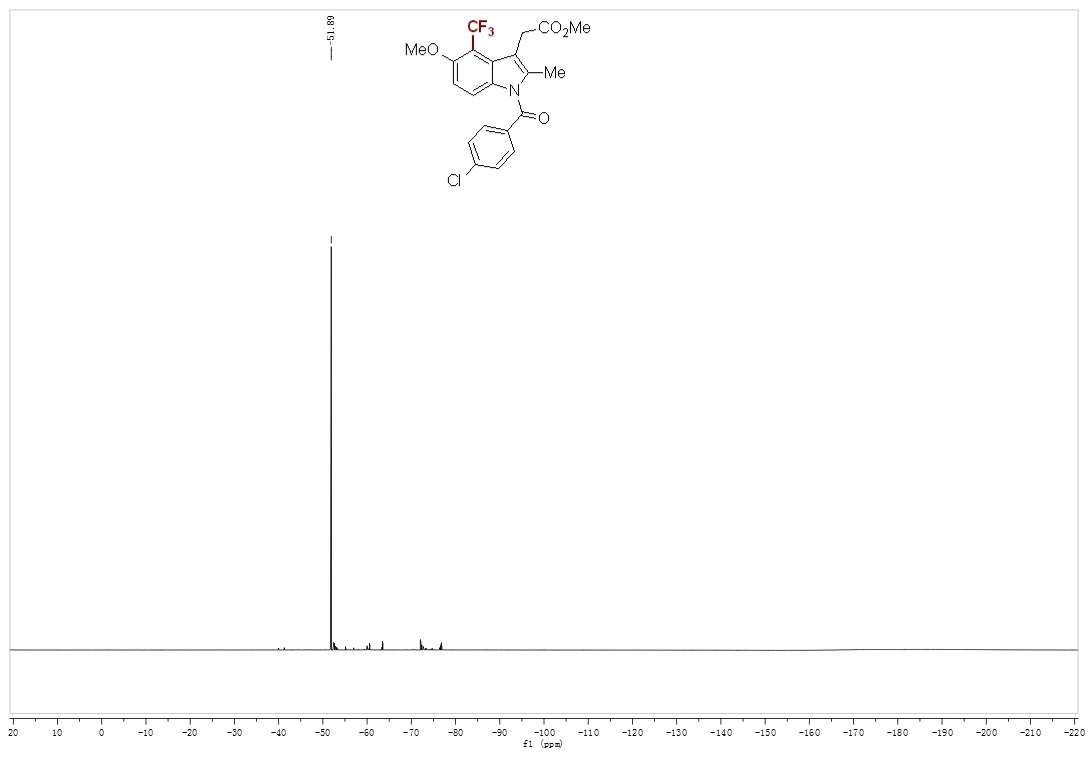


Supplementary Figure 82. ^19^F-NMR spectra of compound 2o.


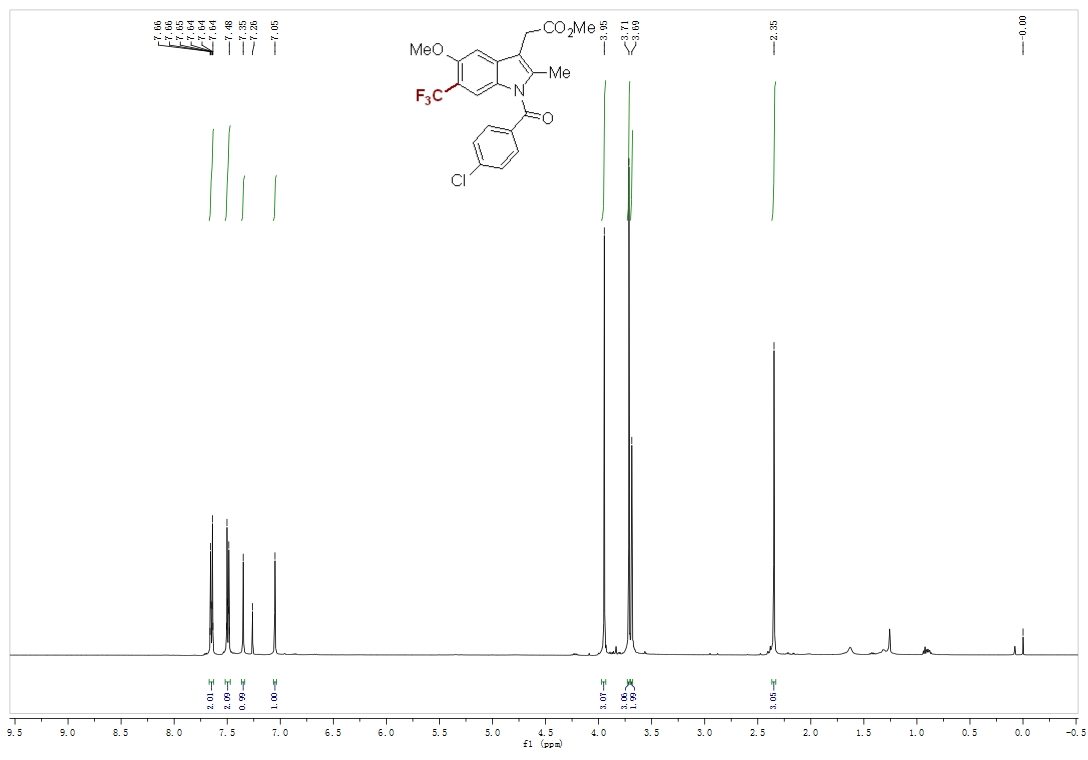


Supplementary Figure 83. ^1^H-NMR spectra of compound 2o'.


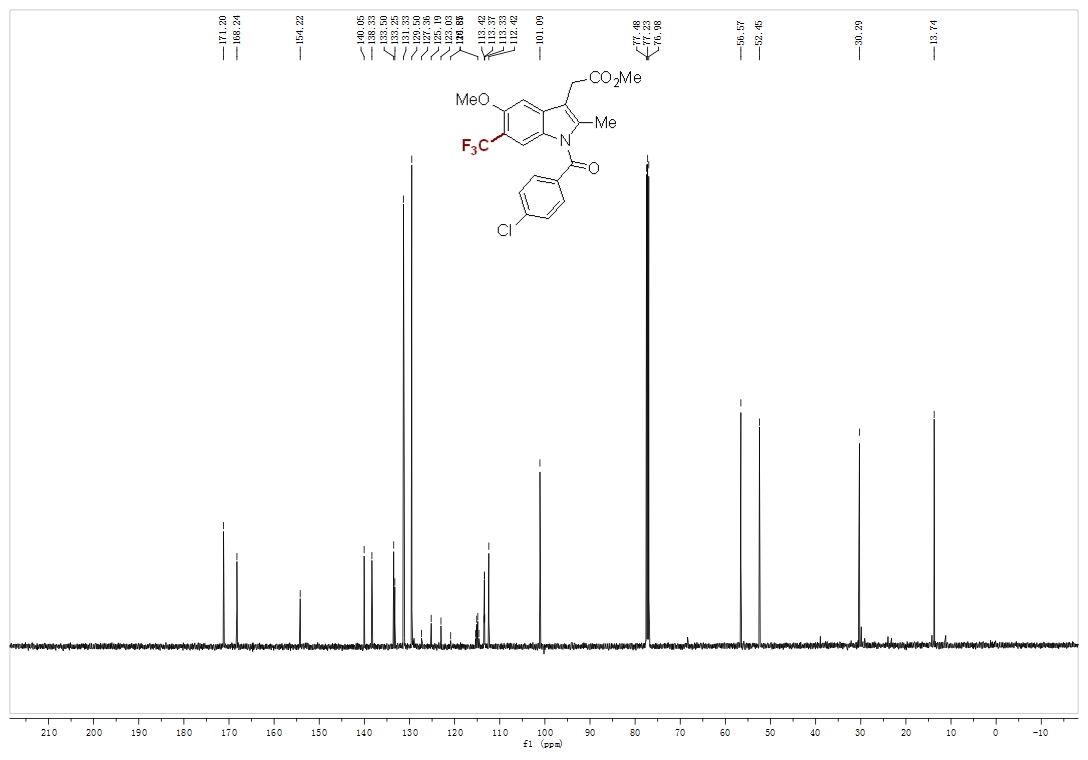


Supplementary Figure 84. ^13^C-NMR spectra of compound 2o'.


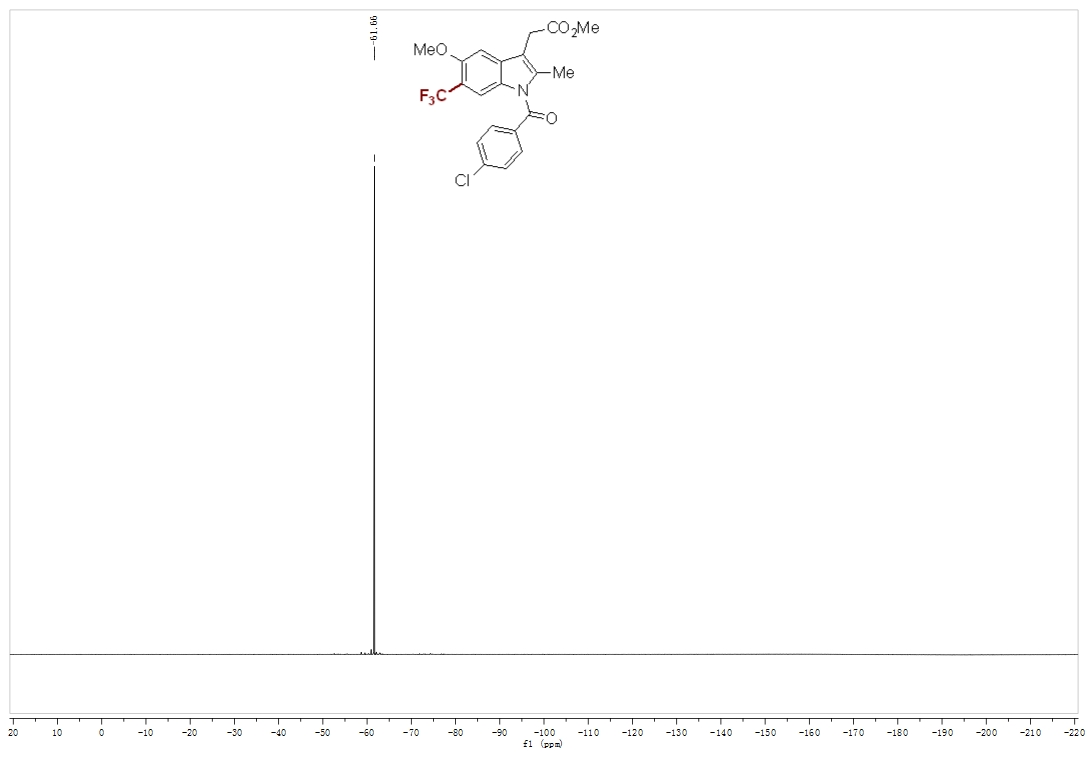


Supplementary Figure 85. ^19^F-NMR spectra of compound 2o'.


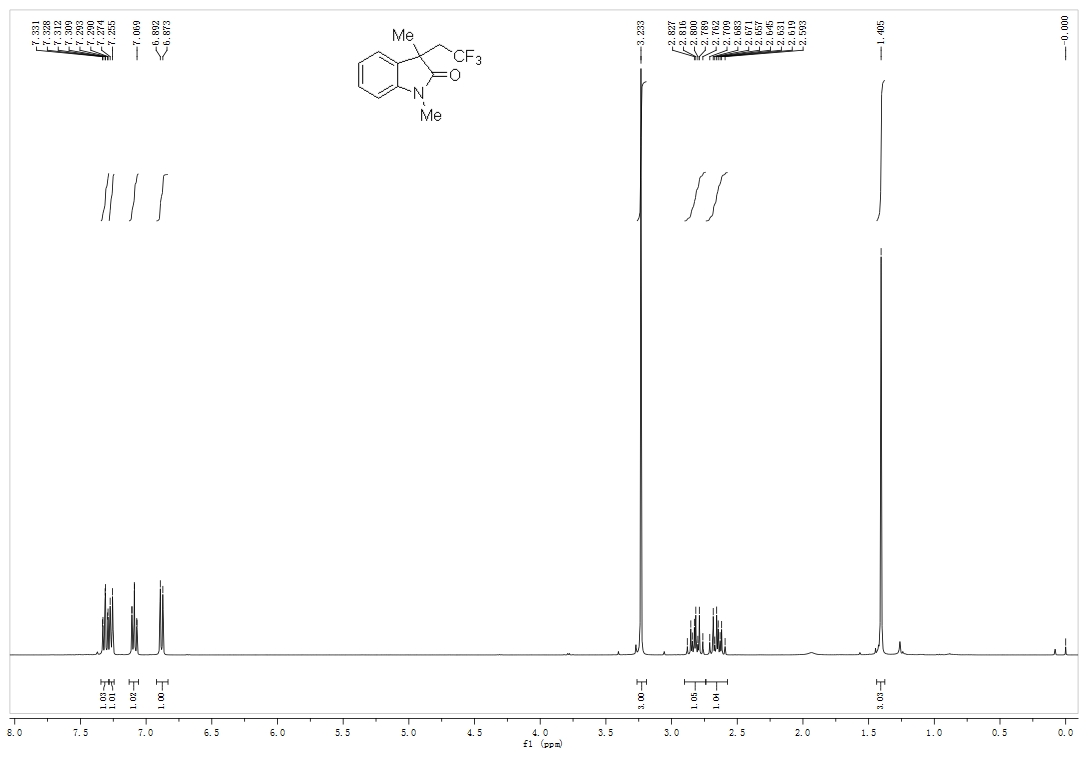


Supplementary Figure 86. ^1^H-NMR spectra of compound 4a.


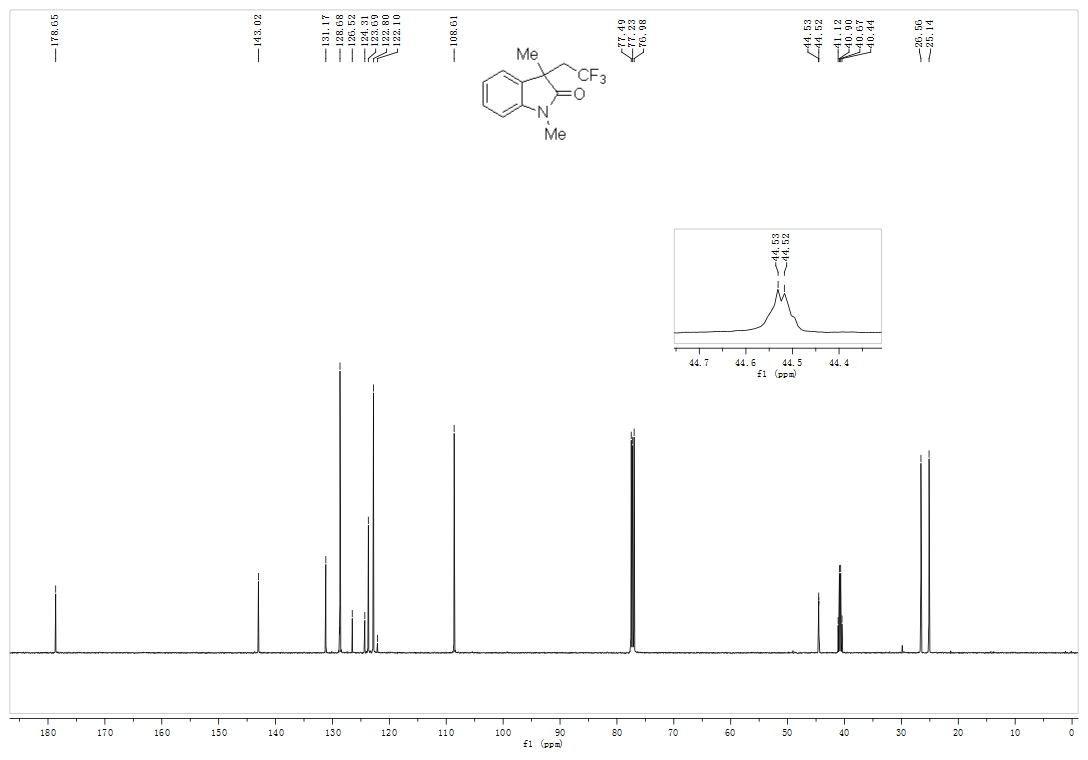


Supplementary Figure 87. ^13^C-NMR spectra of compound 4a.


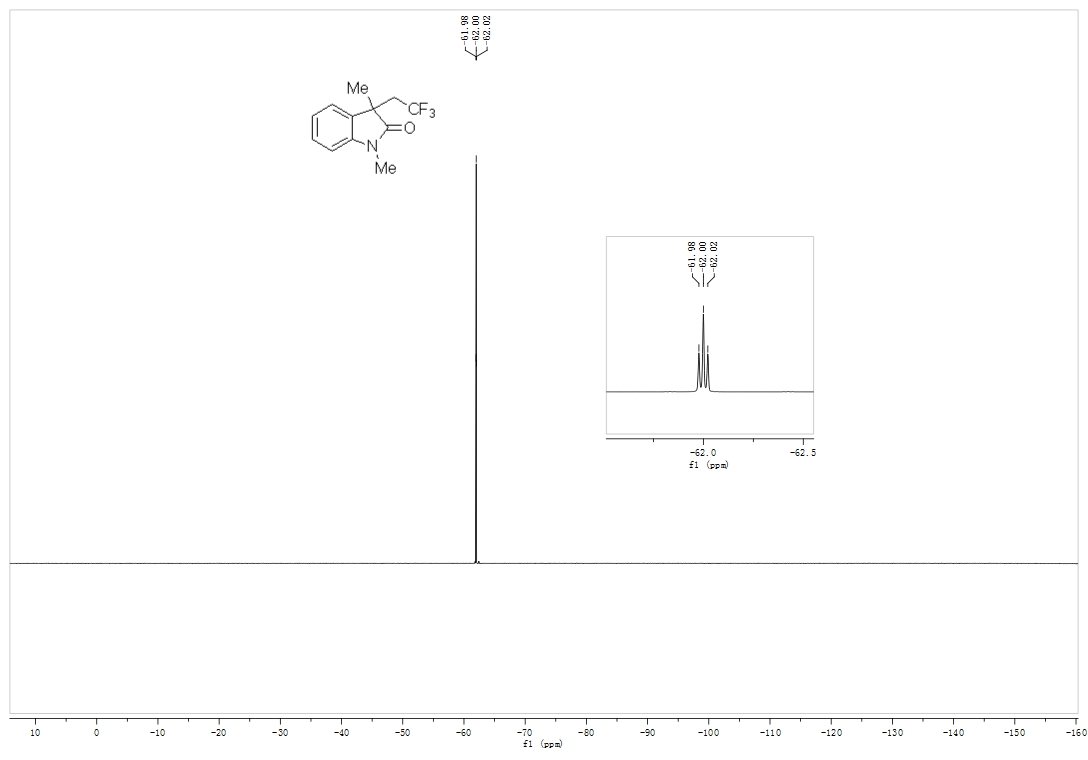


Supplementary Figure 88. ^19^F-NMR spectra of compound 4a.


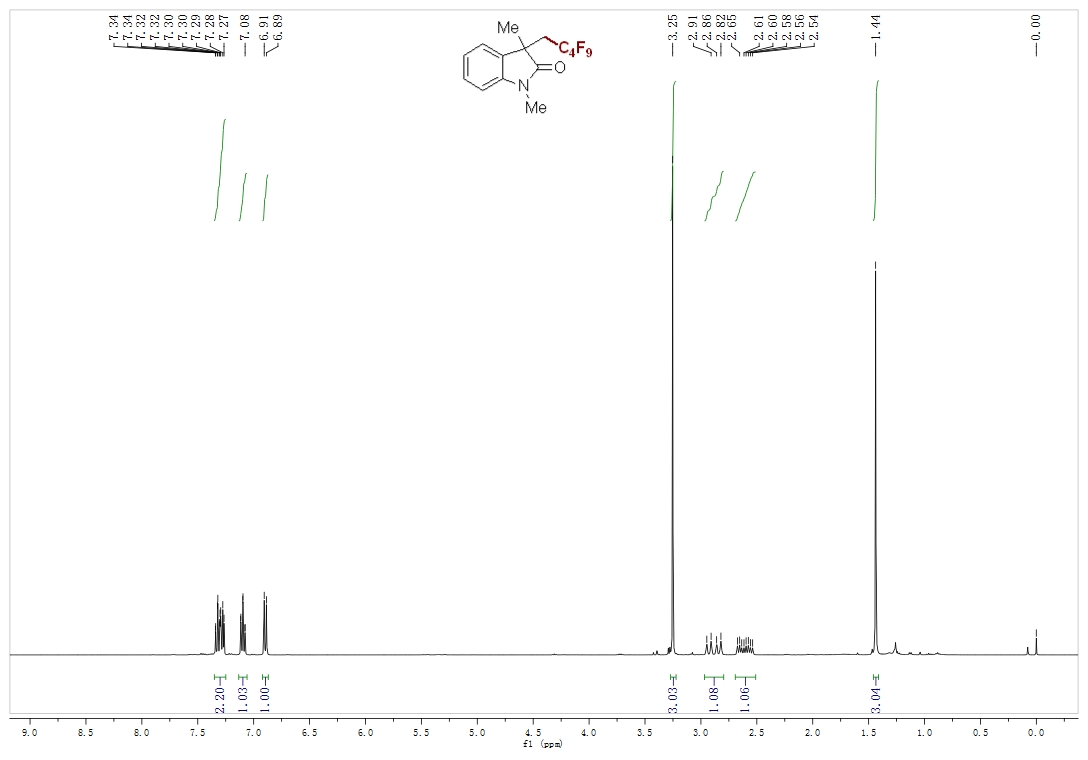


Supplementary Figure 89. ^1^H-NMR spectra of compound 4aa.


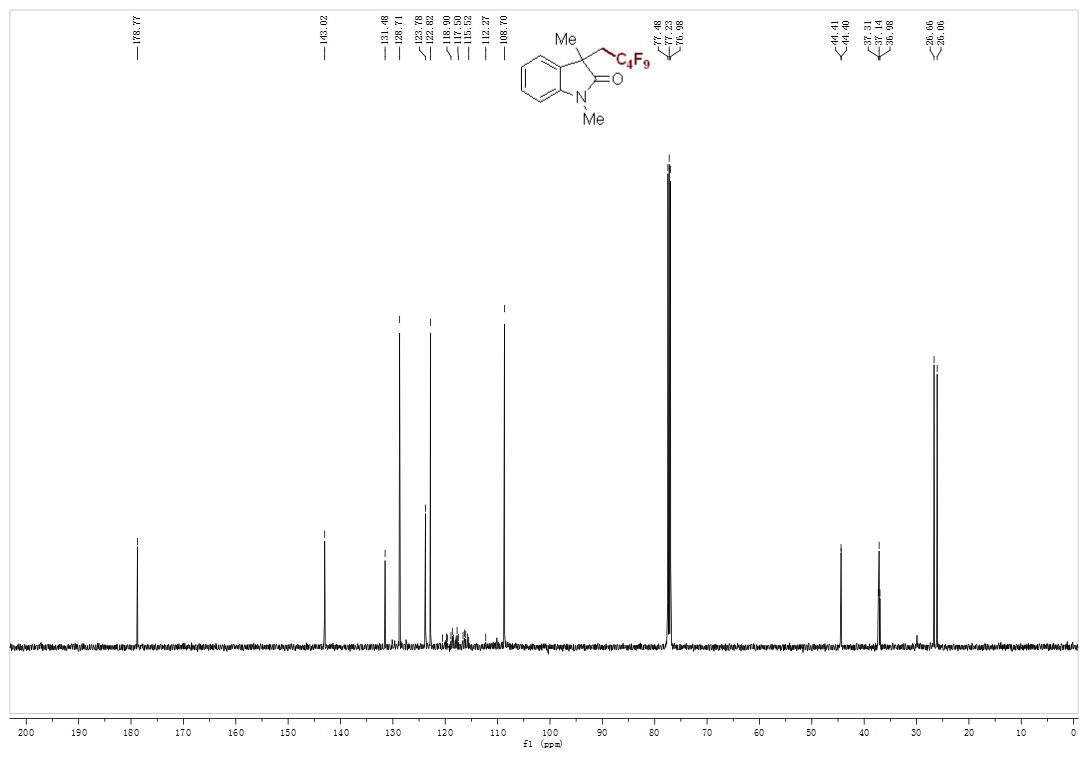


Supplementary Figure 90. ^13^C-NMR spectra of compound 4aa.


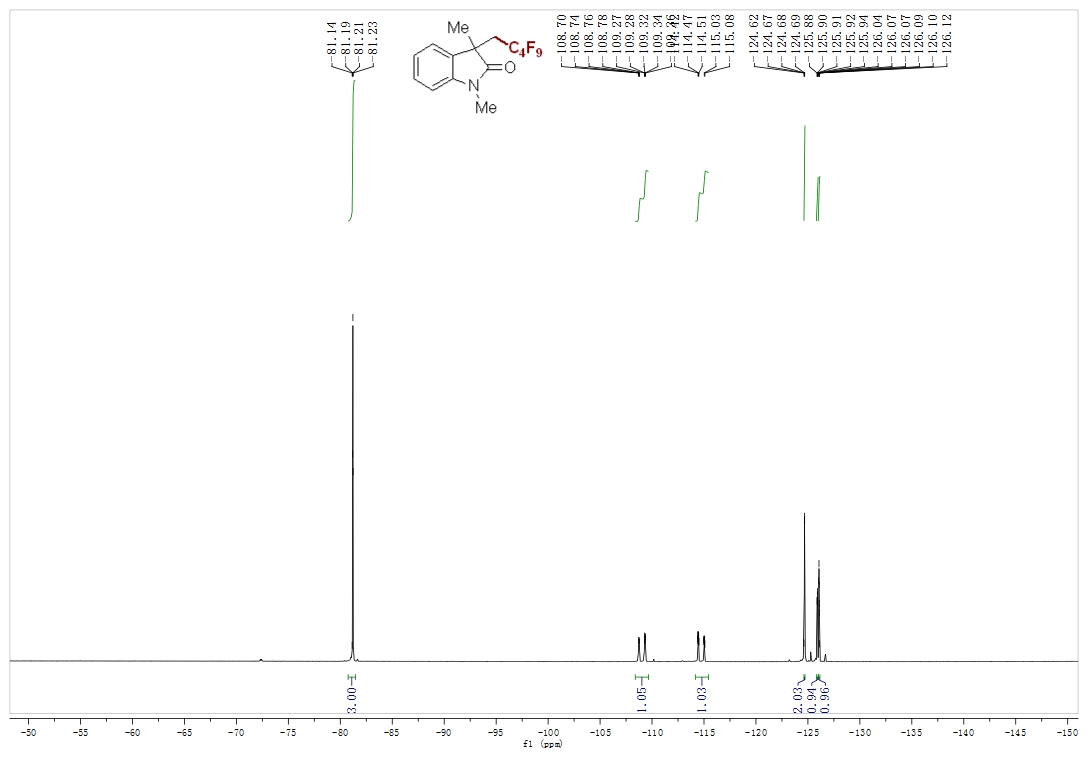


Supplementary Figure 91. ^19^F-NMR spectra of compound 4aa.


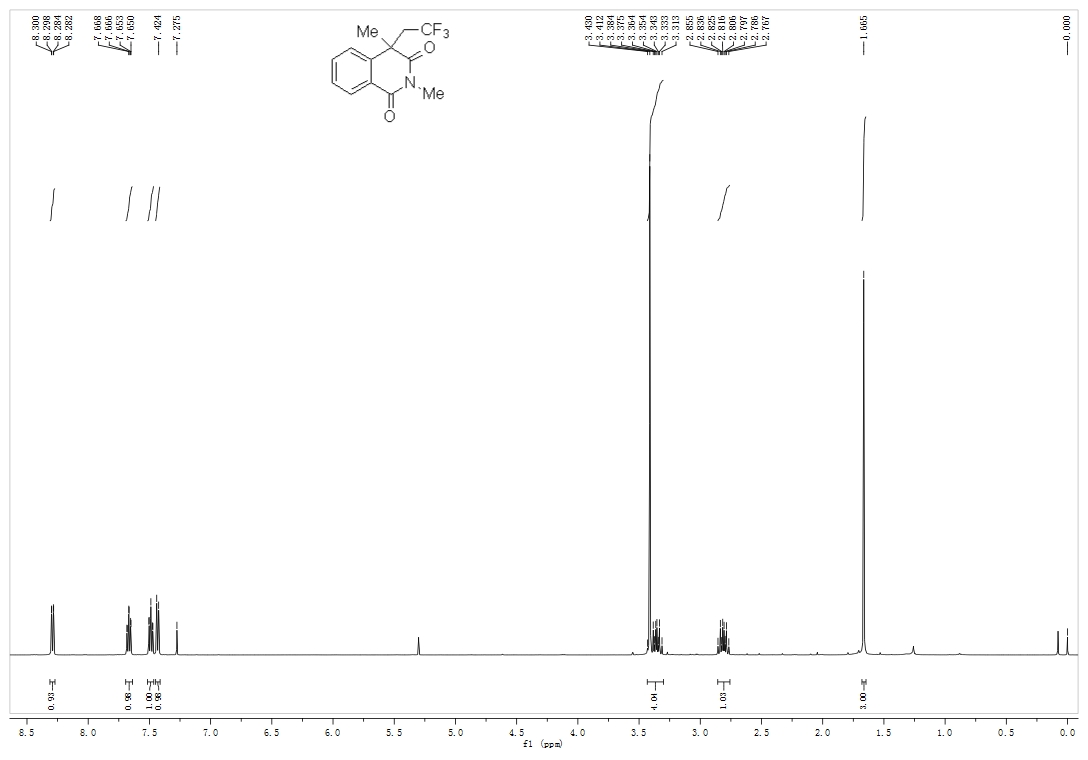


Supplementary Figure 92. ^1^H-NMR spectra of compound 4b.


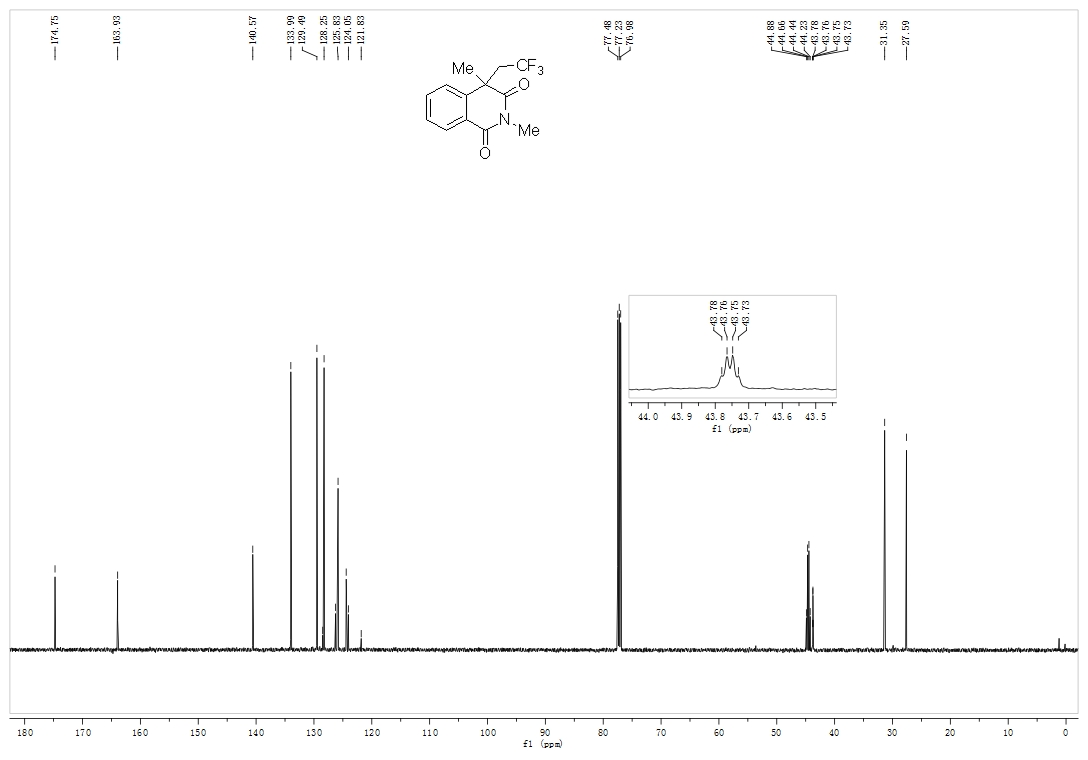


Supplementary Figure 93. ^13^C-NMR spectra of compound 4b.


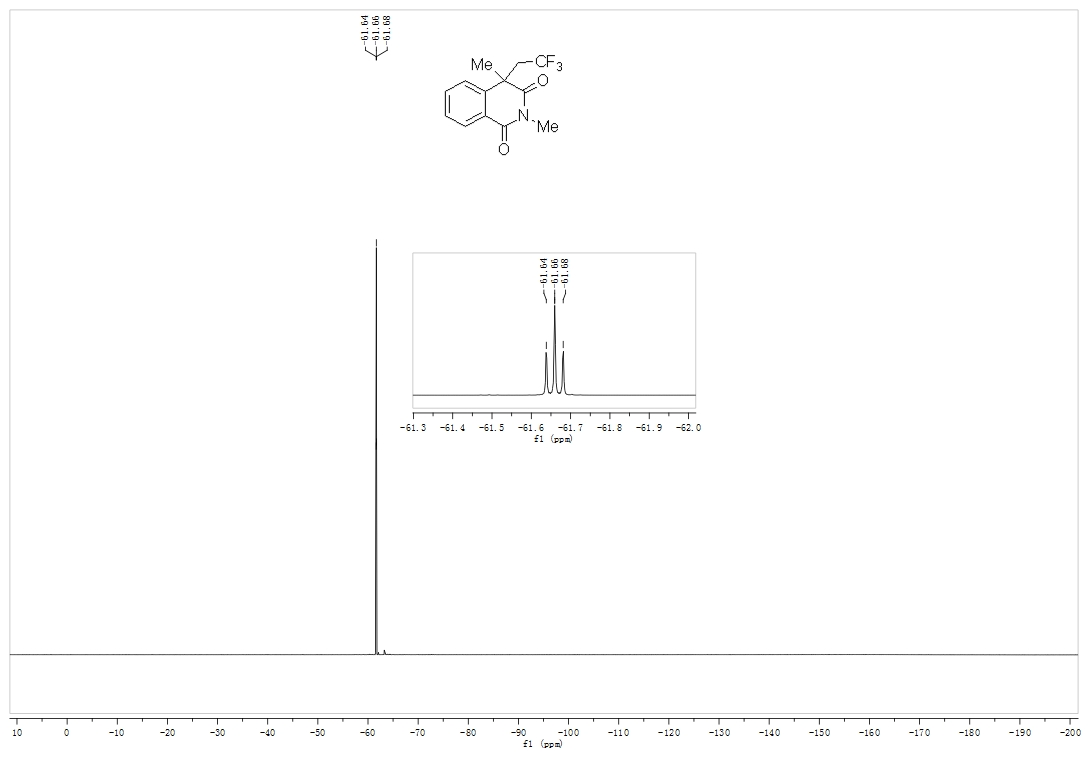


Supplementary Figure 94. ^19^F-NMR spectra of compound 4b.


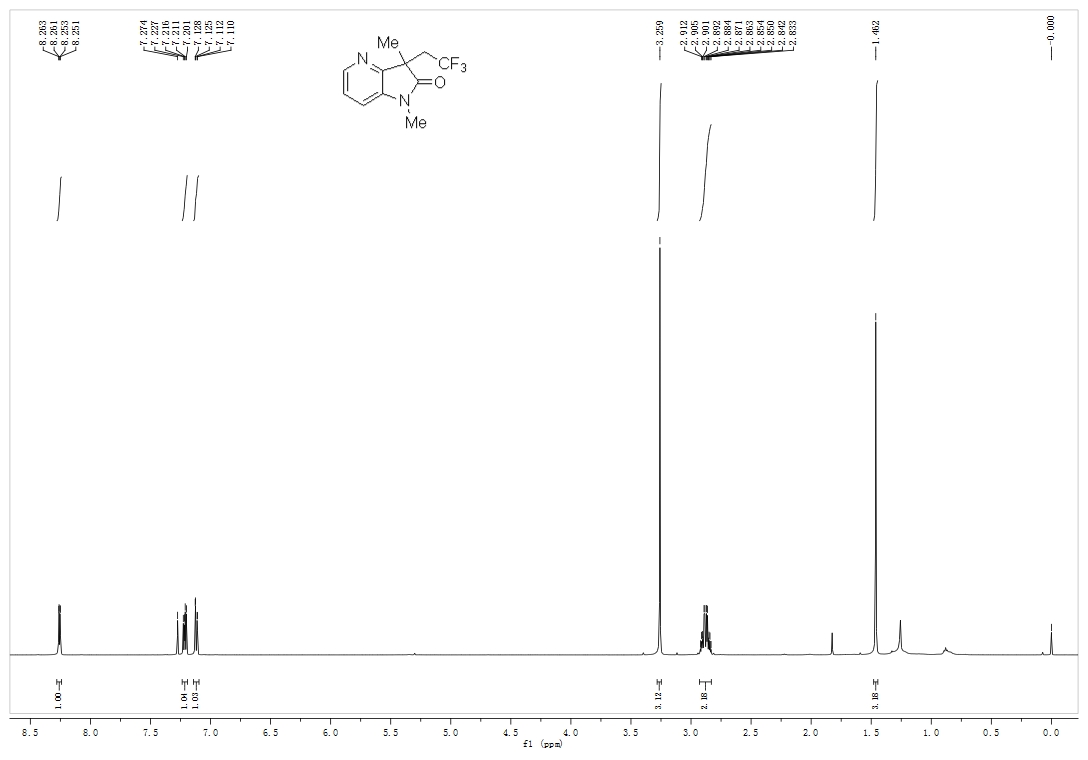


Supplementary Figure 95. ^1^H-NMR spectra of compound 4c.


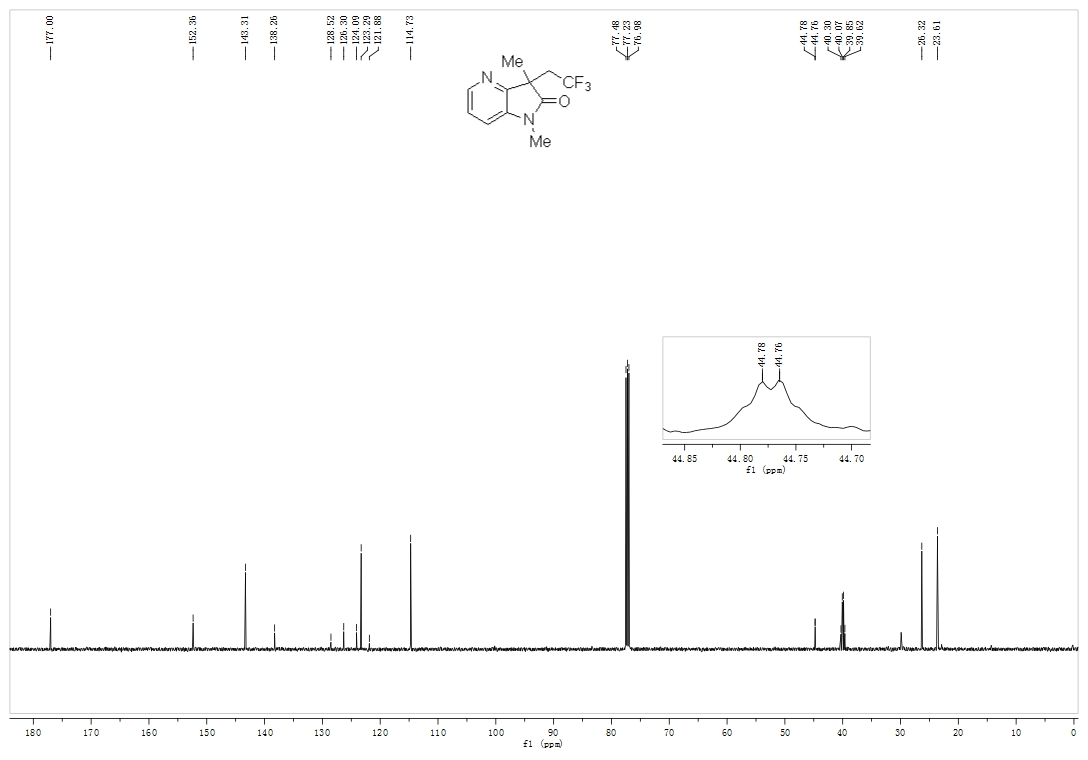


Supplementary Figure 96. ^13^C-NMR spectra of compound 4c.


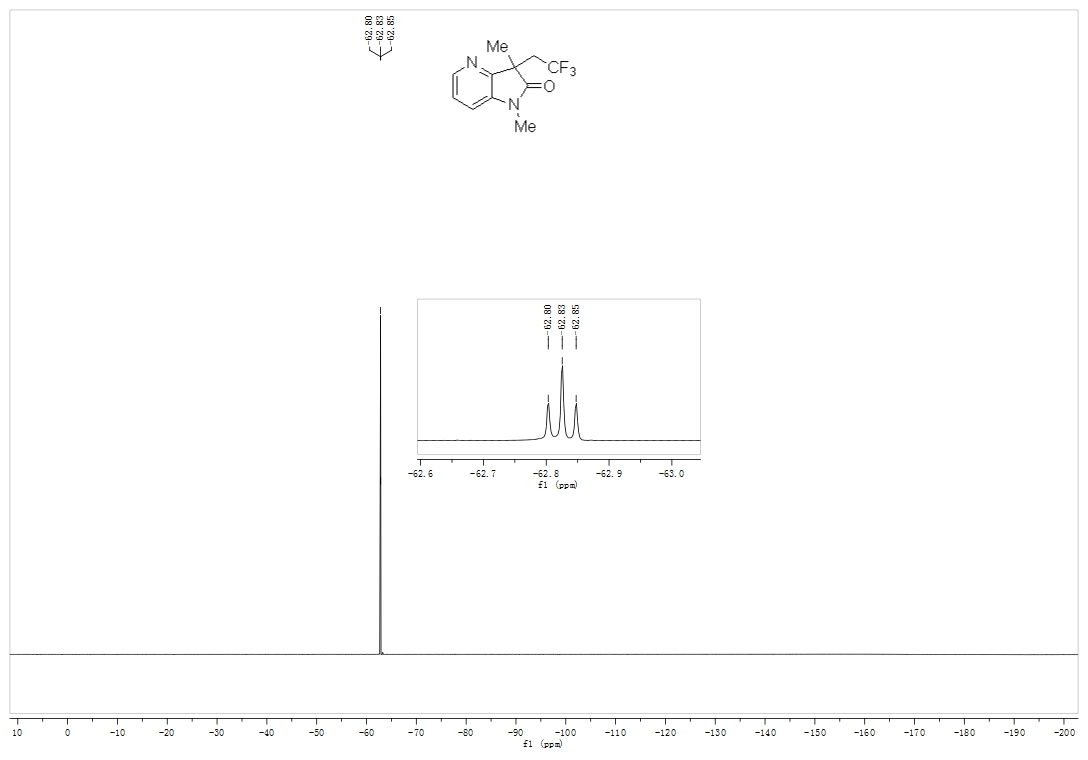


Supplementary Figure 97. ^19^F-NMR spectra of compound 4c.


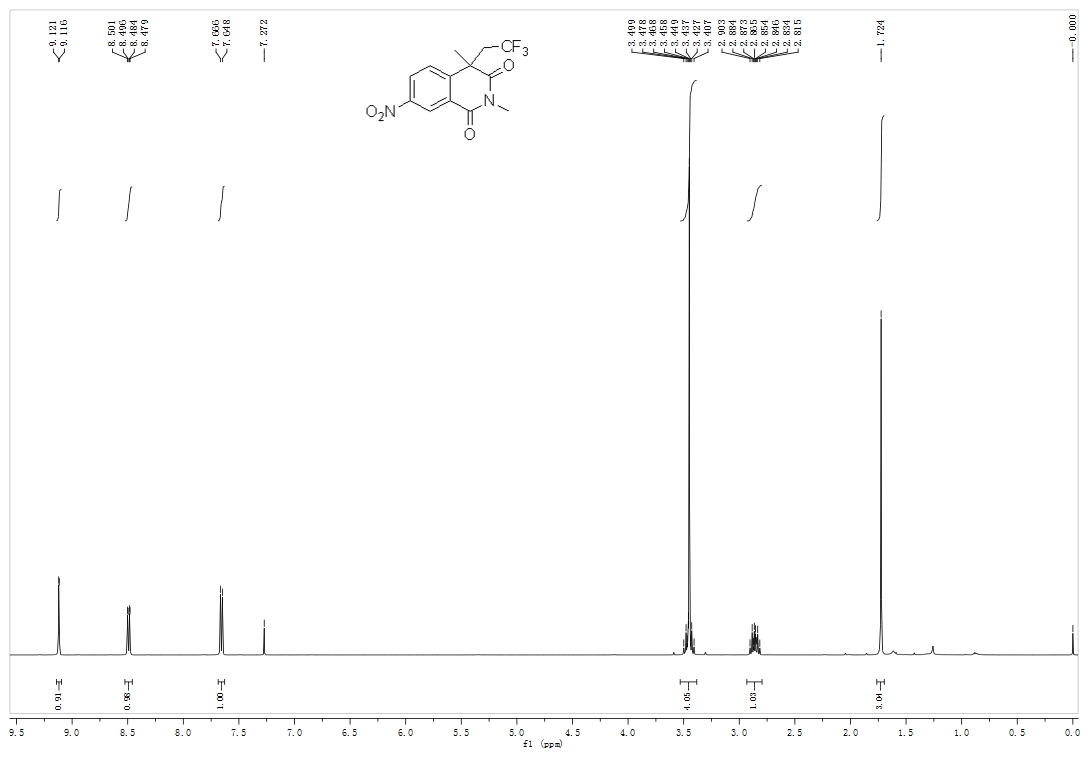


Supplementary Figure 98. ^1^H-NMR spectra of compound 4d.


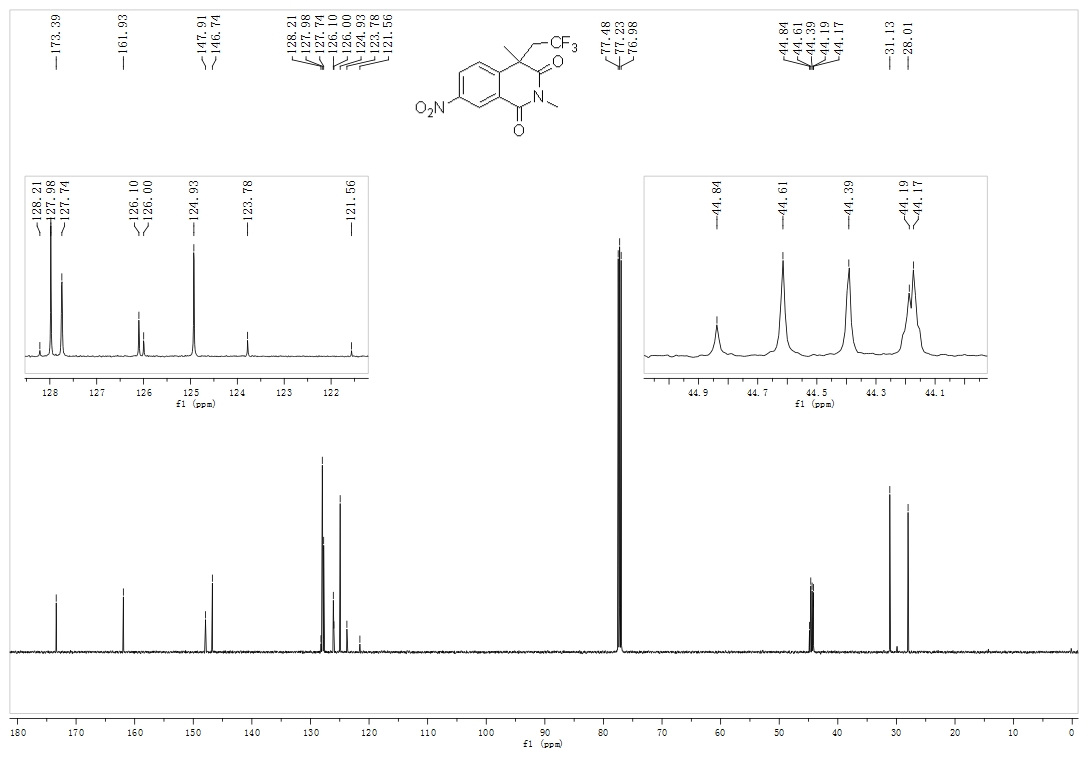


Supplementary Figure 99. ^13^C-NMR spectra of compound 4d.

Supplementary Figure 100. ^19^F-NMR spectra of compound 4d.

Supplementary Figure 101. ^1^H-NMR spectra of compound 4e.

Supplementary Figure 102. ^13^C-NMR spectra of compound 4e.

Supplementary Figure 103. ^19^F-NMR spectra of compound 4e.

Supplementary Figure 104. ^1^H-NMR spectra of compound 4f.

Supplementary Figure 105. ^13^C-NMR spectra of compound 4f.

Supplementary Figure 106. ^19^F-NMR spectra of compound 4f.

Supplementary Figure 107. 2D-NOESY-NMR spectra of compound 4f.

Supplementary Figure 108. ^1^H-NMR spectra of compound 4g.

Supplementary Figure 109. ^13^C-NMR spectra of compound 4g.

Supplementary Figure 110. ^19^F-NMR spectra of compound 4g.

Supplementary Figure 111. 2D-NOESY-NMR spectra of compound 4g.

Supplementary Figure 112. ^1^H-NMR spectra of compound 4h.

Supplementary Figure 113. ^13^C-NMR spectra of compound 4h.

Supplementary Figure 114. ^19^F-NMR spectra of compound 4h.

Supplementary Figure 115. 2D-NOESY-NMR spectra of compound 4h.

Supplementary Figure 116. ^1^H-NMR spectra of compound 4i.

Supplementary Figure 117. ^13^C-NMR spectra of compound 4i.

Supplementary Figure 118. ^19^F-NMR spectra of compound 4i.

Supplementary Figure 119. 2D-NOESY-NMR spectra of compound 4i.

Supplementary Figure 120. ^1^H-NMR spectra of compound 4j.

Supplementary Figure 121. ^13^C-NMR spectra of compound 4j.

Supplementary Figure 122. ^19^F-NMR spectra of compound 4j.

Supplementary Figure 123. 2D-NOESY-NMR spectra of compound 4j.

Supplementary References.

1. Min, J. et al. Effects of oligothiophene π-bridge length on physical and photovoltaic properties of star-shaped molecules for bulk heterojunction solar cells. *J. Mater. Chem. A* **2**, 16135–16147 (2014).
2. SMART Data collection software (version 5.629) (Bruker AXS Inc., Madison, WI, 2003).
3. SAINT, Data reduction software (version 6.45) (Bruker AXS Inc., Madison, WI, 2003).
4. Sheldrick, G. M. *SHELX-97, Program for Crystal Structure Analysis* (University of Göttingen, Göttingen, 1997).
5. Kalgutkar, A. S. et al. Ester and Amide Derivatives of the Nonsteroidal antiinflammatory drug, indomethacin, as selective cyclooxygenase-2 inhibitors. *J. Med. Chem.* **43**, 2860–2870 (2000).
6. Nagib, D. A. & MacMillan, D. W. C. Trifluoromethylation of arenes and heteroarenes by means of photoredox catalysis. *Nature* **480**, 224–228 (2011).
7. Natte, K. et al. Palladium-catalyzed trifluoromethylation of (hetero) arenes with CF_3_Br. *Angew. Chem. Int. Ed.* **55**, 2782–2786 (2016).
8. Cao, X.-H., Pan, X., Zhou, P.-J., Zou, J.-P. & Asekun, O. T. Manganese (iii)-mediated direct Csp^2^–H radical trifluoromethylation of coumarins with sodium trifluoromethanesulfinate. *Chem. Commun.* **50**, 3359–3362 (2014).
9. Straathof, N. J. W., Gemoets, H. P. L., Wang, X., Schouten, J. C., Hessel, V. & Noël, T. Rapid trifluoromethylation and perfluoroalkylation of five-membered heterocycles by photoredox catalysis in continuous flow. *ChemSusChem* **7**, 1612–1617 (2014).
10. Li, L., Mu, X., Liu, W., Wang, Y., Mi, Z. & Li, C.-J. Simple and clean photoinduced aromatic trifluoromethylation reaction. *J. Am. Chem. Soc.* **138**, 5809−5812 (2016).
11. Mu, X., Wu, T., Wang, H.-Y., Guo, Y.-L. & Liu, G. Palladium-catalyzed oxidative aryltrifluoromethylation of activated alkenes at room temperature. *J. Am. Chem. Soc.* **134**, 878–881 (2012).
12. Wang, H., Guo, L.-N. & Duan, X.-H. Palladium-catalyzed alkylarylation of acrylamides with unactivated alkyl halides. *J. Org. Chem.* **81**, 860−867 (2016).
13. Li, L., Deng, M., Zheng, S.-C., Xiong, Y.-P., Tan, B. & Liu, X.-Y. Metal-free direct intramolecular carbotrifluoromethylation of alkenes to functionalized trifluoromethyl azaheterocycles. *Org. Lett.* **16**, 504–507 (2014).
14. Xu, P., Xie, J., Xue, Q., Pan, C., Cheng, Y. & Zhu, C. Visible-light-induced trifluoromethylation of *N*-aryl acrylamides: a convenient and effective method to synthesize CF_3_-containing oxindoles bearing a quaternary carbon center. *Chem. Eur. J.* **19**, 14039–14042 (2013).
15. Kong, W., Casimiro, M., Fuentes, N., Merino, E. & Nevado, C. Metal-free aryltrifluoromethylation of activated alkenes. *Angew. Chem. Int. Ed.* **52**, 13086–13090 (2013).
16. Jiang, L., Yi, W. & Liu, Q. Direct phosphorus-induced fluoroalkylthiolation with fluoroalkylsulfonyl chlorides. *Adv. Synth. Catal.* **358**, 3700−3705 (2016).
