# Supplementary material for: Dye-incorporated coordination polymers for direct photocatalytic trifluoromethylation of aromatics at metabolically susceptible positions
Source: Nat Commun. 2018 Oct 2;9:4024. doi: 10.1038/s41467-018-05919-6 (PMC6168478; doi:10.1038/s41467-018-05919-6)
Supplement: Supplementary file 3 — Descriptions of Additional Supplementary Files [file 41467_2018_5919_MOESM3_ESM.pdf]

Descriptions of Additional Supplementary Files

File Name: Supplementary Data 1
Description: CIF of Zn−TCTA with a CCDC No. 1407818

File Name: Supplementary Data 2
Description: CIF of 1a@Zn−TCTA with a CCDC No. 1546691

File Name: Supplementary Data 3
Description: CIF of 3a@Zn−TCTA with a CCDC No. 1415189

File Name: Supplementary Data 4
Description: CIF of 3f@Zn−TCTA with a CCDC No. 1415190
